# Identification of a Novel Small Molecule That Enhances the Release of Extracellular Vesicles with Immunostimulatory Potency via Induction of Calcium Influx

**DOI:** 10.1021/acschembio.3c00134

**Published:** 2023-04-11

**Authors:** Yukiya Sako, Fumi Sato-Kaneko, Nikunj M. Shukla, Shiyin Yao, Masiel M. Belsuzarri, Michael Chan, Tetsuya Saito, Fitzgerald S. Lao, Helen Kong, Marina Puffer, Karen Messer, Minya Pu, Howard B. Cottam, Dennis A. Carson, Tomoko Hayashi

**Affiliations:** † Moores Cancer Center, University of California San Diego, 9500 Gilman Dr, La Jolla, California 92093-0809, United States; ‡ Department of Rheumatology, Graduate School of Medical and Dental Sciences, Tokyo Medical and Dental University (TMDU), Tokyo 113-8519, Japan; § The Herbert Wertheim School of Public Health and Longevity, University of California San Diego, La Jolla, California 92093-0901, United States

## Abstract

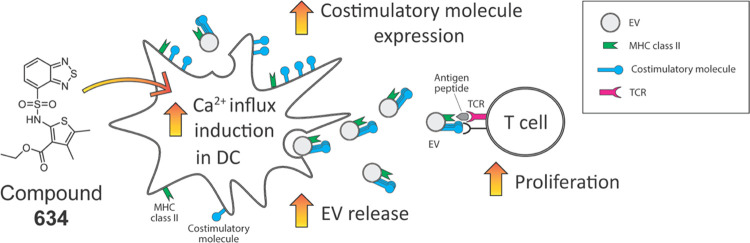

Extracellular vesicles (EVs) transfer antigens and immunomodulatory molecules in immunologic synapses as a part of intracellular communication, and EVs equipped with immunostimulatory functions have been utilized for vaccine formulation. Hence, we sought small-molecule compounds that increase immunostimulatory EVs released by antigen-presenting dendritic cells (DCs) for enhancement of vaccine immunogenicity. We previously performed high-throughput screening on a 28K compound library using three THP-1 reporter cell lines with CD63 Turbo-Luciferase, NF-κB, and interferon-sensitive response element (ISRE) reporter constructs, respectively. Because intracellular Ca^2+^ elevation enhances EV release, we screened 80 hit compounds and identified compound **634** as a Ca^2+^ influx inducer. **634** enhanced EV release in murine bone marrow-derived dendritic cells (mBMDCs) and increased costimulatory molecule expression on the surface of EVs and the parent cells. EVs isolated from **634**-treated mBMDCs induced T cell proliferation in the presence of antigenic peptides. To assess the roles of intracellular Ca^2+^ elevation in immunostimulatory EV release, we performed structure–activity relationship (SAR) studies of **634**. The analogues that retained the ability to induce Ca^2+^ influx induced more EVs with immunostimulatory properties from mBMDCs than did those that lacked the ability to induce Ca^2+^ influx. The levels of Ca^2+^ induction of synthesized analogues correlated with the numbers of EVs released and costimulatory molecule expression on the parent cells. Collectively, our study presents that a small molecule, **634**, enhances the release of EVs with immunostimulatory potency via induction of Ca^2+^ influx. This agent is a novel tool for EV-based immune studies and vaccine development.

## Introduction

Extracellular vesicles (EVs) act as carriers of cell-type-specific molecules, including those involved in innate immune responses, such as cytokines, chemokines, adhesion molecules, lipids, nucleic acids, coding and non-coding RNAs (including microRNAs), and DNA fragments.^1−5^ EV cargo can convey specific intercellular communications and mediate immune responses to microbial pathogens and tumors.^6−8^ Thus, EVs are a potential tool for vaccine adjuvant strategies.^9,10^

EVs derived from dendritic cells (DCs) may present on their surface the major histocompatibility (MHC) class I and II molecules and B7 costimulatory molecules, such as B7.1 (CD80) and B7.2 (CD86), which directly engage and activate CD4^+^ or CD8^+^ T cells.^11−14^ EVs also act as antigen-transferring/delivering tools. EVs from tumor cells can contribute to immunotherapy via delivering the tumor antigens to antigen-presenting cells.^15^ Circulating EVs from individuals who received mRNA-based vaccination for severe acute respiratory syndrome coronavirus 2 (SARS-CoV-2) were loaded with SARS-CoV-2 spike protein and induced spike protein-specific T cell responses and antibodies.^16^ EVs released from antigen-pulsed DCs or engineered EVs equipped with antigens can serve as artificial antigen-presenting particles.^10,17,18^ Thus, EVs are recognized as a next-generation vaccine platform because they function as cargo that transfers antigens and adjuvants and could be a promising strategy for enhancing vaccine efficacy.^9,10,19^

Intracellular Ca^2+^ influx is associated with both EV secretion and immune responses.^20−27^ Calcium signaling plays multiple roles in the activation, migration, and maturation of DCs that contribute to T cell priming and activation.^20,21^ Intracellular Ca^2+^ increment leads to plasma membrane EV biogenesis.^22−25^ Recent reports indicate that calcium ionophore ionomycin (ION) and A23187 enhance EV release^22,25,26^ and induce maturation and activation of antigen-presenting cells (APCs).^27^ However, these compounds are often toxic for in vivo utilization.^28^ Hence, we hypothesized that small-molecule compounds that can increase intracellular Ca^2+^ levels without cytotoxicity could enhance immune modulatory EV release from APCs.

To identify small molecules that increase EV release from APCs, we performed three independent high-throughput screenings (HTSs) on a 28K compound library with extensive chemical space diversity purchased from Maybridge (Leeds, United Kingdom).^29^ This library consists of two subset libraries that are representative of the diversity of the different compound collections, including the entire Maybridge Screening collection of more than 53,000 compounds and representative of the diverse collection of 550,000 compounds. We utilized a human monocytic leukemia THP-1 reporter cell line engineered with a fusion construct of EV-associated tetraspanin (CD63)-linked Turbo-luciferase (Tluc) (CD63 Tluc-CD9 EmGFP THP-1 reporter cells)^30^ and two additional THP-1 reporter cell lines for NF-κB and interferon-stimulated response element (ISRE) activation. Eighty hit compounds were identified after validation by *in vitro* functional screenings using murine bone marrow-derived dendritic cells (mBMDCs), *in vivo* immunization studies, and assessment from a medicinal chemistry perspective.^29^

Several reports indicate that manipulation of intracellular Ca^2+^ levels increases the number of EVs released.^22−25^ Thus, we screened the 80 hit compounds for the ability to induce Ca^2+^ influx and identified another hit compound ethyl 2-(benzo[*c*][1,2,5]thiadiazole-4-sulfonamido)-4,5-dimethylthiophene-3-carboxylate (hereafter designated as **634**) that triggers Ca^2+^ influx in mBMDCs. Compound **634** enhanced the number of EVs released and also costimulatory molecule expression on EVs. Purified EVs from **634**-treated mBMDCs promoted antigen-specific T cell proliferation. Moreover, focused structure–activity relationship (SAR) studies on **634** suggested that an increase in intracellular Ca^2+^ is closely associated with the immunostimulatory potency of EVs released by **634**-treated mBMDCs.

## Results

### Compound **634** Induces Ca^2+^ Influx

**Figure 1:**
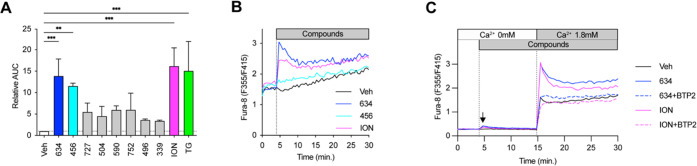
Compound **634** increases intracellular Ca^2+^ levels in mBMDCs. (A) Intracellular Ca^2+^ influx levels of the top eight compounds. THP-1 cells were loaded with the ratiometric Ca^2+^ indicator, Fura-2, and treated with ION (1 μM), TG (1 μM), or test compounds (5 μM). The time–response pattern of intracellular Ca^2+^ levels was recorded for 25 min. Area under the curve (AUC) of OD340/380 ratios corresponds to the intracellular Ca^2+^ kinetics, and the baseline-subtracted AUC was calculated by GraphPad Prism. Data presented are relative AUC to Veh (1.74 at 1st exp. and 1.06 ± 0.05 at 2nd exp. were set as 1, respectively) and mean ± SD of pooled data from three experiments showing similar results. **p < 0.01, ***p < 0.001 by one-way ANOVA with Dunnett’s *posthoc* test compared to Veh. (B) Ca^2+^ mobilization by compounds **456** and **634** in mBMDCs. mBMDCs were loaded with Fura-8 and treated with ION (1 μM), **634** or **456** (10 μM) for 25 min. The dashed line indicates the timing of compounds added. The data shown are representative of three independent experiments showing similar results. (C) Ca^2+^ add-back assay. Fura-8-loaded mBMDC were treated with ION, compound **634**, ION plus BTP2, or **634** plus BTP2 for 10 min in the absence of extracellular Ca^2+^, and then Ca^2+^ (final 1.8 mM) was added. ION, **634**, and BTP2 were added at final concentrations of 1, 10, and 5 μM, respectively. The data presented are averages of duplicates and representatives of two independent experiments showing similar results.

Our previous work demonstrated that small-molecule Ca^2+^ channel activators used as a coadjuvant enhance vaccine adjuvant activity.^31,32^ Triggering Ca^2+^ influx rapidly increases intracellular Ca^2+^ concentration, which enhances plasma membrane EV biogenesis.^22,23^ Thus, we postulated that small-molecule compounds that increase intracellular Ca^2+^ in APCs would enhance the release of immune stimulatory EVs. Eighty hit compounds selected by our triple HTSs were further analyzed by a ratiometric Ca^2+^ indicator assay in a human monocytic cell line (THP-1 cells) (Figure S1A). Ionomycin (ION) and thapsigargin (TG), a calcium ionophore and an inhibitor of sarco/endoplasmic reticulum Ca^2+^-ATPase, respectively, were used as positive controls.^31^ In a validation assay, compounds **634** and **456** significantly increased intracellular Ca^2+^ compared to the vehicle control (Veh, 0.5% DMSO) (Figures 1A and S1A,B). The top two hits, **634** and **456**, were further assayed for Ca^2+^ influx in primary mBMDCs using a ratiometric Ca^2+^ indicator. Compound **634** induced Ca^2+^ influx in mBMDCs, and the increase of intracellular Ca^2+^ was comparable to that of ION (1 μM), while **456** failed to elevate intracellular Ca^2+^ levels (Figure 1B). Compound **634** was nontoxic to mBMDC at 10 μM, which triggered Ca^2+^ influx. Thus, we selected compound **634** for further characterization.

Furthermore, we used the Ca^2+^ add-back assay in mBMDCs to seek the mechanism of intracellular Ca^2+^ increase by **634**. The store-operated Ca^2+^ entry (SOCE) is a major mechanism for Ca^2+^ import from extracellular to intracellular space in immune cells.^33^ SOCE is initiated by Ca^2+^ release from the endoplasmic reticulum (ER) stores and mediated by the interaction of the plasma membrane protein Orai and the ER membrane protein Stim. In the absence of extracellular Ca^2+^, **634** induced a small increase (black arrow in Figure 1C), which indicates a release of Ca^2+^ from the endoplasmic reticulum, similar to that of positive control for a SOCE inducer, ION (Figure 1C). When extracellular Ca^2+^ was replenished, **634** and ION treatment resulted in a sharp increase in Ca^2+^ via the SOCE. To confirm that **634**-induced Ca^2+^ influx was mediated by SOCE, we applied two SOCE inhibitors, BTP2 (also known as YM-58483, *N*-[4-[3,5-bis(trifluoromethyl)pyazol-1-yl]phenyl]-4-methylthiadiazole-5-carboxamide) and AnCoA4 (3-(6-methoxy-1,3-benzodioxol-5-yl)-8,8-dimethylpyrano[2,3-*f*]chromen-4-one, Orai1 channel inhibitor).^34,35^ Both compounds inhibited Ca^2+^ entry induced by **634** (Figures 1C and S2). These results indicated that Orai1-mediated SOCE predominantly contributes to the increase of intracellular Ca^2+^ levels induced by **634**.

An RNA-seq experiment was performed to assess the mechanism of action of **634**. The 5 h treatment time was chosen to assess early cellular responses and avoid possible indirect effects due to compound stimulation. Differential expression analysis at the gene level (R-limma, www.r-project.org) comparing **634** against the vehicle control in mBMDCs showed that among 103 genes whose expression was modulated, 7 genes related to calcium signaling were affected by **634** (5 genes upregulated and 2 downregulated) at Benjamini-Hochberg FDR≤0.05 and fold-change >2 (86 genes upregulated, 17 genes downregulated) (Table S1). To validate the RNA-seq data, the expression of the genes affected by **634** was further analyzed by reverse transcriptase-quantitative PCR (RT-qPCR) using ION as a positive control (Figure S3). The expression of five out of seven genes in **634**-treated cells, 5-hydroxytryptamine (serotonin) receptor 7 (Htr7), cadherin 1 (Cdh1), protein phosphatase 1E (Ppm1e), cadherin-related family member 1 (Cdhr1), and histamine receptor H1 (Hrh1), showed trends similar to the cells exposed to ION, implying that **634** acts via Ca^2+^ signaling pathways.

### **634** Increases EV Release

**Figure 2:**
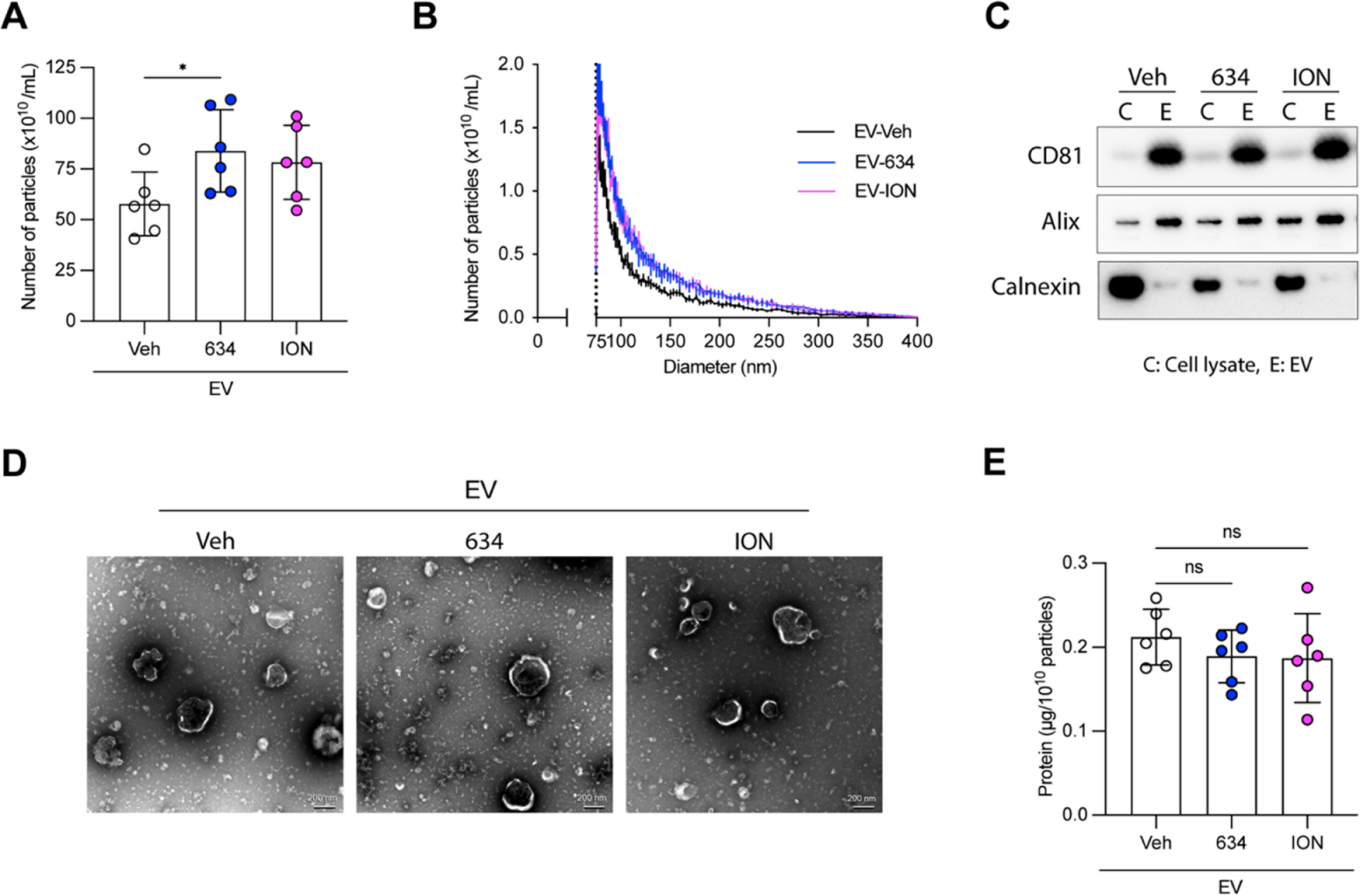
Compound **634** enhances EV release. mBMDCs were incubated with compound **634** (10 μM), ION (1 μM), or Veh (0.5% DMSO) for 48 h. EVs were isolated from the culture supernatant by differential ultracentrifugation, and final pellets were resuspended with 50 μL of PBS (designated as EV**_634_**, EV_ION_, and EV_Veh_). (A) Results were analyzed by MRPS, and the EV number was calculated per mL. Data shown are means ± SDs of EVs from six experiments using different mBMDC batches. *p < 0.05 by one-way ANOVA with Dunnett’s *posthoc* test vs Veh. (B) Size distributions of EV**_634_**, EV_ION_, and EV_Veh_ were measured by MRPS. Data shown are means ± SEMs of EVs from six different mBMDC batches. (C) Immunoblots of EVs (designated as “E”) and parental cell lysates (designated as “C”). The proteins (2 μg/well) were separated by 4–12% NuPAGE gel. Blots were probed with anti-CD81, anti-Alix, or anti-Calnexin antibodies. The images shown are representative of two independent experiments showing similar results. (D) Morphological examination of EV**_634_**, EV_ION_, and EV_Veh_ by TEM. Scale bars represent 200 nm. (E) Ratios of total protein amount to particles of EV**_634_**, EV_ION_, and EV_Veh_ were calculated per 10^10^ EV particles. The results were measured using the Micro BCA Assay kit. Experiments were repeated six times using individual mBMDC batches. Data shown are means ± SDs of data from six measurements. n.s., not significant by one-way ANOVA with Dunnett’s *posthoc* test vs Veh.

To confirm that **634** increased the number of EVs released in the culture supernatant, we measured the numbers of EVs from **634** (10 μM)-treated mBMDCs using microfluidic resistive pulse sensing (MRPS) with a Spectradyne nCS1 instrument. ION (1 μM) was used as a positive control.^25,36^ The EVs were isolated using a multistep differential ultracentrifugation protocol after 48 h treatment.^29^ The 48 h treatment time was chosen because EVs released by the vehicle-treated cells were detected only after 48 h based on the kinetics of EV secretion using CD63 Tluc reporter cells (data not shown). Compound **634** significantly increased the number of EVs released in the culture supernatant compared to that of the Veh control (0.5% DMSO) by 45% (*p* < 0.05, Figure 2A).

Since the heterogeneity of EVs often makes them difficult to obtain as relatively pure preparations and to characterize properly, the International Society for Extracellular Vesicles proposed Minimal Information for Studies of Extracellular Vesicles (“MISEV”) 2018 guidelines recommend the characterization of purified EVs by global quantification, e.g., particle number, total protein amount, and by single vesicle analysis.^37^ Thus, we characterized EV preparations (EV_Veh_, EV_**634**_, and EV_ION_) according to MISEV2018 guidelines. The sizes of the majority populations of EV_Veh_, EV_**634**_, and EV_ION_ were less than 200 nm and thus characterized as small EVs (<200 nm), suggesting that **634** did not affect the heterogeneity of EVs (Figure 2B). EVs were further confirmed by their specific protein composition showing enrichment of the EV markers, CD81 and Alix, and reduced amounts of a protein, calnexin, not associated with small EVs, as shown by immunoblots (Figure 2C and S4). The single vesicles of EV_Veh_, EV_**634**_, and EV_ION_ were morphologically similar by transmission electron microscopy (Figure 2D). The total protein amounts of EV_Veh_, EV_**634**_, and EV_ION_ were measured by the Micro BCA Assay kit to evaluate protein contamination in EV preparations. Total protein amounts of these preparations were comparable, indicating that the purity of EVs was similar among the EV_Veh_, EV_**634**_, and EV_ION_ (Figure 2E). Collectively, these data indicate that **634** increased the number of EVs released by mBMDCs and whose properties were verified according to the MISEV2018 guidelines.

### EV**_634_** Displays an Enhanced Expression of Costimulatory Molecules on the EV Surface

**Figure 3:**
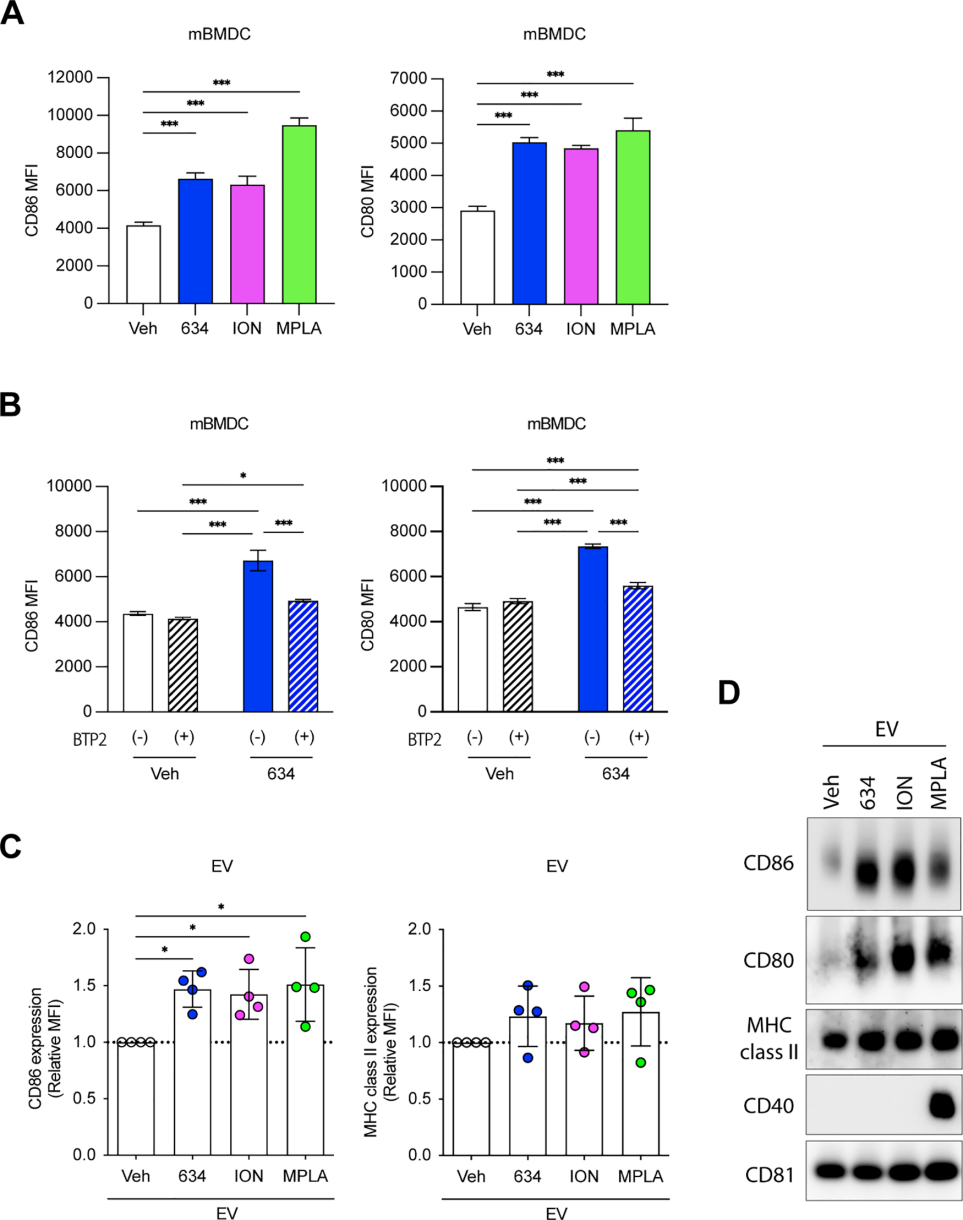
Compound **634** enhances costimulatory molecule expression on mBMDC and EV_**634**_. (A and B) After incubation with **634** (10 μM), ION (1 μM), MPLA (1 μg/mL), Veh (0.5% DMSO), BTP2 (5 μM), or **634** plus BTP2 for 20 h, mBMDCs were stained with a cocktail of anti-CD11c, anti-CD86, anti-CD80, anti-MHC class II, and anti-CD40 and analyzed by flow cytometry. The mean fluorescence intensity (MFI) is shown. Data shown are means ± SDs of triplicates representative of two independent experiments. (A) ***p < 0.001 by one-way ANOVA with Dunnett’s *posthoc* test vs Veh. (B) *p < 0.05, ***p < 0.001 by two-way ANOVA with Tukey’s *posthoc* test. (C) EV_Veh_, EV_**634**_, EV_ION_, and EV_MPLA_ were stained with a cocktail of vFRed, anti-CD86, and anti-MHC class II and analyzed by flow cytometry. Relative MFI to EV_Veh_ is shown. CD86: 58.4 ± 0.4 at 1st batch, 49.2 ± 0.2 at 2nd exp., 56.0 ± 0.5 at 3rd exp., and 63.1 ± 3.7 at 4th exp. and MHC class II: 416.5 ± 57.3 at 1st exp., 556.5 ± 44.5 at 2nd exp., 711.5 ± 16.3 at 3rd exp., and 559.0 ± 14.1 at 4th exp. were set as 1, respectively. Each dot represents a data set from individual mBMDC batches. Data shown are means ± SDs of EVs from four different mBMDC batches. *p < 0.05 by one-way ANOVA with Dunnett’s *posthoc* test vs EV_Veh_. (D) Immunoblot of EVs. The protein (2 μg/well) was separated and probed with anti-CD86, anti-CD80, anti-MHC class II, anti-CD40, or anti-CD81 antibodies. The images shown are representative of two independent experiments with similar results.

T cell activation requires antigens displayed on APC interacting with costimulatory and MHC molecules.^13^ We and others reported that calcium signaling regulates APC function.^20,21^ In the context of EVs, costimulatory molecules such as CD86 and MHC class II are expressed on EVs from parent DCs.^13^ Thus, we hypothesized that **634** increases the expression of costimulatory molecules and MHC class II on mBMDCs that are subsequently transferred to the surface of released EVs. To test this notion, mBMDCs were treated with Veh (0.5% DMSO), **634** (10 μM), ION (1 μM), or monophosphoryl lipid A (MPLA, 1 μg/mL) overnight, and the expression levels of CD86, CD80, MHC class II, and CD40 on mBMDCs were analyzed by flow cytometry (Figures 3A and S5). The TLR4 ligand, MPLA, was used as a positive control.^38^**634** and ION, as well as MPLA, increased CD86 and CD80 expression on mBMDCs (Figure 3A). To test whether intracellular Ca^2+^ increase induced by **634** led to the enhanced expression of CD86 and CD80, BTP2 was used to inhibit SOCE-mediated Ca^2+^ influx. Incubation with BTP2 significantly suppressed CD86 and CD80 expression, indicating that SOCE-mediated Ca^2+^ influx contributes to costimulatory molecule expression enhanced by **634** (Figure 3B). We next evaluated costimulatory molecules on EVs using single-particle high-resolution flow cytometry, with an Amnis CellStream^39^ (Figure 3C), and by immunoblots (Figures 3D and S6). Higher levels of CD86 expression were detected on EV_**634**_ and EV_ION_, similar to those on EV_MPLA_ in comparison to EV_veh_ (*p* < 0.05). This upregulation was also confirmed by immunoblots of isolated EVs (Figures 3D and S6). The immunoblots showed that CD86 and CD80 expression was higher on EV_**634**_ than that on EV_Veh_. These results highlighted that higher costimulatory molecule expression on EV_**634**_ mirrored the increased expression in parental mBMDCs treated with **634**.

### EVs Derived from mBMDCs Treated with **634** Stimulate DO11.10 T Cell Proliferation

**Figure 4:**
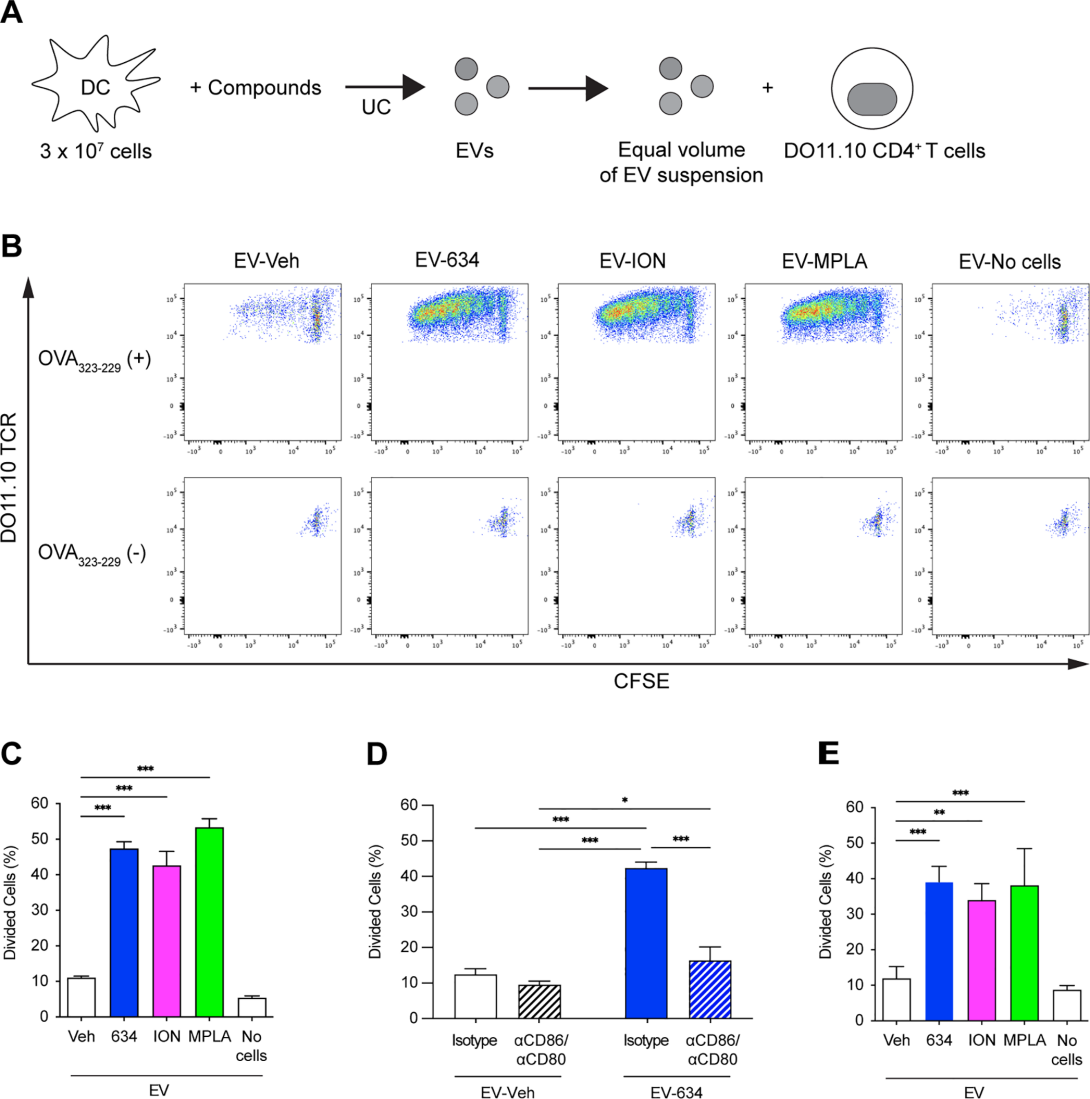
EV_**634**_ enhances T cell proliferation in the presence of antigenic peptides. (A, B) CFSE-labeled CD4^+^ T cells isolated from splenocytes of OVA TCR transgenic strain, DO11.10; splenocytes were treated with an equal volume (7 μL out of 50 μL) of the suspensions of EV_Veh_, EV_**634**_, EV_ION_, or EV_MPLA_ in the presence or absence of OVA_323-339_ peptide for 5 days. EV_No cells_ were used as a negative control. (C–E) T cell proliferation was determined by CFSE dilution using flow cytometry. Percentages of divided T cells induced by EVs from the same volumes of the culture supernatants and the number of parent cells. Data shown are means ± SDs of triplicates representative of two independent experiments. (C) In the presence of the OVA_323–339_ peptide, T cells were treated with an equal volume of the EV suspensions (7 μL out of 50 μL). (D) In the presence of the OVA_323-339_ peptide, T cells were treated with an equal volume (7 μL out of 50 μL) of the suspensions of EV_veh_ or EV_**634**_ in the presence of anti-CD86 and anti-CD80 antibodies or isotype controls for 5 days. Data shown are means ± SDs of triplicates representative of two independent experiments. (E) In the presence of the OVA_323–339_ peptide, T cells were treated with an equal particle number (3.13 × 10^9^). *p < 0.05, **p < 0.01, and ***p < 0.001 by one-way ANOVA with Dunnett’s *posthoc* test vs EV_Veh_ (C and E), and by two-way ANOVA with Tukey’s *posthoc* test (D).

The above data demonstrates that EV_**634**_ carried the costimulatory molecules CD86 and CD80 that are needed to prime naïve T cells during antigen presentation.^13^ To evaluate whether EV_**634**_ promotes T cell proliferation, we employed CD4^+^ T cells expressing ovalbumin (OVA)-specific T cell receptors (TCR) from DO11.10 mice that proliferate upon the engagement of TCR and OVA MHC class II peptides (OVA_323–339_)^40,41^ (Figure 4A). Carboxyfluorescein succinimidyl ester (CFSE)-labeled DO11.10 CD4^+^ T cells were cocultured with EV_**634**_ in the presence of OVA_323–339_. We used MPLA (1 μg/mL) as a positive control.^38^ EVs isolated from the medium without mBMDCs (EV_No cells_) served as negative controls. The amounts of EVs added to the T cell culture were normalized by the volumes of the culture supernatants and parent cell numbers. The proliferation of DO11.10 CD4^+^ T cells, as shown by CFSE dilution and IL-2 release into the culture supernatant, was monitored to evaluate the antigen-presenting function^42^ (Figures 4B, 4C, and S7A). In the presence of OVA_323–339_, EV_**634**_ induced significantly higher T cell proliferation and IL-2 release, equivalent to that of EV_MPLA_ or EV_ION_ (*p* < 0.001). In contrast, no T cell proliferation was detected by particles isolated from the medium (EV_No cells_) or in the absence of OVA_323–339_ (Figure 4B). These data indicate that EV_**634**_ stimulates T cell proliferation in a TCR-dependent manner.

### CD86 and CD80 on EV**_634_** Are Required for EV_634_-Enhanced T Cell Proliferation

The above data indicate that EV_**634**_ enhanced TCR-mediated T cell proliferation in the absence of APC. Because **634** enhanced the expression of costimulatory molecules on EV_**634**_ (Figure 3C, D), we hypothesized that CD86 and CD80 on EV_**634**_ contribute to the EV_**634**_ function. We used neutralizing antibodies for CD86 and CD80 to block the engagement of these molecules on EV to T cells during antigen presentation. The incubation with neutralizing antibodies significantly decreased T cell proliferation induced by EV_**634**_ to baseline levels (EV_veh_) (Figure 4D). These data indicate that the engagement of CD86 and CD80 on EV_**634**_ and T cells is required for TCR-dependent T cell activation.

In the above studies, the dosage of EVs was normalized by the volumes of culture supernatants and parent cell numbers.^38^ By this approach, we could not distinguish whether the induction of T cell proliferation was attributable to the increased EV number or to the immunostimulatory qualities of EVs. To address this question, an equal number of EVs (10^9^/mL) was cultured with CFSE-labeled DO11.10 CD4^+^ T cells. EV_**634**_ maintained higher levels of T cell proliferation and IL-2 release compared to EV_veh_ (*p* < 0.001) when equal numbers of EVs were used (Figures 4E and S7B). These data suggest that the higher particle number and the enhanced function of each EV_**634**_ particle both contributed to enhanced T cell proliferation. Collectively, **634** induced a higher number of EVs equipped with TCR-dependent T cell activation capacity, and both CD86 and CD80 on EV_**634**_ were associated with the T cell activation by EV_**634**_.

### Contaminants from the Culture Medium Have No Impact on EV_634_ Function

It is possible that the EV preparation may be contaminated with the components of the culture medium, namely, **634**, in the EV isolation process, referred to here as carryover **634**. To estimate the concentration of carryover **634**, we measured the level of **634** in an EV preparation using liquid chromatography–mass spectrometry and estimated the concentration to be 32 nM (Figure S8). To test whether carryover **634** in the EV preparation contributed to T cell proliferation, T cells were cultured with **634** alone (0.1, 1, and 10 μM) or **634** plus EV_veh_ (Figure S9). Neither treatment with **634** alone nor cotreatment with **634** and EV_veh_ affected T cell proliferation at as high as 10 μM, which is 300 times higher concentration than the estimated concentration of carryover **634** (32 nM). Other potential contaminants in the EV preparation are the high-molecular-weight complexes, i.e., aggregated proteins and other cellular components.^43^ To exclude these contaminants, we isolated EVs using the combination of ultracentrifugation and size-exclusion chromatography (SEC) and used them for the T cell proliferation assay. The combination method improves the purity of EVs by removal of non-EV-bound soluble proteins.^44,45^ EV particles concentrated by ultracentrifugation were further separated by SEC into two fractions: SEC-EV and SEC-contaminants (Figure S10A). SEC-EV_**634**_ induced significantly higher T cell proliferation compared to SEC-contaminants_**634**_ or EV_veh_, suggesting that the contaminants had a minimal impact on T cell proliferation (Figure S10B). These results indicate that EV_**634**_ contributes to T cell activation and that carryover **634** and the high-molecular-weight complexes in the EV preparation did not influence the EV_634_ function.

### Structure–Activity Relationship (SAR) Studies of 634

We demonstrated that EV_**634**_ could prime naïve T cells in a TCR-dependent manner. To confirm that Ca^2+^ influx is associated with immunostimulatory EV functions, we added to the mBMDC culture chelators for extracellular or intracellular calcium (EGTA and BAPTA-AM, respectively). However, these chelators were toxic and alone enhanced particle release from dead and dying cells. We, therefore, utilized focused SAR studies to obtain **634** analogues that had lost the ability to induce Ca^2+^ influx. We hypothesized that the potency is mediated by the chelation effects likely due to the resulting carboxylic acid functionality obtained by intracellular hydrolysis of the ester bond assisted by the presence of hydrogen-bonding atoms such as oxygen of the sulfonamide and nitrogen of the benzothiadiazole group.

**Figure 5:**
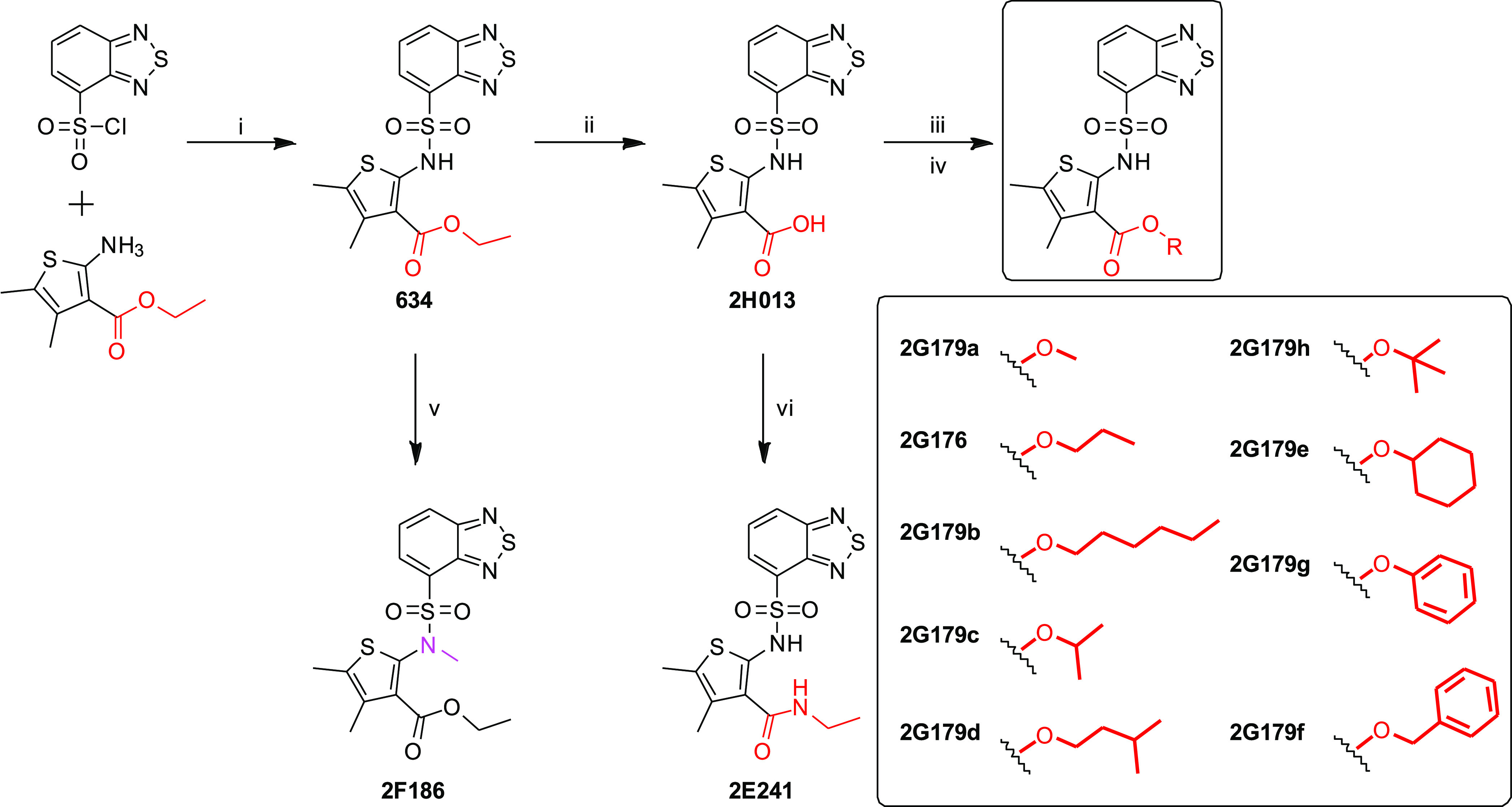
Focused SAR studies on **634**. Syntheses of twelve **634** analogues using a modification of the ester site of **634**.

Since compound **634** was not commercially available for bulk purchase, we first undertook its synthesis. Starting with ethyl 2-amino-4,5-dimethylthiophene-3-carboxylate with benzo[*c*][1,2,5]thiadiazole-4-sulfonyl chloride and pyridine as the base, compound **634** was obtained in good yields (44.5%). Next, using compound **634** as a common synthon, we first de-esterified the ethyl ester to obtain the free carboxylic acid analogue **2H013**. Then, the acid was converted to acid chloride with thionyl chloride to obtain an advanced reactive intermediate that was reacted with several different alcohol reagents to obtain ester-modified analogues. These included analogues with alkyl esters of varying chain lengths, such as **2G179a** (methyl ester), **2G176** (propyl ester), and **2G179b** (hexyl ester), and branched alkyl analogues, including **2G179c** (isopropyl ester), **2G179d** (isopentyl ester), **2G179h** (tert-butyl ester), and **2G179e** (cyclohexyl ester). Additionally, we also prepared analogues bearing phenolic (**2G179g**) and benzylic (**2G179f**) ester functionalities. An ethylamide analogue of **634** (**2E241**) was synthesized to probe the necessity of the ester functional group for Ca^2+^ mobilization. The sulfonamide group was also altered by *N*-methylation to obtain compound **2F186** (Figure 5).

### Ca^2+^ Influx Inducing 634 Analogues Release EVs That Promote T Cell Proliferation

**Figure 6:**
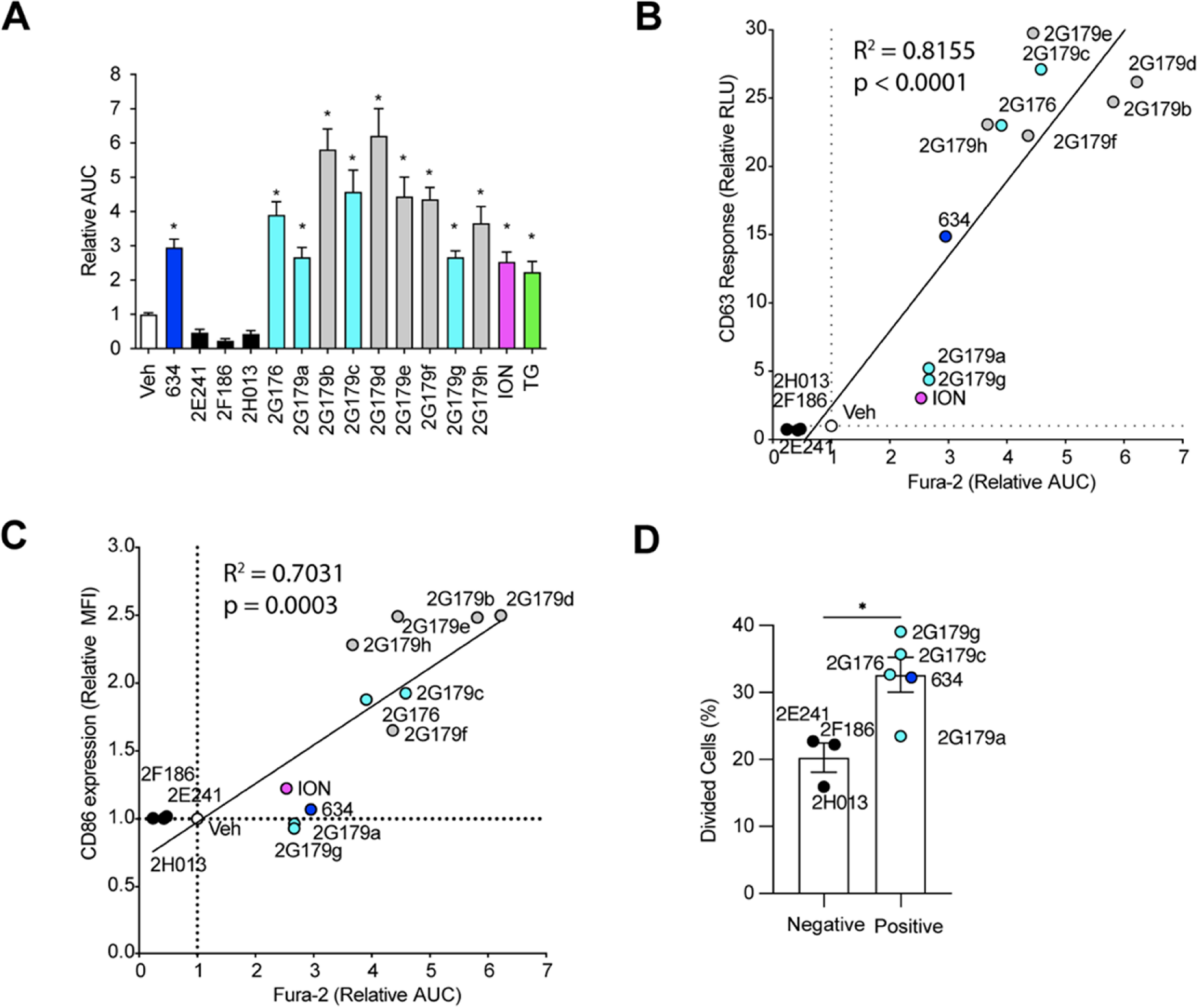
Correlation between intracellular Ca^2+^ influx and T cell proliferation by EVs from mBMDCs treated with 634 analogues. (A) Intracellular Ca^2+^ levels in mBMDCs were monitored following **634** analogue treatment. mBMDCs were loaded with Fura-2 and treated with **634**, its SAR analogues (10 μM), Veh (0.5% DMSO), ION (1 μM), or TG (1 μM). Data are presented as the normalized AUC (Veh was 1.00 ± 0.15). Data presented are means ± SDs of pooled two independent experiments performed in triplicate, showing similar results. *p < 0.05 by one-way ANOVA with Dunnett’s *posthoc* test compared to Veh. (B) Correlation analysis between intracellular Ca^2+^ induction (AUC) and the CD63 Tluc-CD9 EmGFP THP-1 reporter responses. Relative Luminescence activity to Veh (792.7 ± 84.58 at 1st exp. and 353.3 ± 29.14 at 2nd exp. were set as 1, respectively) is shown. (C) Correlation analysis between intracellular Ca^2+^ induction (AUC) and CD86 expression (MFI). mBMDCs were incubated with Veh, ION (1 μM), **634**, and its analogues (10 μM) for 24 h. MFI was normalized to Veh (1.00 ± 0.02). The fitted regression line is shown. Pearson correlation analysis for **634** and its SAR analogues was performed by Graphpad Prism 9. (D) CFSE-labeled DO11.10 CD4^+^ T cells were cultured with an equal volume of EV suspension (7 μL out of 50 μL) in the presence of the OVA_323–339_ peptide for 5 days. The dosage of EVs was normalized by the volumes of culture supernatants and parent cell numbers. Percentages of divided T cells relative to the original population are calculated. Each dot shown is the average of three independent experiments performed in triplicates, and data shown are means ± SEMs of negative compounds (**2E241**, **2F186**, and **2H013**) or five positive compounds (**634**, **2G176**, **2G179a**, **2G179c**, and **2G179g**), respectively. *p < 0.05 by the Mann–Whitney *U* test.

To examine the properties of the **634** analogues, we incubated mBMDCs with 12 of the compounds and followed intracellular Ca^2+^ levels (Figures 6A and S11) by the Fura-2 assay. Three analogues, **2E241** (amide analogue), **2F186** (*N*-methyl sulfonamide analogue), and **2H013** (carboxylic analogue), did not induce Ca^2+^ influx in mBMDCs according to the Fura-2 assay. The other nine analogues each statistically increased intracellular Ca^2+^ levels compared to Veh.

EV release and costimulatory molecule expression by mBMDCs treated with the **634** analogues were screened using CD63 Tluc reporter cells^30^ and by FACS in mBMDCs, respectively. Three analogues that lost the ability of Ca^2+^ influx, **2E241**, **2F186**, and **2H013**, did not increase luciferase activities in the culture supernatant of CD63 Tluc reporter cells nor enhance costimulatory expression on mBMDC. Furthermore, the levels of intracellular Ca^2+^ increase induced by the **634** analogues positively correlated with CD63 Tluc reporter cell responses and costimulatory molecule expression (*p* < 0.001, Figure 6B, C).

To confirm that Ca^2+^ influx is associated with immunostimulatory EV functions, we performed DO11.10 CD4^+^ T cell proliferation assays using EVs released by mBMDCs treated with **634** analogues. Due to the lower cell viability in exosome-depleted FBS medium, we excluded five agents (**2G179b**, **2G179d**, **2G179e**, **2G179f**, and **2G179h**) highlighted in gray in Figure 6A–C (Figures S12A,B).^46^ EVs were isolated from 48 h culture supernatants of mBMDCs treated with Ca^2+^ influx-positive (**634**, **2G176**, **2G179a**, **2G179c**, and **2G179g**) and -negative (**2E241**, **2F186**, and **2H013**) compounds. When T cell proliferation was expressed as relative values of divided cells (EV_veh_ = 1), EVs from mBMDCs treated with Ca^2+^ influx-positive compounds elicited significantly higher T cell proliferation than did Ca^2+^ influx-negative compounds (Figures 6D and S13). These results imply that the analogues that retain the ability to increase intracellular Ca^2+^ level induced higher immunostimulatory EV release than analogues that lost this ability and that ester and sulfonamide functional groups are necessary for the immunostimulatory function of EVs.

## Conclusions

Utilizing three parallel HTSs, we previously selected 80 hit compounds that enhance EV release with NF-κB activity and ISRE activity.^29^ In this study, the 80 hit compounds were screened for Ca^2+^ influx, and compound **634** was identified as a Ca^2+^ influx inducer mediated by SOCE. Compound **634** enhanced the number of EVs released by mBMDCs. This agent also increased costimulatory molecule expression on parental mBMDCs in a SOCE-dependent manner. EVs released from **634**-treated mBMDCs also had increased CD86 and CD80 expression compared to control EVs. The **634**-induced EVs markedly enhanced T cell proliferation in a TCR-dependent manner. Engagement of CD86 and CD80 on EV_**634**_ to T cells was required for the EV function. The SAR studies suggest that **634** analogues bearing the ester functional group retained the ability to induce Ca^2+^ influx and induced immunostimulatory EV release from mBMDCs compared to the amide, carboxylic acid, and *N*-methyl sulfonamide analogues, all of which lost the ability to induce Ca^2+^ influx. This agent and its analogues should be useful tools for the development of effective EV-based vaccines.

## Materials and Methods

### Reagents

Detailed information for the reagents is shown in Table S2.

### Compounds Synthesis

Compound **634** and 12 analogues were synthesized in our laboratory as described in the Supporting Information and Figure 5. These compounds were dissolved in DMSO (#D2438, MilliporeSigma, Temecula, CA) to obtain stock solutions (2 mM).

### Animals

Wild-type BALB/c and C57BL/6 mice and DO11.10 mice were purchased from the Jackson Laboratory. All animal experiments were approved by the Institutional Animal Care and Use Committee for UC San Diego.

### Generation of mBMDCs

mBMDCs were prepared from bone marrow cells harvested from femurs of BALB/c mice as previously described.^47,48^ mBMDCs were washed with RPMI 1640 medium and incubated with the compound or vehicle (0.5% DMSO) in RPMI 1640 supplemented with exosome-depleted FBS in a T182 flask (7.5 × 10^5^ cells/mL, total 40 mL) for 46–48 h. The culture supernatants were used for EV isolation.

### Cell Lines

THP-1 cells were cultured in RPMI 1640 medium supplemented with 10% dialyzed FBS supplemented with 100 U/mL penicillin, 100 μg/mL streptomycin, and 50 μM 2-mercaptoethanol. The CD63 Tluc reporter cell line^30^ were cultured in RPMI 1640 medium supplemented with 10% dialyzed FBS, 100 U/mL penicillin, 100 μg/mL streptomycin, 1 mM sodium pyruvate, 1 × MEM nonessential amino acids, and 5 μg of blastidin. Both cell types were maintained in humidified conditions with 5% CO_2_ at 37 °C.

### Calcium Influx Assay

Ca^2+^ influx was measured using Fura-2 or Fura-8 reagents. mBMDCs or THP-1 cells were loaded with ratiometric Ca^2+^ indicator, Fura-2-AM (4 μM) or Fura-8-AM (4 μM) in HBSS assay buffer [1× HBSS, 10 mM HEPES (pH 7.4), 1.8 mM CaCl_2_, 0.8 mM MgCl_2_, and 0.1% BSA], containing 0.04% Pluronic F127 at 37 °C for 40 min and at RT for additional 20 min. OD340/380 (emission) and OD510 nm (excitation) were read for Fura-2 by a fluorescence plate reader (Tecan2000, #30016056, TECAN, San Jose, CA). For Fura-8, OD 355/415 nm (excitation) and OD540 (emission) were read. Data were presented as OD ratios for 340/380 or 355/415 as a representative of changes in the intracellular Ca^2+^ level. The baseline-subtracted AUC of 340/380 ratios was calculated using GraphPad Prism (version 9, GraphPad Software, San Diego, CA) (Figure S14).

### Ca^2+^ Add-Back Assay

mBMDCs were loaded with Fura-8-AM in Ca^2+^-depleted HBSS assay buffer [1× HBSS, 10 mM HEPES (pH 7.4), 0.8 mM MgCl_2_, and 0.1% BSA] containing 0.04% Pluronic F127 at 37 °C for 40 min and at RT for additional 30 min. OD 355/415 nm (excitation) and OD540 (emission) were read. Data were presented as OD ratios for 340/380 or 355/415. Cells were treated with test compounds in the absence of Ca^2+^ first for 10 min and then CaCl_2_ was added up to 1.8 mM.

### EV Isolation by Differential Ultracentrifugation

EVs were isolated as described previously with minor modifications.^30^ Conditioned culture medium (40 mL) was spun at 300*g* for 10 min, at 2000*g* for another 10 min, and at 10,000*g* for 30 min. Next, 30 mL of supernatant was transferred to 31.5 polypropylene UC tubes and spun at 100,000*g*_avg_ for 3 h in an SW28 rotor (*K*-factor: 2554) by a Beckman Optima XL-90 ultracentrifuge (Beckman Coulter Life Sciences, Brea, CA). The supernatant was aspirated (leaving ∼50 μL), and the pellet was resuspended in 30 mL cold-filtered PBS. The resuspended pellet was spun under the same conditions as the previous spin, followed by another round of gentle aspiration and resuspension to a final volume of 50 μL in cold 0.02 μm filtered PBS. All centrifugation steps were performed at 4 °C, and the resulting samples were stored at −80 °C until use. All relevant data was submitted to the EV-TRACK knowledgebase (EV TRACK ID: EV220366, https://evtrack.org/index.php).^49^

### Measurement of EV Concentrations

EV particle numbers and size distributions were determined by the MRPS technique with an nCS1 particle analyzer utilizing C-400 cartridges (Spectradyne, Signal Hill, CA). EV samples were diluted 200-fold in 1% Tween20-filtered PBS. All results were analyzed using the nCS1 Data Analyzer (Spectradyne). The setting of the peak filters was “transit time (μs) from 0 to 100, symmetry from 0.2 to 4.0, and signal-to-noise ratio (S/N) of at least 10”. Particles below 75 nm were cut off to exclude the background particles from the diluent, 1% Tween20-filtered PBS (Figure S15), and particles with diameters from 75 to 400 nm were measured.

### Immunoblotting

Immunoblotting was performed using anti-CD81, anti-Alix, anti-Calnexin, anti-CD86, anti-CD80, anti-MHC class II, and anti-CD40 antibodies as primary antibodies as previously described by us.^30^ mBMDCs were lysed with PhosphoSafe extraction reagent supplemented with protease inhibitors. The total protein in the samples was quantitated by the Micro BCA Assay kit. Two micrograms of protein of cell or EV lysates was mixed with 4× NuPAGE sample buffer under reducing conditions with dithiothreitol (DTT) for Alix, Calnexin, CD86, CD80, MHC class II, and CD40 or nonreducing conditions (without DTT) for CD81. Samples were also denatured at 95 °C for 5 min prior to loading. After fractionation on NuPAGE 4–12% Bis-Tris gels, proteins were blotted onto Immobilon-P PVDF membranes and blocked for 1 h in 5% BSA-TBS-T at RT. The blots were then incubated with primary antibodies (Abs), anti-CD81, anti-Alix, anti-Calnexin, anti-CD86, anti-CD80, anti-MHC class II, and anti-CD40 Abs (1:1000 dilution), overnight at 4 °C with gentle agitation. After washing, the membranes were incubated with the corresponding secondary antibody for 30 min at RT with gentle agitation. Blots were developed with ProSignal Dura ECL and visualized using a ChemiDoc Imaging System. AccuRuler Prestained Protein Ladder was used for the molecular weight markers. Details for antibodies are shown in Table S3.

### Transmission Electron Microscopy

For the morphological characterization of EVs, negative stain transmission electron microscopy was performed as previously described.^30^ Formvar-carbon-coated copper grids (400 mesh, Ted Pella, Redding, CA) were placed on 10 μL drops of each sample solution displayed on a parafilm sheet. After allowing the material to adhere to the grids for 5 min, grids were washed three times by rinsing with more than 200 μL drops of milli-Q water before being left for 1 min on 2% (wt/vol) uranyl acetate in water. The excess solution was removed with 11 μm Whatman filter paper, and grids were left to dry for 20 min before viewing. Grids were examined using an FEI Tecnai Spirit G2 BioTWIN transmission electroscope equipped with a bottom-mount Eagle 4k (16 megapixels) camera (FEI, Hillsboro, OR).

### Costimulatory Molecule Expression Analysis

Costimulatory molecule expression on mBMDC was measured by the flow cytometry assay as described previously.^29^ mBMDCs (10^6^ cells/mL) were incubated with 10 μM compound, 1 μM Ionomycin, and 1 μg/mL MPLA for 20–24 h. DMSO (0.5%) was used as the vehicle. Cells were incubated with antimouse CD16/32 antibodies for blocking FcR and stained with anti- CD11c, anti-CD40, anti-CD80, anti-CD86, or anti-MHC class II antibodies for 30 min at 4 °C. Cells were stained with 4′,6-diamino-2-phenylindole (DAPI) for 10 min at RT. Data were acquired using MACSQuant Analyzer 10 (Miltenyi Biotec, Germany) and analyzed with FlowJo (version 10.8.1, Becton Dickinson, Ashland, OR). The gating strategy is shown in Figure S16.

### High-Resolution Single EV Analysis by Imaging Flow Cytometry

EVs were characterized using a commercially available reagent, vFC assay kit (#CBS, Cellarcus Biosciences, La Jolla, CA), using an Amnis CellStream Flow Cytometer equipped with 488 and 642 nm lasers (Luminex, Austin, TX) according to the manufacturer’s instructions^39^ with some modifications. In brief, samples were diluted 1:64 in vesicle staining buffer (Cellarcus Biosciences) and stained with an antibody cocktail of vFRed, anti-CD86, and anti-MHC class II Abs for 1 h at room temperature. Samples were diluted 1:200 in vesicle staining buffer before acquisition. Data were acquired using the Cellstream instrument with FSC and SSC turned off and all other lasers set to 100% of the maximum power. Each sample was run for 20 s at a sample volumetric flow rate of 3.66 μL/min. Relative MFI to EV_Veh_ is shown. Data were analyzed using FlowJo. Details for antibodies and flow cytometer configurations are shown in Table S3 and Figure S17.

### Antigen-Specific T Cell Proliferation Assay and ELISA

Transgenic OVA_323-329_ specific CD4^+^ cells were isolated from DO11.10 mice splenocytes using the EasySep Mouse CD4^+^ T cell isolation kit (negative selection). CFSE (4 μM)-labeled DO.11.10 CD4^+^ T cells were cocultured with an equal volume or equal number (3.13 × 10^9^ or 3.99 × 10^9^ EV particles) of EVs in the presence of the OVA_323–339_ peptide.^40^ In the assays with neutralization of CD86 and CD80, isotype control antibodies (rat IgG2a or American hamster IgG) or anti-CD86 and anti-CD80 antibodies (1.25 μg/mL) were added. IL-2 in the supernatant was tested by the Mouse IL-2 DuoSet ELISA kit. Cells were stained with antimouse DO11.10 clonotypic TCR antibody, and antigen-specific CD4^+^ T cell proliferation was evaluated by CFSE dilution using a MACSQuant flow cytometer (Meltyni Biotec, San Diego, CA). Cell proliferation was quantitated by the percentage of divided cells relative to the original population^42^ (Figure S18). Data were analyzed using FlowJo (version 10.8.1, FlowJo, Ashland, OR).

### CD63 Tluc-CD9 EmGFP THP-1 Reporter Cell Assay

The reporter cell assay was carried out as described previously.^29^ CD63 Tluc-CD9 EmGFP THP-1 reporter cells (designated as CD63 Tluc reporter cells) were incubated with 10 μM test compounds, ION (1 μM), or 0.5% DMSO (negative control) in RPMI 1640 supplemented with exosome-depleted FBS at 5 × 10^4^ cells/200 μL/well in a 96-well plate for 48 h at 37 °C. Subsequently, the plate was centrifuged, the supernatant was transferred, and chemiluminescence was measured by the TurboLuc Luciferase One-Step Glow Assay kit.

### Cell Viability Assay

The cell viability was measured by the MTT assay as described previously.^29^ mBMDCs (2 × 10^6^ cells/200 μL/well in RPMI 1640 supplemented with dialyzed 10% FBS or 1.5 × 10^6^ cells/200 μL/well in RPMI1640 supplemented with 10% exosome-depleted FBS) were treated with 10 μM of each test compound in 96-well plates. After 46–48 h of compound treatment, MTT (0.5 mg/mL) was added to each well. The cells were lysed after overnight incubation, and absorbance values at 570 and 650 nm were read.

### Statistical Analysis

To compare multiple groups, one-way ANOVA with Dunnett’s *posthoc* test or two-way ANOVA with Tukey’s *posthoc* test was used. To compare two groups, the two-tailed Mann–Whitney test was used. Prism 9 software (GraphPad Software, San Diego, CA) was used. *P* values smaller than 0.05 were considered statistically significant.

## Supporting Information Available

Additional data on cytokine levels, expression of costimulatory molecules, and cell viability; original data of the Ca^2+^ influx assay and immunoblotting; compound data of ^1^H NMR, ^13^C NMR, HRMS, and LC–MS analyses; and additional information about biological experiments (PDF)
