# Supplementary material for: Identification of a Novel Small Molecule That Enhances the Release of Extracellular Vesicles with Immunostimulatory Potency via Induction of Calcium Influx
Source: ACS Chem Biol. 2023 Apr 11;18(4):982–93. doi: 10.1021/acschembio.3c00134 (PMC10127211; doi:10.1021/acschembio.3c00134)
Supplement: Supplementary file 1 — cb3c00134_si_001.pdf [file cb3c00134_si_001.pdf]

Supporting Information

Identification of a Novel Small Molecule that Enhances the Release of Extracellular Vesicles with Immunostimulatory Potency via Induction of Calcium Influx

Yukiya Sako [1], Fumi Sato-Kaneko [1], Nikunj M. Shukla [1], Shiyin Yao [1], Masiel M. Belsuzarri [1], Michael Chan [1], Tetsuya Saito [1,2], Fitzgerald S. Lao [1], Helen Kong [1], Marina Puffer [1], Karen Messer [3], Minya Pu [3], Howard B. Cottam [1], Dennis A. Carson [1*†], Tomoko Hayashi [1*†]

[1] Moores Cancer Center, University of California San Diego, 9500 Gilman Dr, La Jolla, CA 92093-0809, United States

[2] Department of Rheumatology, Graduate School of Medical and Dental Sciences, Tokyo Medical and Dental University (TMDU), Tokyo, 113-8519, Japan

[3] The Herbert Wertheim School of Public Health and Longevity, University of California San Diego, La Jolla, CA, 92093-0901, United States

* Corresponding author: Dennis A. Carson

Email: dcarson@health.ucsd.edu

* Co-corresponding author: Tomoko Hayashi

Email: thayashi@ucsd.edu

Table of Contents Page

Table S1. RNA sequencing analysis of mBMDCs treated with compound **634**	S3
Table S2. Reagents used in each experiment	S4-S5
Table S3. Antibodies used in each experiment	S6
Figure S1. Intracellular Ca^{2+} screening of 80 HTS hit candidate compounds	S7
Figure S2. Intracellular Ca^{2+} mobilization of **634** plus Orai1 inhibitor	S7
Figure S3. Validation of differential gene expression using reverse transcriptase-quantitative PCR (RT-qPCR)	S8
Figure S4. Full original immunoblots presented in Figure 2C	S9
Figure S5. Flow cytometric analysis for costimulatory molecules on mBMDCs	S10
Figure S6. Full original immunoblots presented in Figure 3C	S11
Figure S7. IL-2 levels in the culture supernatants in the T cell proliferation studies in Figures 4C and E	S12
Figure S8. Measurement of **634** in an EV preparation using liquid chromatography-mass spectrometry (LC-MS)	S12
Figure S9. T cell proliferation by **634** alone or **634** plus EV_{veh}	S13
Figure S10. T cell proliferation by EVs derived from mBMDCs treated with Veh or **634** isolated with size exclusion chromatography (SEC)	S14
Figure S11. Intracellular Ca^{2+} mobilization of the SAR derivatives of **634**	S14
Figure S12. Cytotoxic effects of the SAR derivatives of **634**	S15
Figure S13. T cell proliferation in the presence of antigenic peptides by EVs derived from mBMDCs treated with the SAR derivatives of **634**	S15
Figure S14. Calculation method of area under curve (AUC) of Ca^{2+} influx	S16
Figure S15. Size distribution of diluent alone measured by MRPS	S16
Figure S16. Gating strategy for MFIs of costimulatory molecules on CD11c positive cells	S17
Figure S17. Vesicle flow cytometry gating strategy	S18
Figure S18. Gating strategy for T cell proliferation	S19
Materials and Methods: Synthesis schemes of **634** and SAR derivatives	S20-S23
Materials and Methods: ^{1}H, ^{13}C NMR, HRMS, and LC-MS for **634** and SAR derivatives	S24-S49
Materials and Methods: Biological experiments	S50
Reference	S51

Table S1. RNA sequencing analysis of mBMDCs treated with compound **634**

	Gene Symbol	Description	FC	adj.P.Val
Up-regulated	***Oit3***	oncoprotein induced transcript 3	**2.02**	2.70E-04
	Sla2	Src-like-adaptor 2	**2.04**	9.69E-03
	Htr7	5-hydroxytryptamine (serotonin) receptor 7	**2.38**	6.28E-09
	Cdh1	cadherin 1	**2.61**	4.05E-09
	Ppm1e	protein phosphatase 1E (PP2C domain containing)	**2.77**	3.35E-02
Down-regulated	***Cdhr1***	cadherin-related family member 1	**0.40**	4.42E-02
	Hrh1	histamine receptor H1	**0.46**	2.60E-05

mBMDCs were treated with compound **634** (5 μM) or Veh (0.1 % DMSO) in triplicate for 5 h. RNA was isolated, and RNA-seq analysis was performed. **634** modulated expression of 103 genes compared to Veh (FDR ≤ 0.05, and fold change > 2) (86 genes: up-regulated, 17 genes: down-regulated). Five up-regulated and two down-regulated genes related to Ca^{2+} signaling were identified by Gene Summaries from NCBI RNA reference sequence collection (RefSeq) Database, Gene Ontology (GO) Biological Process, GO Molecular Function, or KEGG pathway. Data were analyzed by UC San Diego Moore Cancer Center Biostatistics and Bioinformatics Shared Resources.

Table S2. Reagents used in each experiment

Reagents	Source	Catalog #
MPLA	Invivogen (San Diego, CA)	tlrl-mpls
PMA	Invivogen	tlrl-pma
Ionomycin	Tocris (UK)	1704
Thapsigargin	Tocris	1138
BTP2 (YM-58483)	Tocris	3939
AnCoA4	MilliporeSigma	532999
$OVA_{323-339}$ (ISQAVHAAHAEINEAGR)	AnaSpec (Fremount, CA)	AS-27024k
1 x PBS	Thermo Fisher Scientific (Waltham, MA)	14190
GM-CSF	BioLegend	576308
**Cell culture**		
RPMI 1640	Thermo Fisher Scientific	11875
Dialyzed FBS	Atlanta Biologicals (Atlanta, GA)	S12850H
Penicillin, Streptomycin	Thermo Fisher Scientific	15140122
2-mercaptoethanol	Thermo Fisher Scientific	21985
Sodium pyruvate	Thermo Fisher Scientific	11360-070
MEM non-essential amino acids	Thermo Fisher Scientific	11140-050
Blastidin	Invivogen	Ant-bl
**EV isolation**		
Exosome depleted FBS	Thermo Fisher Scientific	A27208
T182 flask	Thermo Fisher Scientific	25-211
31.5 mL open-top polypropylene UC tube	Beckman Coulter Life Sciences (Brea, CA)	358126
0.02 µm inorganic membrane filter	MilliporeSigma	6809-2002
qEVoriginal, 70 nm Gen2 Column	IZON Science (Portland, OR)	ICO-70
**T cell proliferation assay and ELISA**		
EasySep Mouse $CD4^+$ T cell isolation kit	STEMCELL Technologies (Vancouver, BC, Canada)	19852
CFSE	Thermo Fisher Scientific	C34554
Mouse IL-2 DuoSet ELISA kit	R&D systems (Minneapolis, MN)	DY402
anti-mouse DO11.10 clonotypic TCR antibody	BD Biosciences (San Diego, CA)	562524
Anti-CD80 (16-10A1)	BioXCell (Lebanon, NH)	BE0024
Anti-CD86 (GL1)	BioXCell	BE0025
American hamster IgG isotype control	BioXCell	BE0091
Rat IgG2a isotype control	R&D systems	MAB006
Stain Buffer	BD Biosciences	554657
**Calcium Influx assay**		
Fura-2-AM	Abcam	Ab120873

	(Boston, MA)	
Fura-8-AM	ATT Bioquest (Sunnyvale, CA)	21055
1 x HBSS	Corning (Union City, CA)	14175
Pluronic F127	Thermo Fisher Scientific	P3000MP
**Immunoblotting**		
PhosphoSafe extraction reagent	MilliporeSigma	71296
Protease inhibitor mixture	Roche (Manheim, Germany)	11697498001
Micro BCA Assay Kit	Thermo Fisher Scientific	23235
NuPAGE sample buffer	Thermo Fisher Scientific	NP007
NuPAGE 4-12% Bis-Tris Gels	Thermo Fisher Scientific	NP0329
Immobilon-P PVDF membranes	MilliporeSigma	IPVH00010
ProSignal Dura ECL	Thermo Fisher Scientific	34076
AccuRuler Prestained Protein Ladder	Lamda Biotech (St. Louis, MO)	26616
**CD63 Tluc-CD9 EmGFP THP-1 reporter cell assay**		
TurboLuc™ Luciferase One-Step Glow Assay Kit	Thermo Fisher Scientific	88264

Table S3. Antibodies used in this manuscript

**Antibodies (clone)**	**Dilution Factor**	**Source**	**Catalog #**
**Immunoblotting**			
Anti-CD81 (D5O2Q)	1000	Cell Signaling (Danvers, MA)	10037
Anti-Alix (3A9)	1000	Cell Signaling	2171
Anti-Calnexin (EPR3633(2))	1000	Abcam (UK)	ab133615
Anti-CD86 (E5W6H)	1000	Cell Signaling	19589S
Anti-CD80 (E6J6N)	1000	Cell Signaling	54521
Anti-MHC class II (M5/114)	1000	Abcam	ab139365
Anti-CD40 (E227J)	1000	Cell Signaling	86165
Anti-Rabbit IgG, HRP	5000	Cell Signaling	7074
Anti-mouse IgG, HRP	5000	Cell Signaling	7076
Anti-rat IgG, HRP	5000	Cell Signaling	7077
**Flow Cytometry**			
Anti-DO-11.10 clonotype TCR	300	BD Biosciences	56524
Anti-CD11c, APC (N418)	400	eBioscience	17-0114-82
Anti-CD11c, APC/Cy7 (N418)	400	BioLegend (San Diego, CA)	117324
Anti-CD86, PE (GL1)	500	eBioscience	12-0862-82
Anti-CD86, PE/Cy7 (GL1)	800	BD Biosciences	560582
Anti-CD80, APC (16-10A1)	200	BD Biosciences	560016
Anti-MHC class II [I-A], FITC (AMS-32.1)	400	BD Biosciences	553547
Anti-CD40, PE (1C10)	200	Thermo Fisher Scientific (Waltham, MA)	12-0401-83
Anti-CD16/32 (2.4G2)	300	BD Biosciences	553142
**Vesicle Flow Cytometry**			
vFRed	100	Cellarcus Biosciences	CBS4
Anti-MHC class II [I-A/I-E], Alexa Fluor 488 (M5/114)	10	BD Biosciences	562352
Anti-CD86, APC (GL1)	10	BD Biosciences	558703

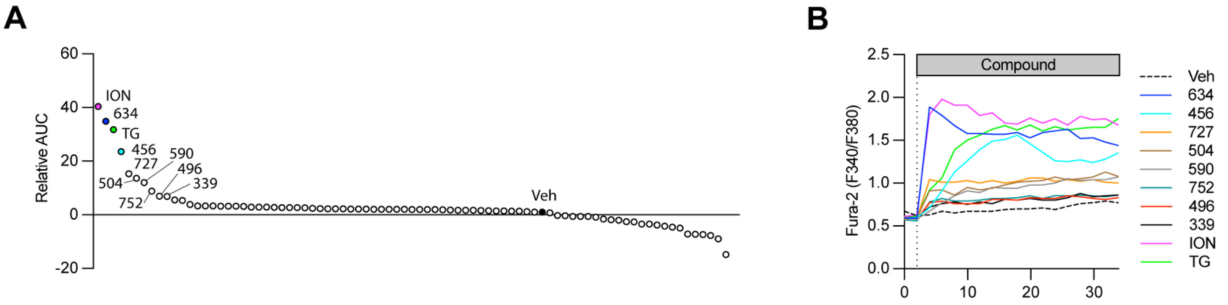

Figure S1. Intracellular Ca^{2+} screening of eighty HTS hit candidate compounds. THP-1 cells were loaded with the ratiometric Ca^{2+} indicator, Fura-2, and treated with ION (1 µM), TG (1 µM), or test compounds (5 µM). The time-response pattern of intracellular Ca^{2+} levels was recorded with a plate reader for 25 min. (A) The AUC of OD340/380 ratios corresponds to the intracellular Ca^{2+} kinetics and the baseline-subtracted AUC was calculated by GraphPad Prism (Figure S2). Data presented are relative AUC to Veh (1.74 was set as 1). (B) Ca^{2+} mobilization by the top eight compounds identified in the previously performed HTS by us [1]. Data presented are averages of duplicates and representative of two independent experiments showing similar results.

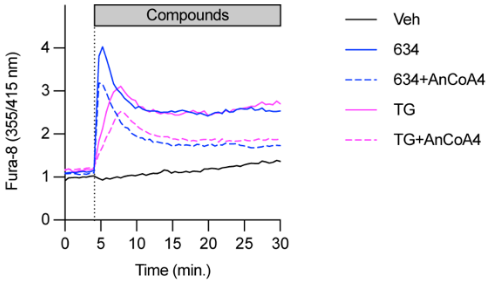

Figure S2. Intracellular Ca^{2+} mobilization of 634 plus Orai1 inhibitor. mBMDCs were loaded with the ratiometric Ca^{2+} indicator, Fura-8, and treated with Veh (0.5% DMSO), **634** alone (10 µM), **634** plus AnCoA4 (20 µM), TG (1 µM) alone or TG plus AnCoA4. The time-response pattern of intracellular Ca^{2+} levels was recorded with a plate reader for 25 min. The experiment was performed in duplicate, and the data represent two independent experiments showing similar results. The dashed line indicates the timing of compounds added.

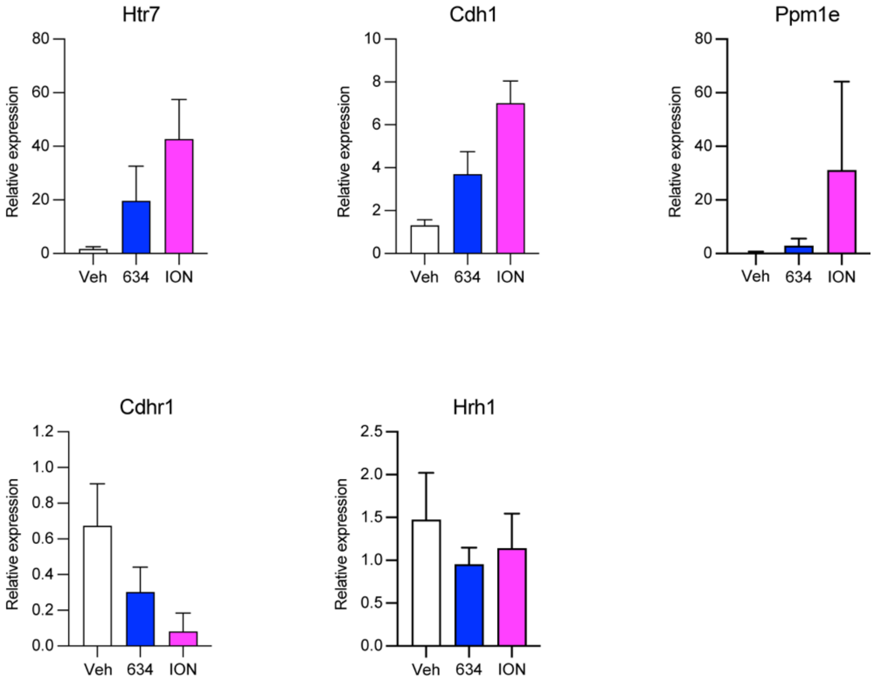

Figure S3. Validation of differential gene expression using reverse transcriptase-quantitative PCR (RT-qPCR). Relative gene expression levels of *Htr7*, *Cdh1*, *Ppm1e*, *Cdhr1* or *Hrh1* were determined by RT-qPCR. *Gapdh* was used as an internal control. mBMDCs were treated with Veh (0.1% DMSO), **634** (5 µM), or ION (1 µM) for 5 h.

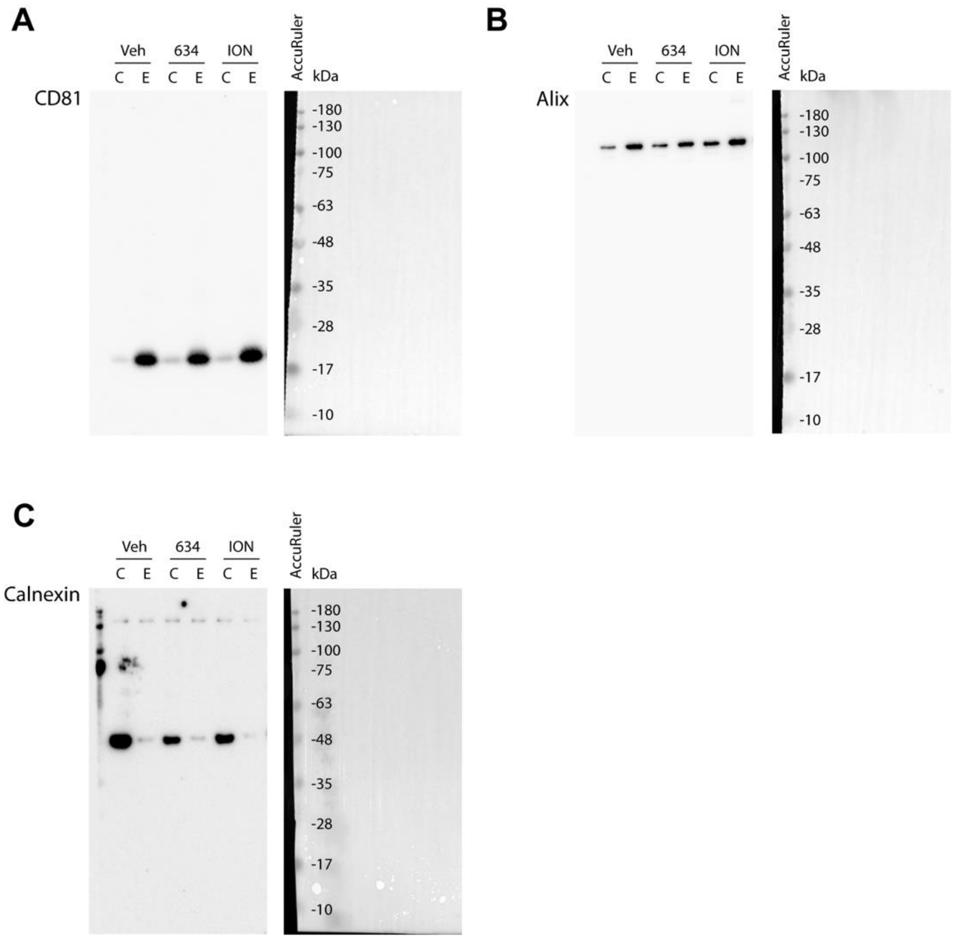

Figure S4. Full original immunoblots presented in Figure 2C. The blots of parental cell lysate (labeled as C) and EVs (labeled as E) were stained with anti-CD81(A), anti-Alix (B), and anti-Calnexin (C). In staining with CD81, samples were run under non-reducing conditions. AccuRuler Prestained Protein Ladder (Lamda Biotech) was used as a molecular weight marker.

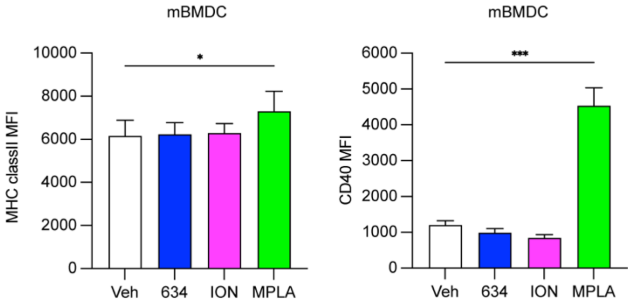

Figure S5. Flow cytometric analysis for costimulatory molecules on mBMDCs. For the analysis of co-stimulatory molecule expression on mBMDCs, mBMDCs (10^6 cells/mL) were incubated with **634** (10 µM), ION (1 µM), and MPLA (1 µg/mL) for 20-24h. 0.5% DMSO was used as a vehicle. After removing the supernatant, cells were washed with the stain buffer and incubated with anti-mouse CD16/32 antibody for blocking FcR. Cells were stained with an antibody cocktail with anti-MHC class II , and anti-CD40 antibodies for 30 min at 4 ˚C. Cells were then washed and stained with DAPI (4', 6-diamino-2phenylindole) for 10 min at room temperature to exclude dead cells (DAPI^{high}) from the analysis. Data were acquired using MACSQuant Analyzer 10 (Miltenyi Biotec, Germany) and analyzed with FlowJo (version 10.8.1). Mean fluorescence intensity (MFI) is shown. *p<0.05, ***p<0.001 by one-way ANOVA with Dunnett's *post hoc* test vs. Veh.

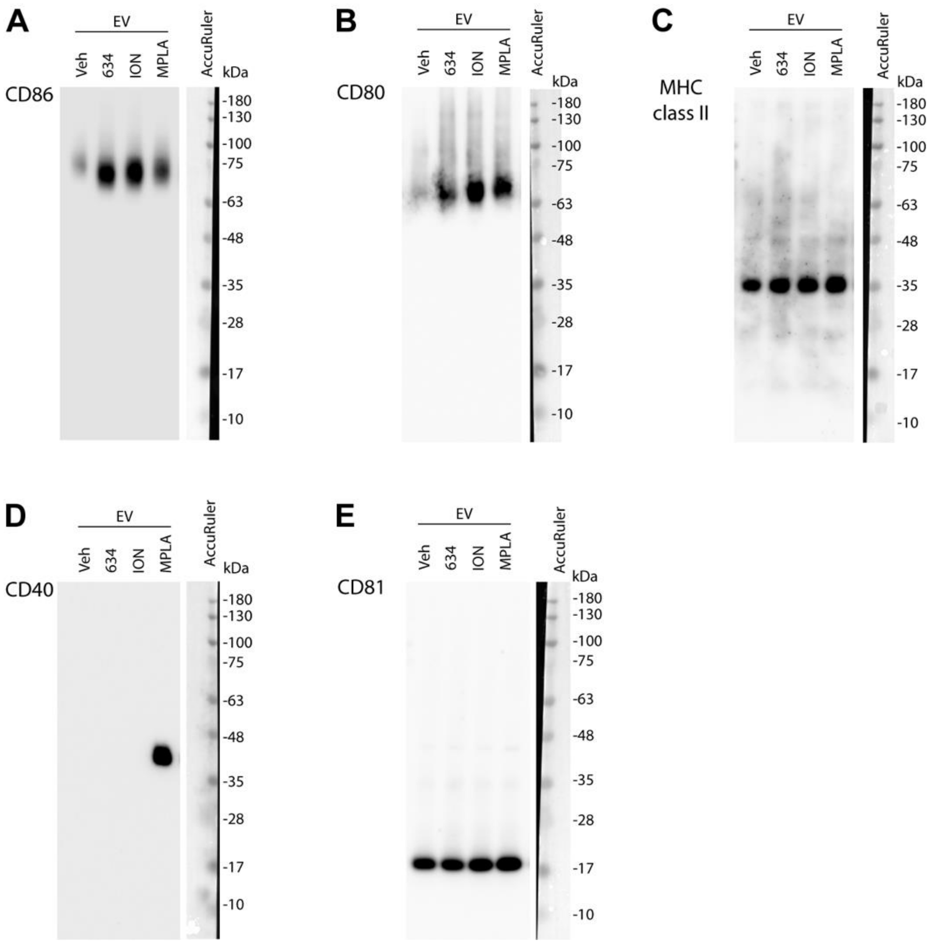

Figure S6. Full original immunoblots presented in Figure 3C. The blots of EVs were stained with anti-CD86 (A), anti-CD80 (B), anti-MHC class II (C), anti-CD40 (D)**,** and ant-CD81(E) antibodies. AccuRuler Prestained Protein Ladder (Lamda Biotech) was used as a molecular weight marker.

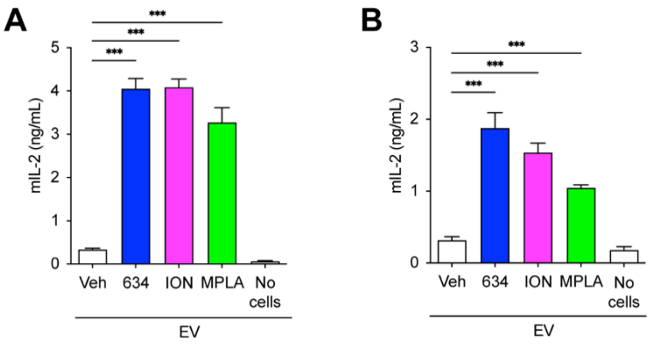

Figure S7. IL-2 levels in the culture supernatants in the T cell proliferation studies in Figures 4C and E. CFSE-labeled CD4$^+$ T cells isolated from OVA TCR transgenic strain, DO.11.10, were treated with an equal volume (7µL out of 50 µL) (A) or an equal particle number (3.13×10^9 EV particles) (B) of EV$_{\mathbf{634}}$, EV$_{ION}$, EV$_{MPLA}$, or EV$_{No\ cell}$ in the presence of OVA$_{323\text{-}339}$ peptide for three days. The supernatants were assayed for IL-2. Data shown are means ± SD of triplicates representative of two independent experiments. ***p<0.001 by one-way ANOVA with Dunnett's *post hoc* test vs. Veh.

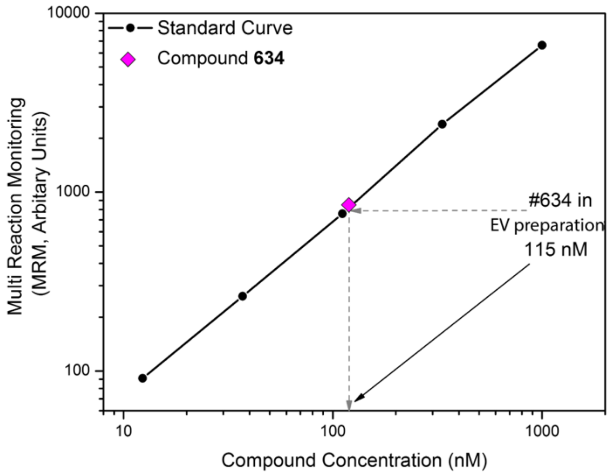

Figure S8. Measurement of 634 in an EV preparation using liquid chromatography-mass spectrometry (LC-MS). The standard curve was prepared using **634** in DMSO at 10, 37, 111, and 333 nM. The concentration of **634** was interpolated from the standard curve (115 nM in the figure below), and the concentration of **634** was corrected by dilution factor (1:4 in PBS, 460 nM). Following EV preparation, 7 µL was added to 100 µL culture medium, and the final concentration of **634** in the T cell culture was estimated at approximately 32 nM.

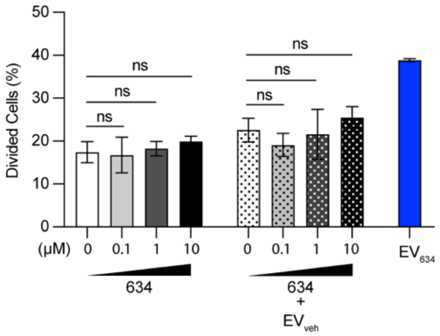

Figure S9. T cell proliferation by 634 alone or 634 plus EV_{veh}. CFSE-labeled DO11.10 TCR^+ $CD4^+$ T cells isolated were treated with Veh (0.05% DMSO), **634** alone (0.1 µM, 1 µM, or 10 µM), or **634** plus EV_{Veh} suspension (7 µL out of 50 µL), or $EV_{\mathbf{634}}$ suspension (7 µL out of 50 µL) alone in the presence of $OVA_{323-339}$ peptide for 5 days. T cell proliferation was determined by CFSE dilution using flow cytometry. Percentages of divided T cells relative to the original population are calculated. Data shown are means ± SD of triplicates representative of two independent experiments. n.s., not significant by two-way ANOVA with Tukey's *post hoc* test vs. Veh.

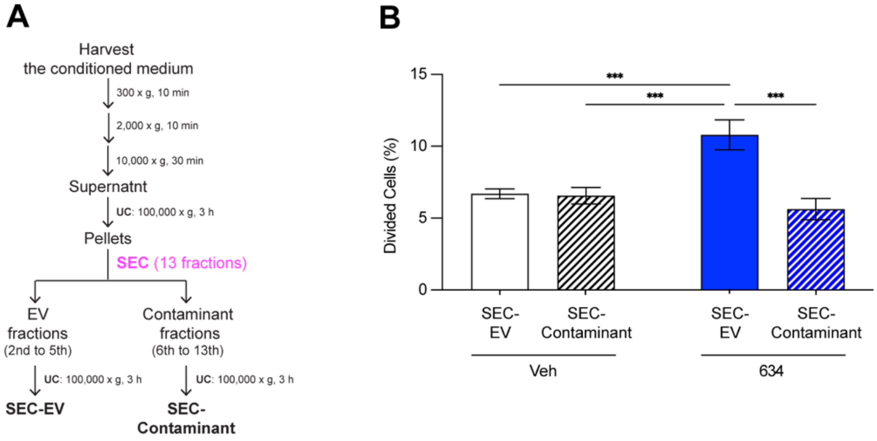

Figure S10. T cell proliferation by EVs derived from mBMDCs treated with Veh or 634 isolated with size exclusion chromatography (SEC). (A) mBMDCs were incubated with Veh (0.5 %) or **634** (10 µM) for 48 h. Following EVs were isolated by UC, the pellets were suspended in 500 µL PBS, and then EV suspension was loaded on SEC (IZON qEV columns). Thirteen fractions were collected (0.4 ml of each) after the first 2.1 mL flow-through. Four fractions (from 2nd fraction to 5th fraction) were collected as SEC-EV, and following 7 fractions (from 6th fraction to 13th fraction) were collected as SEC-Contaminant. SEC-EV and SEC-Contaminant were concentrated by ultracentrifugation and resuspended with 50 µL PBS (designated as SEC-EV$_{veh}$, SEC-EV$_{\mathbf{634}}$, SEC-Contaminant$_{Veh}$, and SEC-Contaminant$_{\mathbf{634}}$). (B) CFSE-labeled DO11.10 TCR^{+} CD4^{+} T cells were treated with an equal volume of SEC-EV$_{veh}$, SEC-EV$_{\mathbf{634}}$, SEC-Contaminant$_{Veh}$, and SEC-Contaminant$_{634}$ suspension (7 µL out of 50 µL) in the presence of OVA$_{323\text{-}339}$ peptide for 5 days. T cell proliferation was determined by CFSE dilution using flow cytometry. Percentages of divided T cells relative to the original population are calculated. Data shown are means ± SD of triplicates. ****p*<0.001 by two-way ANOVA with Tukey's *post hoc* test.

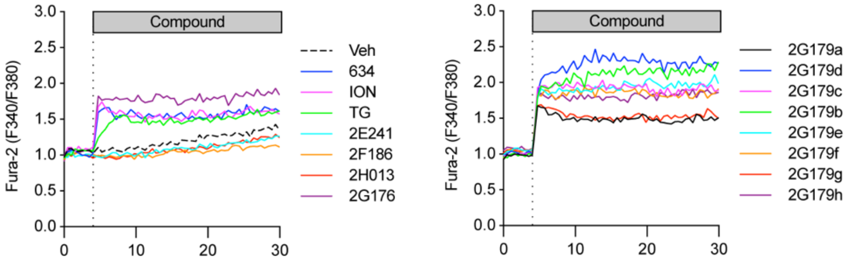

Figure S11. Intracellular Ca^{2+} mobilization of the SAR derivatives of 634. mBMDCs were loaded with the ratiometric Ca^{2+} indicator, Fura-2, and treated with Veh (0.5% DMSO), ION (1 µM), TG (1 µM), or test compounds (10 µM). The time-response pattern of intracellular Ca^{2+} levels was recorded with a plate reader for 25 min. The experiment was performed in duplicate, and the data represent two independent experiments showing similar results. The dashed line indicates the timing of compounds added.

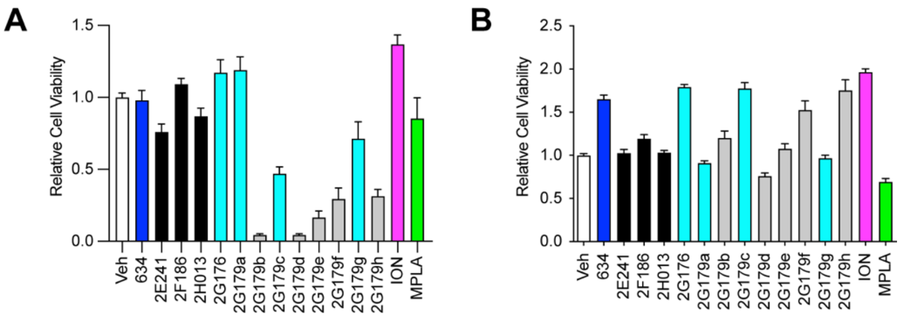

Figure S12. Cytotoxic effects of the SAR derivatives of 634. mBMDCs were cultured in RPMI1640 supplemented with 10% exosome depleted FBS (A) or with 10% fetal bovine serum (FBS) (B) and treated with Veh (0.5% DMSO), Ionomycin (ION, 1 µM), MPLA (1 µg/mL), or test compounds (10 µM). After 48 h incubation, cell viability was determined by MTT assay. The relative viability was normalized to Veh-treated cells [Veh was 1.00 ± 0.03 at (A), and 1.00 ± 0.02 at (B)]. Data shown are means ± SD of triplicates of a representative experiment of two independent experiments.

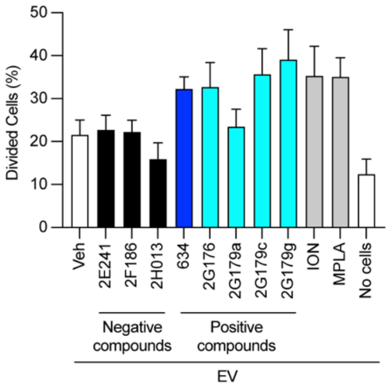

Figure S13. T cell proliferation in the presence of antigenic peptides by EVs derived from mBMDCs treated with the SAR derivatives of 634. CFSE-labeled DO11.10 TCR$^+$ CD4$^+$ T cells were treated with an equal volume of EV suspension (7µL out of 50 µL) in the presence of OVA$_{323-339}$ peptide for 5 days. T cell proliferation was determined by CFSE dilution using flow cytometry. Percentages of divided T cells relative to the original population are calculated. Data shown are means ± SEM of three independent experiments performed in triplicates.

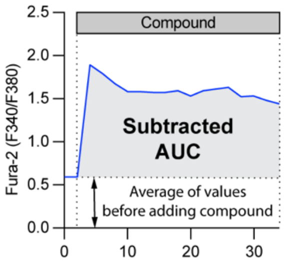

Figure S14. Calculation method of Area Under Curve (AUC) of Ca^{2+} influx. The baseline-subtracted AUC of 340/380 ratios was calculated as net AUC using GraphPad Prism. The baseline was calculated as the mean of 340/380 ratios without compound in each measurement.

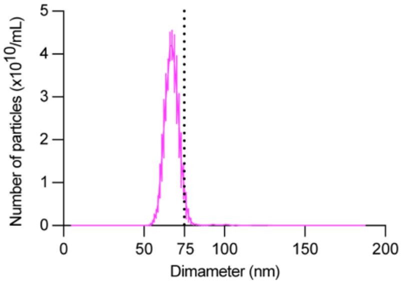

Figure S15. Size distribution of diluent alone measured by MRPS. Size distribution of particles in the dilutant (1% Tween 20 in PBS) was measured by MRPS. Data shown are means ± SEM of data from 3 measurements.

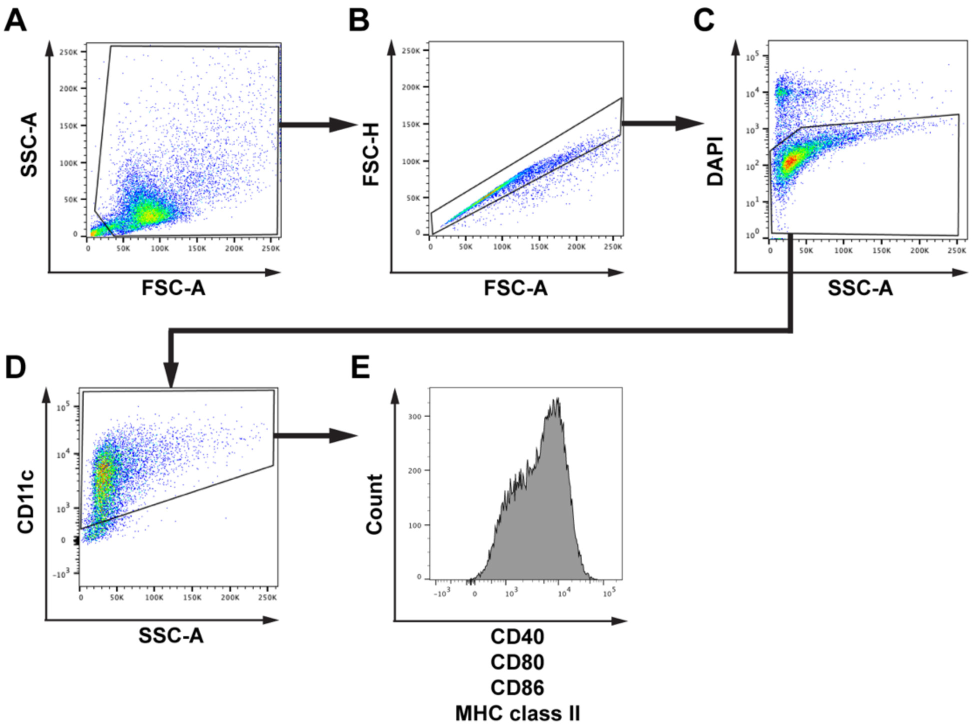

Figure S16. Gating strategy of costimulatory molecules expression on CD11c positive cells. Flow cytometry data were gated to distinguish (A) lymphocytes and (B) singlets based on forward and side scatter. (C) DAPI^{high} dead cells were excluded from the analysis. (D) CD11c positive cells were gated, and MFIs of costimulatory molecules on CD11c positive cells were calculated.

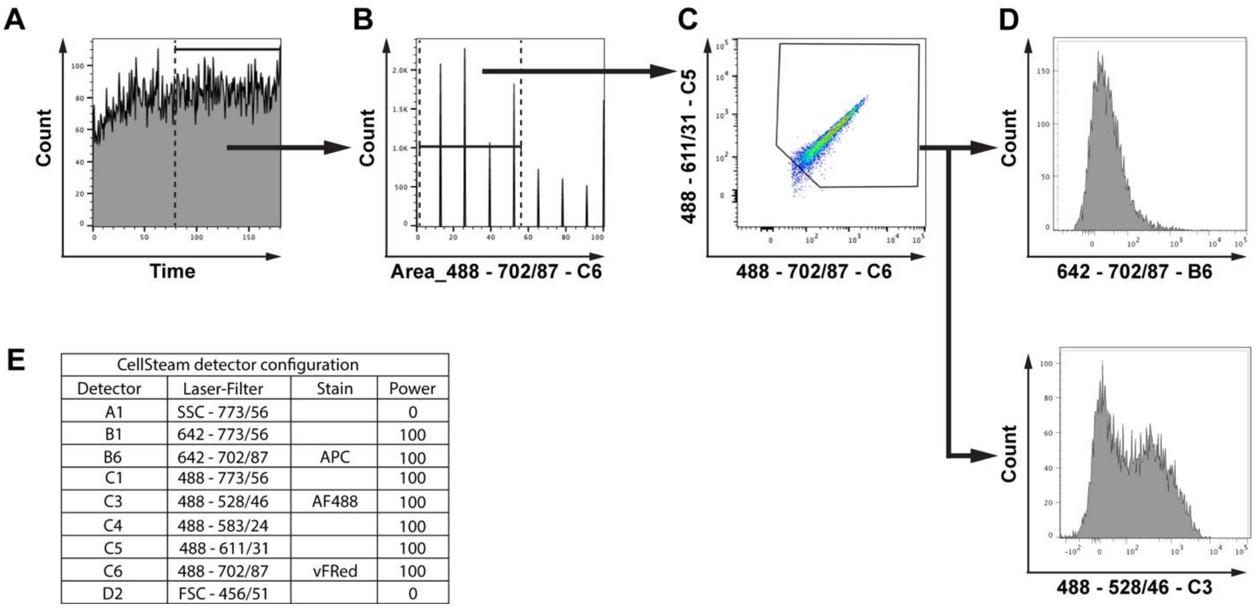

CellSteam detector configuration			
Detector	Laser-Filter	Stain	Power
A1	SSC - 773/56		0
B1	642 - 773/56		100
B6	642 - 702/87	APC	100
C1	488 - 773/56		100
C3	488 - 528/46	AF488	100
C4	488 - 583/24		100
C5	488 - 611/31		100
C6	488 - 702/87	vFRed	100
D2	FSC - 456/51		0

Figure S17. Vesicle flow cytometry gating strategy. (A) Data were gated on time to remove events associated with any fluidic anomalies. (B) The particles larger than four pixels on the vFRed™ object area were excluded because they would be coincident or out-of-focus events. (C) These events were further gated to exclude low-intensity background events. (D) MFI of CD86 and MHC class II on vesicles was calculated. (E) The CellStream flow cytometer configuration was used in this study. FSC and SSC lasers were turned off, and its Small Particle mode was activated. Each sample was introduced at the Slow sample flow rate (3.66 µL/min) and analyzed for 20 seconds.

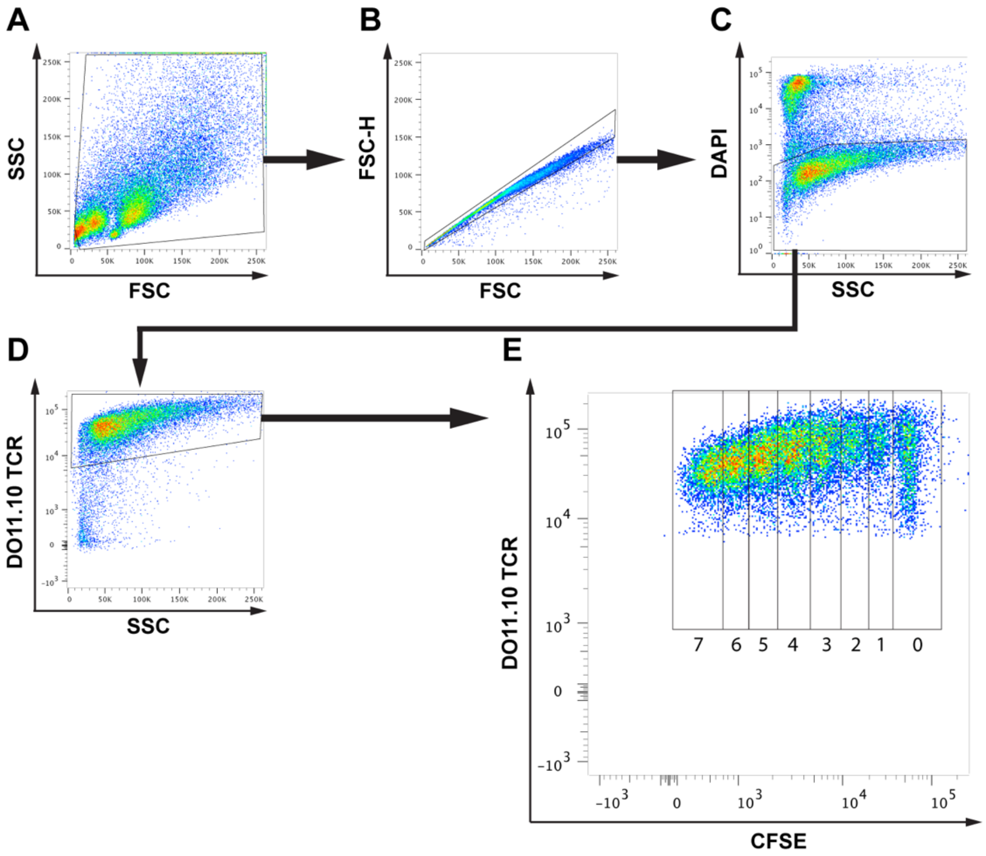

Figure S18. Gating strategy for T cell proliferation. Flow cytometry data were gated to distinguish (A) lymphocytes and (B) singlets based on forward and side scatter. (C) DAPI^{high} dead cells were excluded from the analysis. (D) DO11.10 TCR-positive cells were gated. Cell proliferation was monitored by CFSE dilution in DO11.10 TCR gated population. Percentages of divided cells relative to the original population were calculated.

Materials and Methods: Chemical Synthesis
Chemical reagents were purchased as at least reagent grade from commercial vendors unless otherwise specified and used without further purification. Solvents were purchased from Fischer Scientific (Pittsburgh, PA) and were either used as purchased or redistilled with an appropriate drying agent.

Instrumentation. Analytical TLC was performed using precoated TLC silica gel 60 F_{254} aluminum sheets purchased from EMD (Gibbstown, NJ) and visualized using UV light. Flash chromatography was carried out using a Biotage Isolera One (Charlotte, NC) system for normal phase column chromatography or Teledyne ISCO ACCQPrep HP150 for C18-reverse phase column chromatography using the specified solvent. Reaction monitoring and purity analysis were done using an Agilent 1260 LC/6420 Triple Quad mass spectrometer (Santa Clara, CA) with Onyx Monolithic C18 (Phenomenex, Torrance, CA) column. Purity of all final compounds was above 95% (also see LC-MS spectra in Supporting Information for all final compounds). All final compounds were analyzed by high resolution MS (HRMS) using an Agilent 6230 ESI-TOFMS (Santa Clara, CA). ^{1}H and ^{13}C NMR spectra were obtained on a Varian 500 with XSens probe (Varian, Inc., Palo Alto, CA). The chemical shifts are expressed in parts per million (ppm) using suitable deuterated NMR solvents.

Experimental Section
Ethyl 2-(benzo[*c*][1,2,5]thiadiazole-4-sulfonamido)-4,5-dimethylthiophene-3-carboxylate (**634**). In a round bottom, benzo[*c*][1,2,5]thiadiazole-4-sulfonyl chloride (400 mg, 1.71 mmol) and ethyl 2-amino-4,5-dimethylthiophene-3-carboxylate (340 mg, 5.17 mmol) were dissolved in anhydrous CH_2Cl_2 (1 mL), followed by the addition of pyridine (413 µL, 1.29 mmol) to the reaction mixture and stirred overnight. Solvent was then removed, and the residue was recrystallized in isopropanol to yield 302 mg of **634** (44.5% yield). ^{1}H NMR (500 MHz, CHLOROFORM-d) d 11.10 (s, 1H), 8.34 (d, *J* = 7.09 Hz, 1H), 8.24 (d, *J* = 8.56 Hz, 1H), 7.71 (t, *J* = 7.80 Hz, 1H), 4.28 (q, *J* = 7.09 Hz, 2H), 2.16 (s, 3H), 2.08 (s, 3H), 1.35 (t, *J* = 7.21 Hz, 3H). ^{13}C NMR (126 MHz, CHLOROFORM-d) d 165.1, 155.2, 148.8, 145.5, 132.2, 130.3, 130.0, 127.9, 127.1, 123.0, 114.5, 60.8, 29.7, 14.2, 12.4. HRMS for $C_{15}H_{15}N_3O_4S_3$ [M + Na^+] calculated 420.0117, found 420.0118.

2-(benzo[*c*][1,2,5]thiadiazole-4-sulfonamido)-4,5-dimethylthiophene-3-carboxylic acid (**2H013**). In a reaction vial, compound **634** (300 mg, 0.756 mmol) was dissolved in a 1:1 ratio of methanol (1mL) and tetrahydrofuran (1 mL). In a separate vial, lithium hydroxide (47.4 mg, 1.129 mmol) was dissolved in water (0.4 mL, 20% of the organic volume) and added to the reaction mixture. The reaction was heated to 45℃ and allowed to stir overnight. Solvent was then removed and extracted with ethyl acetate, water, and hydrochloric acid. The organic layer was collected, and the residue was purified by HPLC prep system to yield 218 mg of compound **2H013** (78.4 % yield). ^{1}H NMR (500 MHz, CHLOROFORM-d) d 11.00 (s, 1H), 8.29 (d, *J* = 6.85 Hz, 1H), 8.20 (d, *J* = 8.56 Hz, 1H), 7.66 (t, *J* = 7.80 Hz, 1H), 2.14 (s, 6H). ^{13}C NMR (126 MHz, CHLOROFORM-d) d 169.9, 155.2, 148.7, 148.3, 132.1, 130.8, 129.8, 127.9, 127.4, 123.3, 113.1, 14.4, 12.5. HRMS for $C_{13}H_{11}N_3O_4S_3Na$ $[M + Na]^+$ calculated 391.9804, found 391.9802.

2-(benzo[*c*][1,2,5]thiadiazole-4-sulfonamido)-4,5-dimethylthiophene-3-carbonyl chloride (**2F230**). Compound **2H013** (15 mg, mmol) was dissolved in anhydrous CH_2Cl_2 (0.5mL). Thionyl chloride (0.5mL) was then added and stirred at room temperature for 30 min. A 1:1 volume ratio of CH_2Cl_2 and thionyl chloride is used. Solvent was then removed and washed with CH_2Cl_2 multiple times. The residue was not purified and used an intermediate. 100% yield is assumed to yield 15.7 mg of **2F230**.

Propyl 2-(benzo[*c*][1,2,5]thiadiazole-4-sulfonamido)-4,5-dimethylthiophene-3-carboxylate (**2G176**). In a reaction vial N-propanol (0.3 mL) and triethylamine (TEA) (6 µL, 0.043mmol) were mixed and added to compound **2F230** (8.4 mg, 0.022 mmol). The reaction mixture was stirred at room temperature until completion and purified by HPLC prep to yield 2.4 mg of compound **2G176** (28.6% yield). ^{1}H NMR (500 MHz, CHLOROFORM-d) d 11.15 (s, 1H), 8.34 (d, *J* = 7.09 Hz, 1H), 8.16 - 8.28 (m, 1H), 7.70 (dd, *J* = 7.09, 8.80 Hz, 1H), 4.18 (t, *J* = 6.60 Hz, 2H), 2.16 (s, 3H), 2.08 (s, 3H), 1.69 - 1.79 (m, 2H), 0.98 (t, *J* = 7.46 Hz, 3H). ^{13}C NMR (126 MHz, CHLOROFORM-d) d 165.3, 155.2, 148.9, 145.6, 132.2, 130.3, 130.0, 127.9, 127.1, 122.9, 114.5, 66.5, 21.9, 14.3, 12.4, 10.7. HRMS for $C_{16}H_{17}N_3O_4S_3Na$ $[M + Na]^+$ calculated 434.0273, found 434.0271.

Methyl 2-(benzo[*c*][1,2,5]thiadiazole-4-sulfonamido)-4,5-dimethylthiophene-3-carboxylate (**2G179a**). In a reaction vial, anhydrous methanol in excess (0.5mL) and TEA (11.3 µL. 0.081 mmol) were mixed and added to compound **2F230** (15.7 mg, 0.041 mmol). The reaction mixture was stirred at 40°C until completion. Solvent was then removed, and the residue was purified by HPLC prep system to yield 11.5 mg of **2G179a** (74.2% yield). ^{1}H NMR (500 MHz, CHLOROFORM-d) d 10.99 (s, 1H), 8.34 (d, *J* = 7.00 Hz, 1H), 8.24 (d, *J* = 8.56 Hz, 1H), 7.71 (t, *J* = 8.80 Hz, 1H), 3.82 (s, 3H), 2.16 (s, 3H), 2.07 (s, 3H). ^{13}C NMR (126 MHz, CHLOROFORM-d) d 165.4, 155.2, 148.8, 145.5, 132.2, 130.3, 130.0, 127.9, 127.1, 123.1, 114.4, 51.7, 14.2, 12.4. HRMS for $C_{14}H_{13}N_3O_4S_3Na$ $[M + Na]^+$ calculated 405.9960, found 405.9961.

Hexyl 2-(benzo[*c*][1,2,5]thiadiazole-4-sulfonamido)-4,5-dimethylthiophene-3-carboxylate (**2G179b**). Compound **2F230** (15.7 mg, 0.041 mmol), TEA (11.3 µL, 0.081 mmol), and N-hexanol in excess (0.5mL) were reacted according to the general procedure of compound **2G179a** to yield 5.9 mg of compound **2G179b** (32.1% yield). ^{1}H NMR (500 MHz, CHLOROFORM-d) d 11.16 (s, 1H), 8.35 (d, *J* = 7.09 Hz, 1H), 8.24 (d, *J* = 8.80 Hz, 1H), 7.71 (t, *J* = 7.80 Hz, 1H), 4.22 (t, *J* = 6.72 Hz, 2H), 2.16 (s, 3H), 2.08 (s, 3H), 1.70 (quin, *J* = 7.09 Hz, 2H), 1.36 - 1.42 (m, 2H), 1.30 - 1.35 (m, 4H), 0.90 (t, *J* = 6.72 Hz, 3H). ^{13}C NMR (126 MHz, CHLOROFORM-d) d 165.3, 155.2, 148.9, 145.6, 132.2, 130.3, 130.0, 127.9, 127.1, 122.9, 114.5, 65.0, 31.4, 28.5, 25.7, 22.5, 14.3, 14.0, 12.4. HRMS for $C_{19}H_{23}N_3O_4S_3Na$ $[M + Na]^+$ calculated 476.0743, found 476.0741.

Isopropyl 2-(benzo[*c*][1,2,5]thiadiazole-4-sulfonamido)-4,5-dimethylthiophene-3-carboxylate (**2G179c**). Compound **2F230** (15.7 mg, 0.041 mmol), TEA (11.3 µL, 0.081 mmol), and 2-propanol in excess (0.5mL) were reacted according to the general procedure of compound **2G179a** to yield 7.6 mg of compound **2G179c** (45.5% yield). ^{1}H

NMR (500 MHz, CHLOROFORM-d) d 11.20 (s, 1H), 8.29 - 8.39 (m, 1H), 8.18 - 8.28 (m, 1H), 7.70 (dd, *J* = 7.09, 8.80 Hz, 1H), 5.10 - 5.21 (m, 1H), 2.15 (s, 3H), 2.06 (s, 3H), 1.30 (d, *J* = 6.36 Hz, 6H). ^{13}C NMR (126 MHz, CHLOROFORM-d) d 164.8, 155.2, 148.9, 145.4, 132.2, 130.3, 130.0, 127.9, 127.1, 122.9, 114.7, 68.6, 21.9, 14.3, 12.4. HRMS for $C_{16}H_{17}N_3O_4S_3Na$ [M + Na]$^+$ calculated 434.0273, found 434.0269.

Isopentyl 2-(benzo[*c*][1,2,5]thiadiazole-4-sulfonamido)-4,5-dimethylthiophene-3-carboxylate (**2G179d**). Compound **2F230** (15.7 mg, 0.041 mmol), TEA (11.3 µL, 0.081 mmol), and iso amyl alcohol in excess (0.5mL) were reacted according to the general procedure of compound **2G179a** to yield 4.9 mg of compound **2G179d** (27.5% yield). ^{1}H NMR (500 MHz, CHLOROFORM-d) d 11.14 (s, 1H), 8.34 (d, *J* = 7.09 Hz, 1H), 8.20 - 8.29 (m, 1H), 7.70 (dd, *J* = 7.09, 8.80 Hz, 1H), 4.25 (t, *J* = 6.85 Hz, 2H), 2.15 (s, 3H), 2.07 (s, 3H), 1.68 - 1.76 (m, 1H), 1.59 (q, *J* = 6.93 Hz, 2H), 0.95 (s, 3H), 0.93 (s, 3H). ^{13}C NMR (126 MHz, CHLOROFORM-d) d 165.3, 155.2, 152.8, 148.9, 145.6, 132.2, 130.3, 127.9, 127.1, 122.9, 114.4, 63.4, 37.2, 25.1, 22.4, 14.3, 12.4. HRMS for $C_{18}H_{21}N_3O_4S_3Na$ [M + Na]$^+$ calculated 462.0586, found 462.0582.

Cyclohexyl 2-(benzo[*c*][1,2,5]thiadiazole-4-sulfonamido)-4,5-dimethylthiophene-3-carboxylate (**2G179e**). Compound **2F230** (15.7 mg, 0.041 mmol), TEA (11.3 µL, 0.081 mmol), and cyclohexanol in excess (0.5mL) were reacted according to the general procedure of compound **2G179a** to yield 5.6 mg of compound **2G179e** (30.6% yield). ^{1}H NMR (500 MHz, CHLOROFORM-d) d 11.22 (s, 1H), 8.30 - 8.40 (m, 1H), 8.18 - 8.28 (m, 1H), 7.71 (dd, *J* = 7.09, 8.80 Hz, 1H), 4.96 (quin, *J* = 4.20 Hz, 1H), 2.16 (s, 3H), 2.09 (s, 3H), 1.84 - 1.93 (m, 2H), 1.71 - 1.77 (m, 2H), 1.37 - 1.60 (m, 6H). ^{13}C NMR (126 MHz, CHLOROFORM-d) d 164.7, 155.2, 148.9, 145.5, 132.2, 130.3, 130.0, 127.9, 127.1, 122.8, 114.8, 73.3, 31.5, 29.7, 25.3, 23.5, 14.5, 12.4. HRMS for $C_{19}H_{21}N_3O_4S_3Na$ [M + Na]$^+$ calculated 474.0586, found 474.0582.

Benzyl 2-(benzo[*c*][1,2,5]thiadiazole-4-sulfonamido)-4,5-dimethylthiophene-3-carboxylate (**2G179f**). Compound **2F230** (15.7 mg, 0.041 mmol), TEA (11.3 µL, 0.081 mmol), and benzyl alcohol in excess (0.5mL) were reacted according to the general procedure of compound **2G179a** to yield 3.2 mg of compound **2G179f** (17.2% yield). ^{1}H NMR (500 MHz, CHLOROFORM-d) d 11.13 (s, 1H), 8.28 - 8.37 (m, 1H), 8.21 (d, *J* = 8.80 Hz, 1H), 7.69 (dd, *J* = 7.09, 8.56 Hz, 1H), 7.33 - 7.42 (m, 5H), 5.27 (s, 2H), 2.14 (s, 3H), 2.06 (s, 3H). ^{13}C NMR (126 MHz, CHLOROFORM-d) d 164.9, 155.2, 148.7, 146.1, 135.5, 132.2, 130.3, 129.9, 128.7, 128.4, 128.4, 127.9, 127.1, 123.0, 114.1, 66.5, 14.4, 12.4. HRMS for $C_{20}H_{17}N_3O_4S_3Na$ [M + Na]$^+$ calculated 482.0273, found 482.0267.

Phenyl 2-(benzo[*c*][1,2,5]thiadiazole-4-sulfonamido)-4,5-dimethylthiophene-3-carboxylate (**2G179g**). In a reaction vial, phenol (36.5 mg, 0.88 mmol) and TEA (6 µL, 0.043mmol) were dissolved in CH_2Cl_2 (0.5mL) and was added to compound **2F230** (15.7 mg, 0.022 mmol). The reaction mixture was stirred at 40°C until completion. Solvent was removed and the residue was purified by HPLC prep system to yield 5.2 mg of **2G179g** (28.8 % yield). ^{1}H NMR (500 MHz, CHLOROFORM-d) d 10.92 (s, 1H), 8.36 (d, *J* = 6.36 Hz, 1H), 8.20 - 8.30 (m, 1H), 7.72 (dd, *J* = 7.09, 8.80 Hz, 1H), 7.45 (t, *J* = 7.83 Hz, 2H), 7.31 (t, *J* = 7.40 Hz, 1H), 7.06 (d, *J* = 7.58 Hz, 2H), 2.21 (s, 6H). ^{13}C NMR

(126 MHz, CHLOROFORM-d) d 163.6, 155.2, 149.8, 148.8, 147.3, 132.2, 130.3, 129.9, 129.6, 127.9, 127.2, 126.2, 123.3, 121.6, 113.5, 14.4, 12.5. HRMS for $C_{19}H_{15}N_3O_4S_3Na$ $[M + Na]^+$ calculated 468.0117, found 468.0114.

Tert-butyl 2-(benzo[*c*][1,2,5]thiadiazole-4-sulfonamido)-4,5-dimethylthiophene-3-carboxylate (**2G179h**). Compound **2F230** (15.7 mg, 0.041 mmol), TEA (11.3 µL, 0.081 mmol), and tert butanol in excess (0.5mL) were reacted according to the general procedure of compound **2G179a** to yield 5.4 mg of compound **2G179h** (31.2% yield). ^{1}H NMR (500 MHz, CHLOROFORM-d) d 11.28 (s, 1H), 8.28 - 8.38 (m, 1H), 8.18 - 8.28 (m, 1H), 7.70 (dd, *J* = 7.09, 8.80 Hz, 1H), 2.14 (s, 3H), 2.04 (s, 3H), 1.52 (s, 9H). ^{13}C NMR (126 MHz, CHLOROFORM-d) d 164.5, 155.2, 148.9, 144.9, 132.3, 130.3, 130.0, 128.0, 127.0, 122.8, 115.8, 82.2, 28.2, 14.4, 12.4. HRMS for $C_{17}H_{19}N_3O_4S_3Na$ $[M + Na]^+$ calculated 448.0430, found 448.0434.

Ethyl 4,5-dimethyl-2-(N-methylbenzo[*c*][1,2,5]thiadiazole-4-sulfonamido)thiophene-3-carboxylate (**2F186**). Compound **2E213** (10 mg, 0.025 mmol) and potassium carbonate (6.7 mg, 0.050mmol) was dissolved in anhydrous dimethylformamide. Iodomethane (1.71 µL, 0.028 mmol) was added to the reaction mixture and stirred at 45℃ until completion. The reaction mixture was extracted with ethyl acetate, water and hydrochloric acid. Solvent was removed, and the residue was purified by HPLC prep to yield 5.7 mg of **2F186** (54.8 % yield)**.** ^{1}H NMR (500 MHz, CHLOROFORM-d) d 8.25 (d, *J* = 8.80 Hz, 1H), 8.13 (d, *J* = 7.00 Hz, 1H), 7.66 (t, *J* = 8.60 Hz, 1H), 3.97 (q, *J* = 7.09 Hz, 2H), 3.64 (s, 3H), 2.21 (s, 3H), 2.16 (s, 3H), 1.26 (t, *J* = 7.21 Hz, 3H)**.** ^{13}C NMR (126 MHz, CHLOROFORM-d) d 163.1, 155.5, 149.7, 140.8, 132.4, 132.4, 131.7, 131.3, 128.2, 126.5, 60.7, 41.9, 14.0, 13.3, 13.3**.** HRMS for $C_{16}H_{18}N_3O_4S_3$ $[M + H]^+$ calculated 412.0454, found 412.0457.

2-(benzo[*c*][1,2,5]thiadiazole-4-sulfonamido)-N-ethyl-4,5-dimethylthiophene-3-carboxamide (**2E241**). Compound **2H013** (10 mg, 0.027 mmol) was dissolved in anhydrous CH_2Cl_2 and ethylamine in THF (2M) (40 µL, 0.081 mmol) was added to the reaction mixture. Solvent was removed, and the residue was purified by HPLC prep to yield 3 mg of **2E241 (**28.0% yield)**.** ^{1}H NMR (500 MHz, CHLOROFORM-d) d 10.01 (br. s., 1H), 8.26 (d, *J* = 4.16 Hz, 1H), 8.25 (d, *J* = 2.20 Hz, 1H), 7.70 (t, *J* = 7.80 Hz, 1H), 6.22 (br. s., 1H), 3.39 (quin, *J* = 6.80 Hz, 2H), 2.12 (s, 3H), 2.08 (s, 3H), 1.20 (t, *J* = 7.21 Hz, 3H)**.** ^{13}C NMR (126 MHz, CHLOROFORM-d) d 164.8, 155.2, 149.0, 136.7, 132.0, 130.0, 128.3, 128.1, 127.0, 126.2, 34.7, 14.7, 13.5, 12.8. HRMS for $C_{15}H_{17}N_4O_3S_3$ calculated $[M + H]^+$ calculated 397.0457, found 397.0457.

^{1}H, ^{13}C NMR, HRMS, and LC-MS for all compounds

^{1}H, ^{13}C NMR **634**

Formula	$C_{15}H_{15}N_3O_4S_3$	FW	397.4923

Acquisition Time (sec)	1.9975	Date	16 Aug 2022 07:50:44			Date Stamp	16 Aug 2022 07:49:30
File Name	C:\Users\Mycoahhh\Documents\NMR\michan\634\2e213_PROTON-1-1.jdf					Frequency (MHz)	399.91
Nucleus	1H	Number of Transients	16	Origin	ECZ 402	Original Points Count	14998
Owner	michan	Points Count	16384	Pulse Sequence	single_pulse.jxp	Solvent	CHLOROFORM-d
Spectrum Offset (Hz)	2008.3990	Spectrum Type	STANDARD	Sweep Width (Hz)	7508.51	Temperature (degree C)	22.200

^{1}H NMR (400 MHz, CHLOROFORM-d) d 11.09 (s, 1H), 8.34 (d, J = 6.87 Hz, 1H), 8.23 (d, J = 9.17 Hz, 1H), 7.70 (t, J = 8.20 Hz, 1H), 4.28 (q, J = 6.87 Hz, 2H), 2.16 (s, 3H), 2.08 (s, 3H), 1.35 (t, J = 7.33 Hz, 3H)

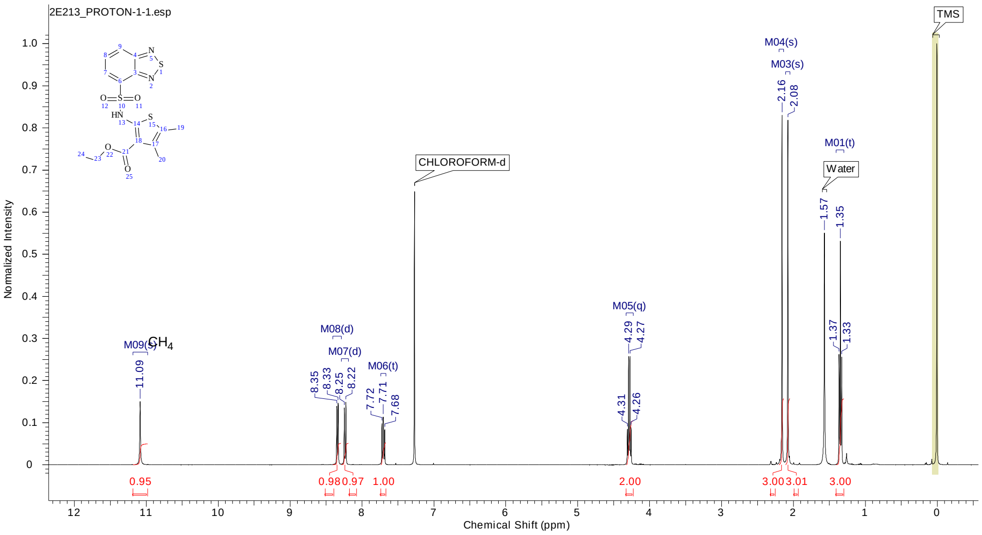

Formula	$C_{15}H_{15}N_3O_4S_3$	FW	397.4923

Acquisition Time (sec)	1.3005	Comment	Std carbon	Date	Dec 14 2020	Date Stamp	Dec 14 2020
File Name	C:\Users\Mycoahhh\Documents\NMR\michan\634\2E213-carbon.fid\fid					Frequency (MHz)	125.69
Nucleus	13C	Number of Transients	80	Original Points Count	39649	Points Count	65536
Pulse Sequence	s2pul	Receiver Gain	30.00	Solvent	CHLOROFORM-d		
Spectrum Offset (Hz)	13191.9609	Spectrum Type	STANDARD	Sweep Width (Hz)	30487.80	Temperature (degree C)	30.000

^{13}C NMR (126 MHz, CHLOROFORM-d) d 165.1, 155.2, 148.8, 145.5, 132.2, 130.3, 130.0, 127.9, 127.1, 123.0, 114.5, 60.8, 29.7, 14.2, 12.4

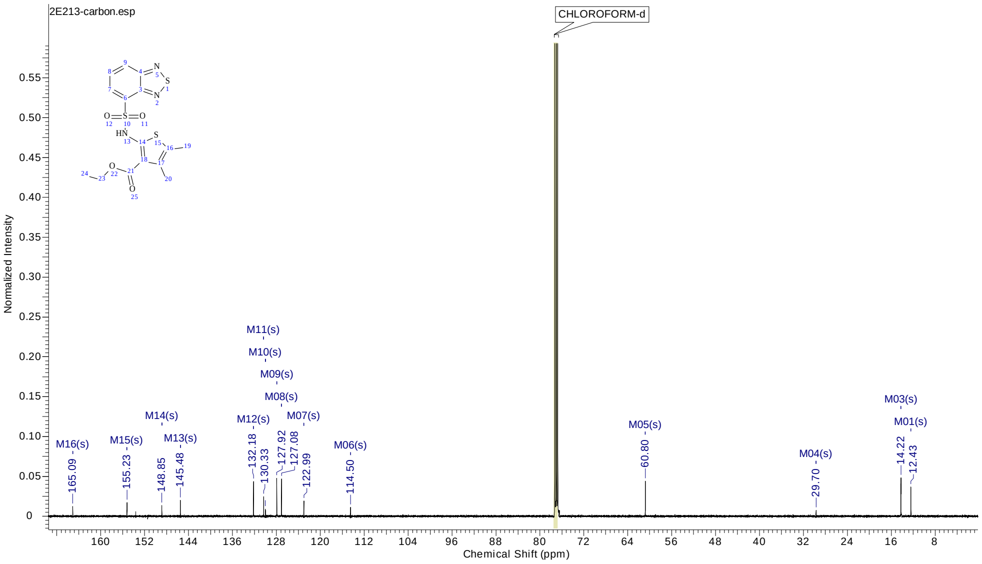

HRMS and LC-MS **634**

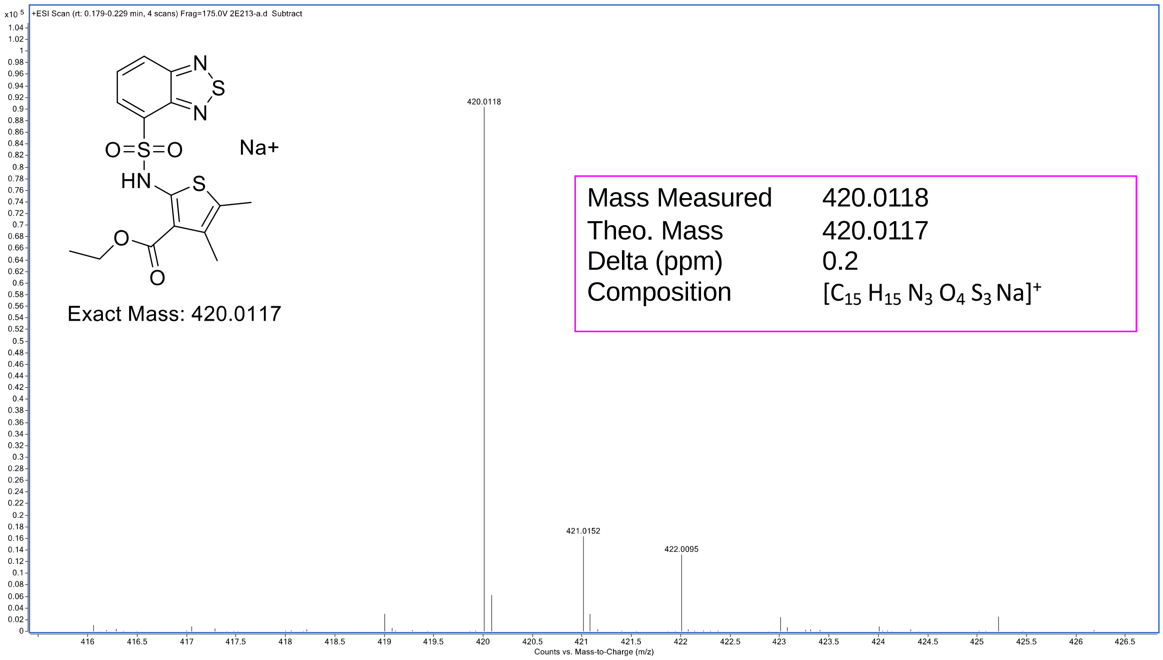

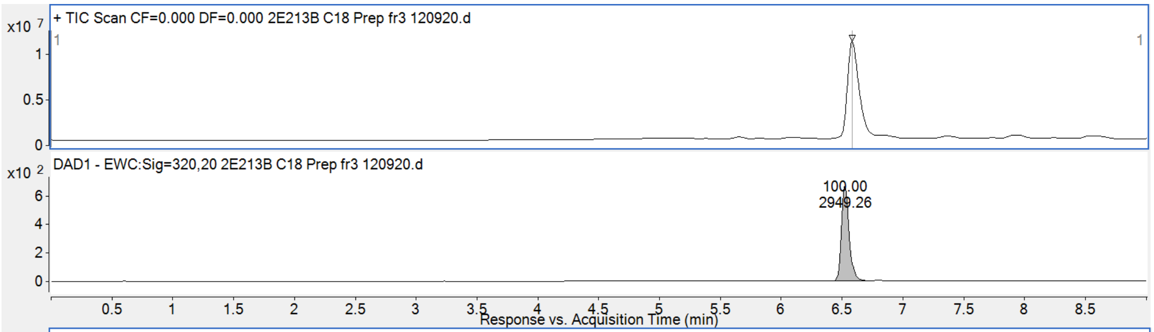

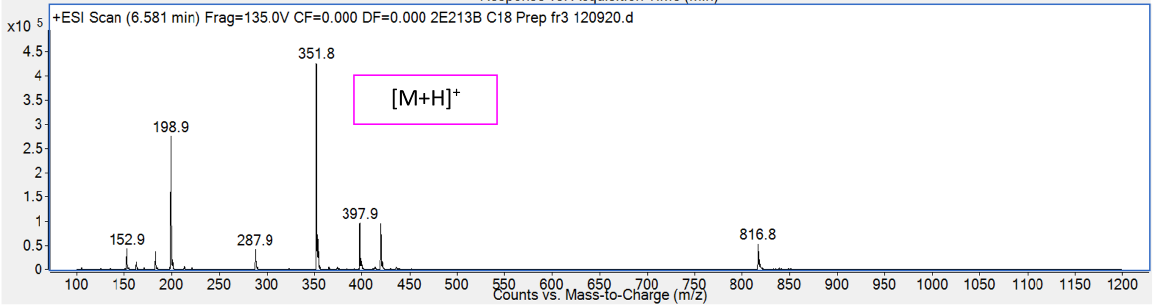

^{1}H, ^{13}C NMR **2H013**

8/8/2022 2:34:09 PM

Formula	$C_{13}H_{11}N_3O_4S_3$	***FW***	369.4391

Acquisition Time (sec)	2.0486	***Comment***	Std proton	***Date***	Jul 13 2022	***Date Stamp***	Jul 13 2022
File Name	C:\Users\Mycoahhh\Documents\NMR\michan\634\2H013-proton.fid\fid					***Frequency (MHz)***	499.83
Nucleus	1H	***Number of Transients***	16	***Original Points Count***	16415	***Points Count***	32768
Pulse Sequence	s2pul	***Receiver Gain***	30.00	***Solvent***	CHLOROFORM-d		
Spectrum Offset (Hz)	2960.0264	***Spectrum Type***	STANDARD	***Sweep Width (Hz)***	8012.82	***Temperature (degree C)***	30.000

^{1}H NMR (500 MHz, CHLOROFORM-d) d 11.00 (s, 1H), 8.29 (d, *J* = 6.85 Hz, 1H), 8.20 (d, *J* = 8.56 Hz, 1H), 7.66 (t, *J* = 7.80 Hz, 1H), 2.14 (s, 6H)

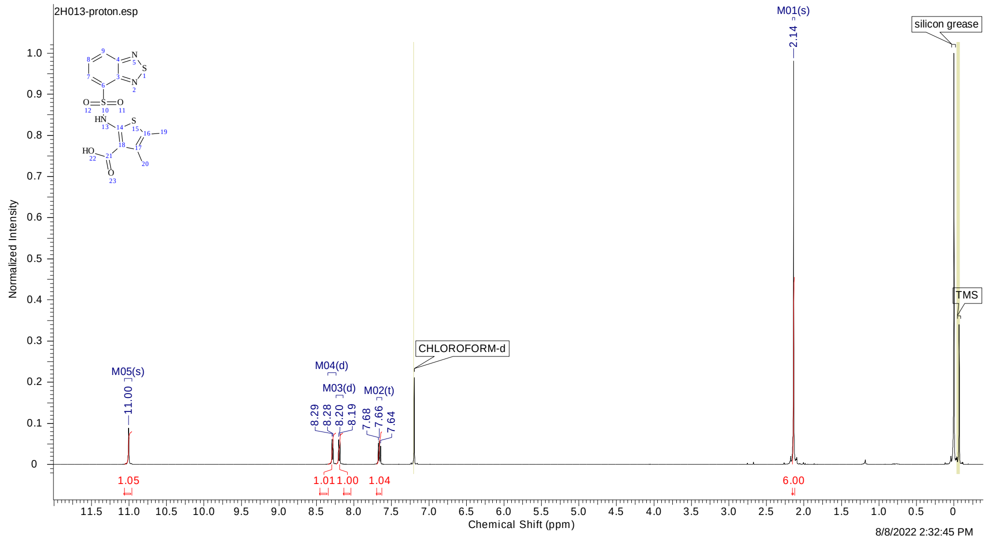

8/8/2022 2:32:45 PM

Formula	$C_{13}H_{11}N_3O_4S_3$	***FW***	369.4391

Acquisition Time (sec)	1.3005	***Comment***	Std carbon	***Date***	Jul 13 2022	***Date Stamp***	Jul 13 2022
File Name	C:\Users\Mycoahhh\Documents\NMR\michan\634\2H013-carbon.fid\fid					***Frequency (MHz)***	125.69
Nucleus	13C	***Number of Transients***	148	***Original Points Count***	39649	***Points Count***	65536
Pulse Sequence	s2pul	***Receiver Gain***	30.00	***Solvent***	CHLOROFORM-d		
Spectrum Offset (Hz)	13192.3086	***Spectrum Type***	STANDARD	***Sweep Width (Hz)***	30487.80	***Temperature (degree C)***	30.000

^{13}C NMR (126 MHz, CHLOROFORM-d) d 169.9, 155.2, 148.7, 148.3, 132.1, 130.8, 129.8, 127.9, 127.4, 123.3, 113.1, 14.4, 12.5

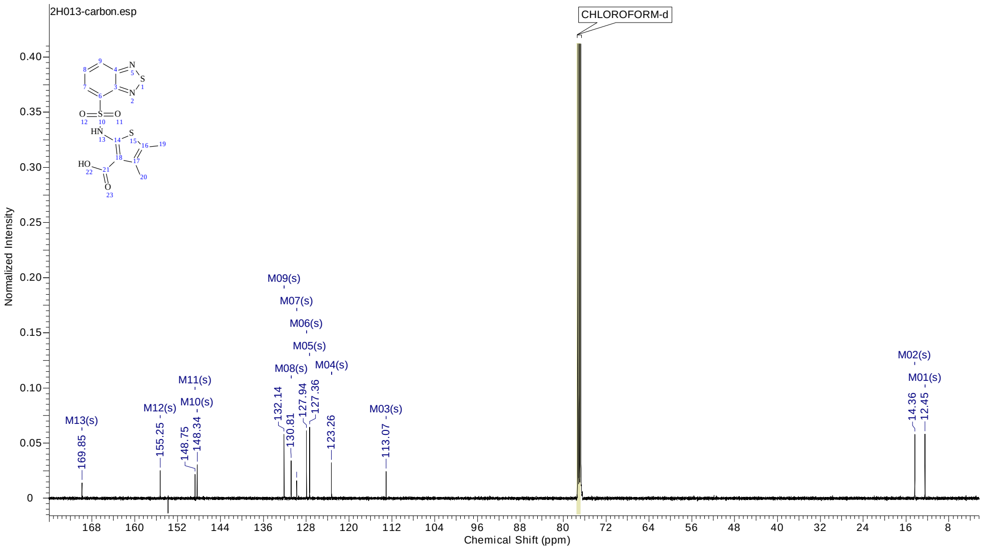

HRMS and LC-MS **2H013**

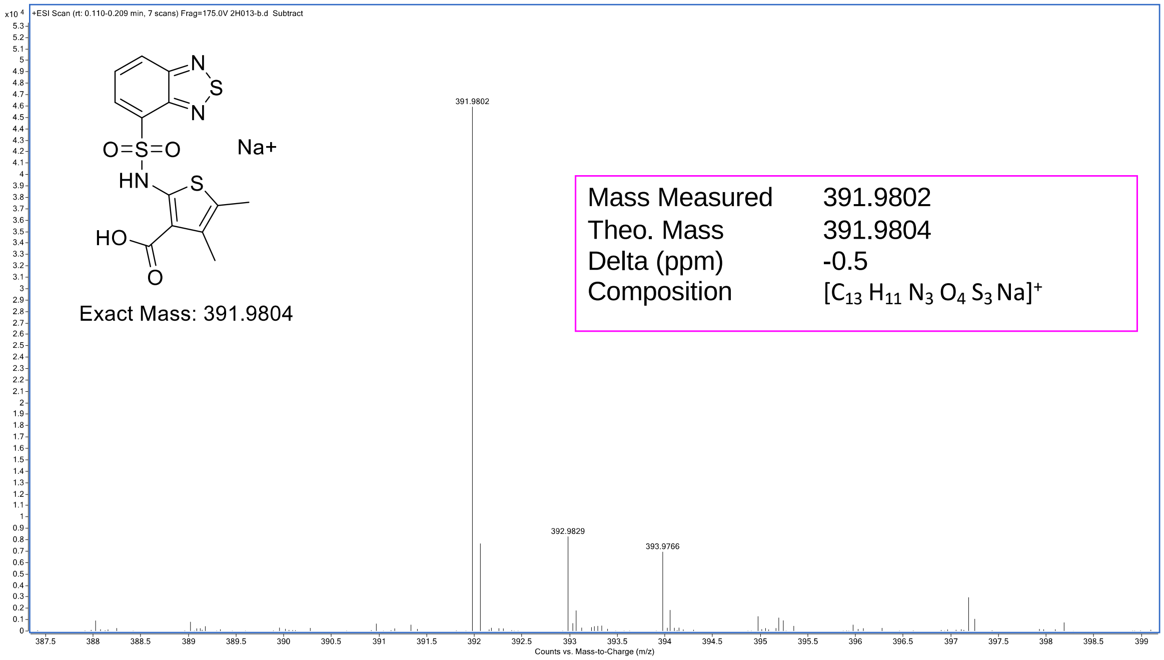

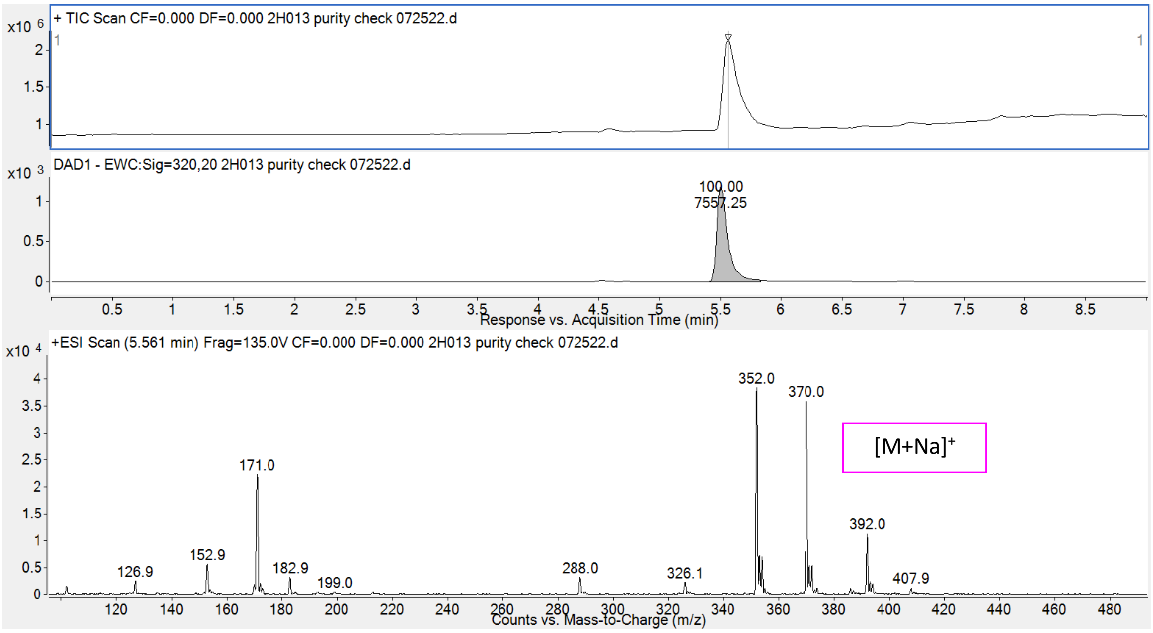

^{1}H, ^{13}C NMR **2F186**

8/9/2022 10:32:29 AM

Formula	$C_{16}H_{17}N_3O_4S_3$	FW	411.5189

Acquisition Time (sec)	2.0486	Comment	Std proton	Date	Jul 19 2022	Date Stamp	Jul 19 2022
File Name	C:\Users\Masy\Desktop\634 NMR\2F186new-proton.fid\fid					Frequency (MHz)	499.83
Nucleus	1H	Number of Transients	16	Original Points Count	16415	Points Count	32768
Pulse Sequence	s2pul	Receiver Gain	20.00	Solvent	CHLOROFORM-d		
Spectrum Offset (Hz)	2999.0005	Spectrum Type	STANDARD	Sweep Width (Hz)	8012.82	Temperature (degree C)	30.000

^{1}H NMR (500 MHz, CHLOROFORM-d) d 8.25 (d, *J* = 8.80 Hz, 1H), 8.13 (d, *J* = 7.00 Hz, 1H), 7.66 (t, *J* = 8.60 Hz, 1H), 3.97 (q, *J* = 7.09 Hz, 2H), 3.64 (s, 3H), 2.21 (s, 3H), 2.16 (s, 3H), 1.26 (t, *J* = 7.21 Hz, 3H)

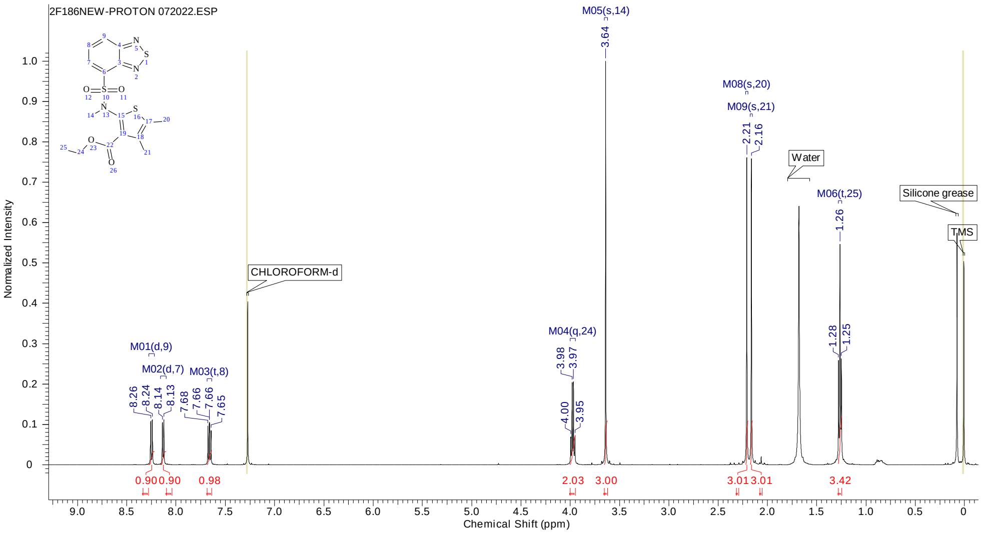

8/8/2022 2:35:00 PM

Formula	$C_{16}H_{17}N_3O_4S_3$	FW	411.5189

Acquisition Time (sec)	1.3005	Comment	Std carbon	Date	Jul 7 2022	Date Stamp	Jul 7 2022
File Name	C:\Users\Mycoahhh\Documents\NMR\michan\634\2F186-2-carbon.fid\fid					Frequency (MHz)	125.69
Nucleus	13C	Number of Transients	128	Original Points Count	39649	Points Count	65536
Pulse Sequence	s2pul	Receiver Gain	30.00	Solvent	CHLOROFORM-d		
Spectrum Offset (Hz)	13192.7734	Spectrum Type	STANDARD	Sweep Width (Hz)	30487.80	Temperature (degree C)	30.000

^{13}C NMR (126 MHz, CHLOROFORM-d) d 163.1, 155.5, 149.7, 140.8, 132.4, 132.4, 131.7, 131.3, 128.2, 126.5, 60.7, 41.9, 14.0, 13.3, 13.3

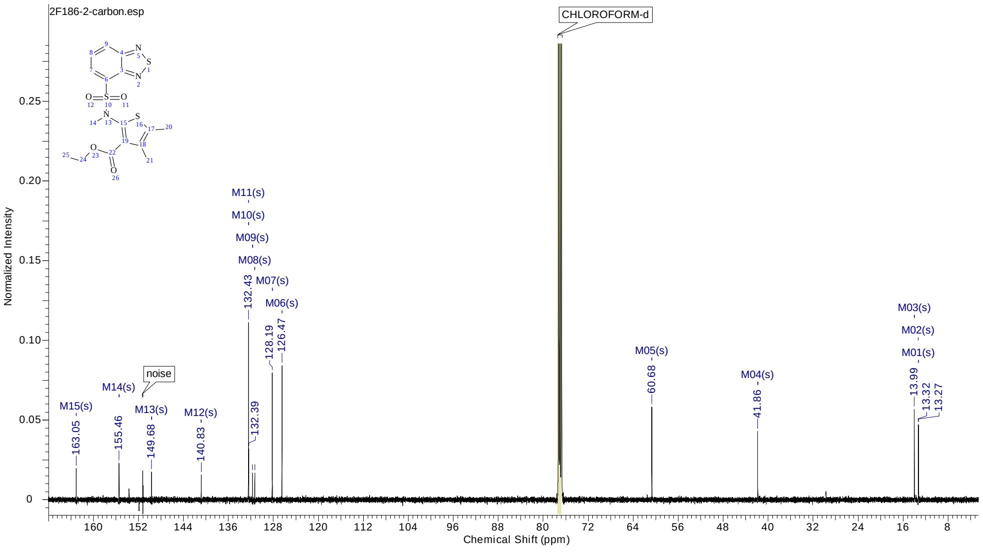

HRMS and LC-MS **2F186**

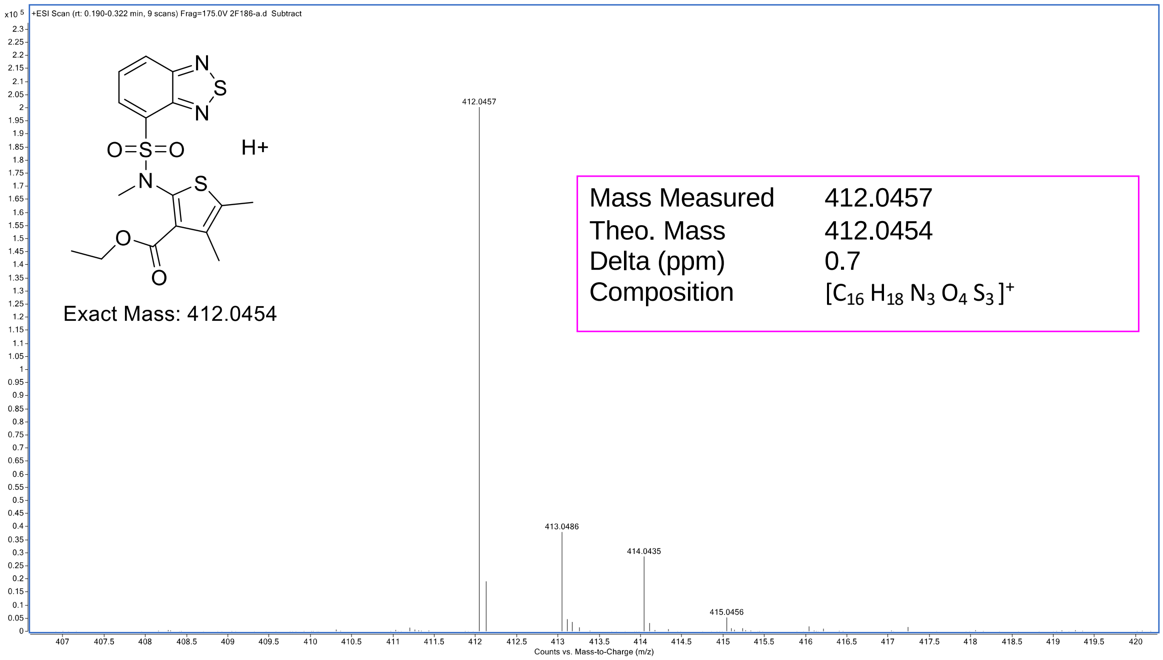

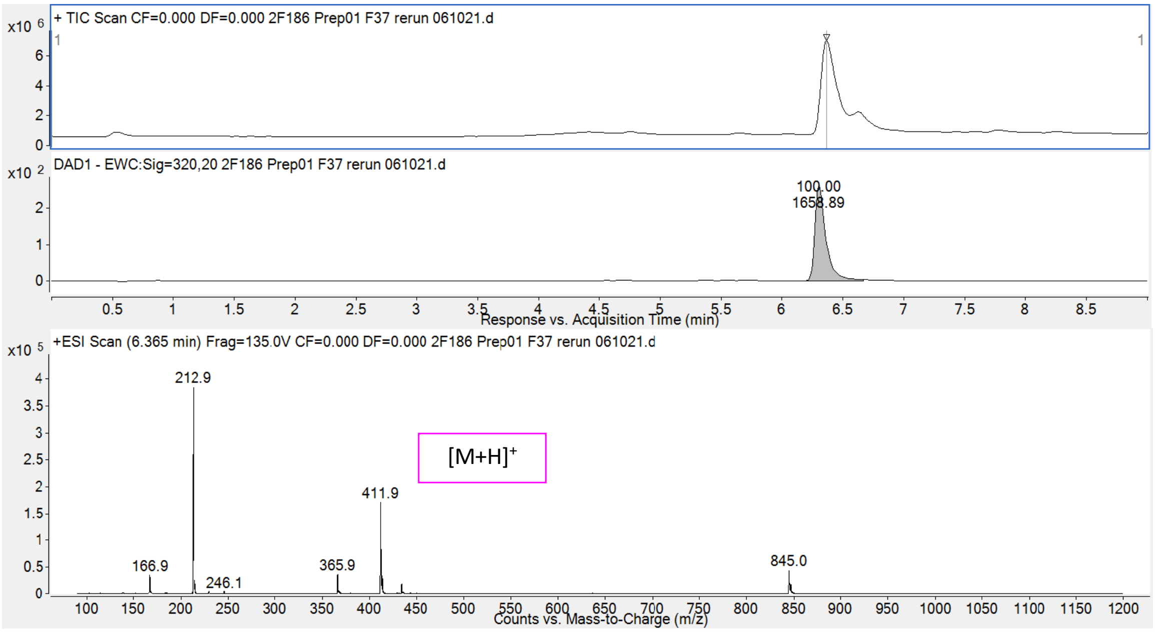

^{1}H, ^{13}C NMR **2E241**

8/8/2022 2:37:40 PM

Formula	$C_{15}H_{16}N_4O_3S_3$	FW	396.5075

Acquisition Time (sec)	2.0486	Comment	Std proton	Date	Jul 19 2022	Date Stamp	Jul 19 2022
File Name	C:\Users\Masy\Desktop\634 NMR\2E241new-proton.fid\fid					Frequency (MHz)	499.83
Nucleus	1H	Number of Transients	16	Original Points Count	16415	Points Count	32768
Pulse Sequence	s2pul	Receiver Gain	20.00	Solvent	CHLOROFORM-d		
Spectrum Offset (Hz)	2995.7290	Spectrum Type	STANDARD	Sweep Width (Hz)	8012.82	Temperature (degree C)	30.000

^{1}H NMR (500 MHz, CHLOROFORM-d) d 10.01 (br. s., 1H), 8.26 (d, J = 4.16 Hz, 1H), 8.25 (d, J = 2.20 Hz, 1H), 7.70 (t, J = 7.80 Hz, 1H), 6.22 (br. s., 1H), 3.39 (quin, J = 6.80 Hz, 2H), 2.12 (s, 3H), 2.08 (s, 3H), 1.20 (t, J = 7.21 Hz, 3H)

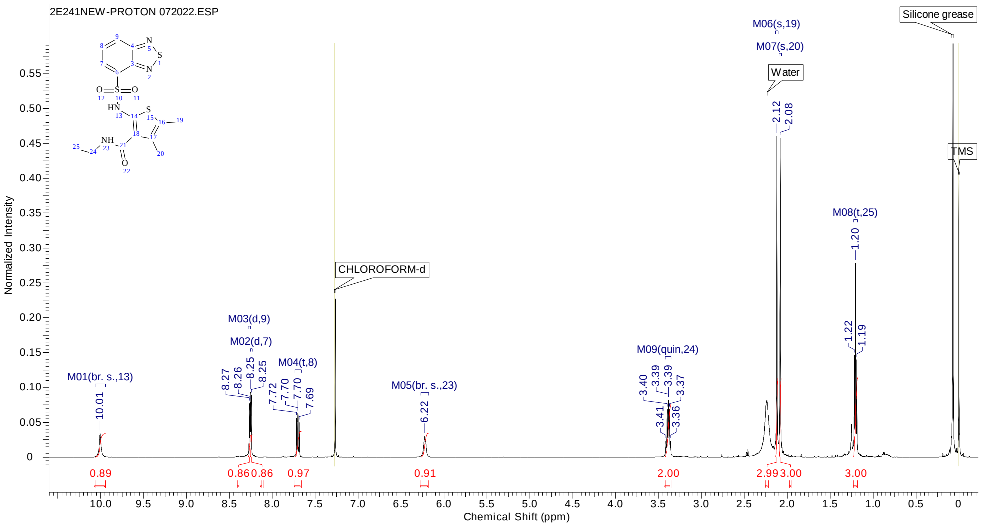

8/8/2022 2:36:41 PM

Formula	$C_{15}H_{16}N_4O_3S_3$	FW	396.5075

Acquisition Time (sec)	1.3005	Comment	Std carbon	Date	Jul 13 2022	Date Stamp	Jul 13 2022
File Name	C:\Users\Mycoahhh\Documents\NMR\michan\634\2E241-2-carbon.fid\fid					Frequency (MHz)	125.69
Nucleus	13C	Number of Transients	168	Original Points Count	39649	Points Count	65536
Pulse Sequence	s2pul	Receiver Gain	30.00	Solvent	CHLOROFORM-d		
Spectrum Offset (Hz)	13192.3086	Spectrum Type	STANDARD	Sweep Width (Hz)	30487.80	Temperature (degree C)	30.000

^{13}C NMR (126 MHz, CHLOROFORM-d) d 164.8, 155.2, 149.0, 136.7, 132.0, 130.0, 128.3, 128.1, 127.0, 126.2, 34.7, 14.7, 13.5, 12.8

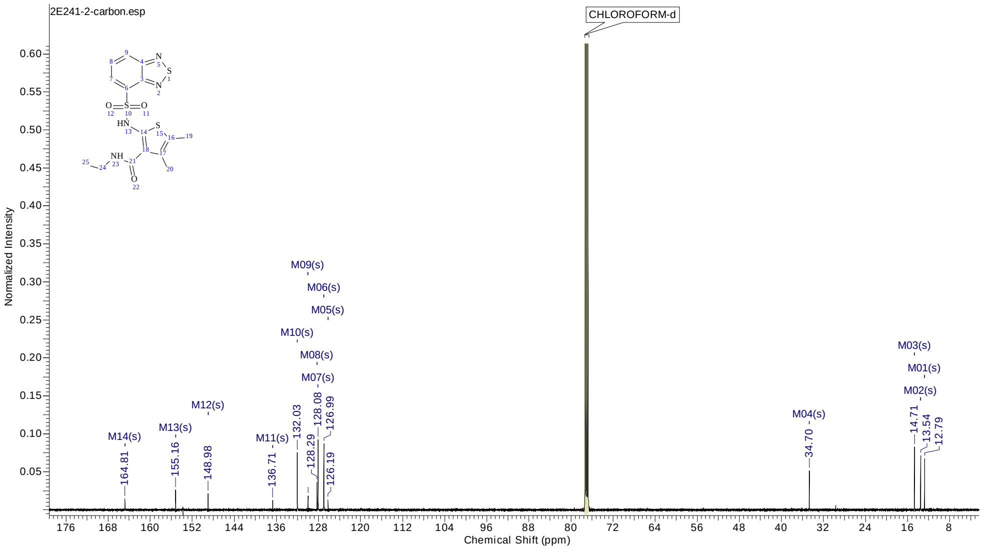

HRMS and LC-MS **2E241**

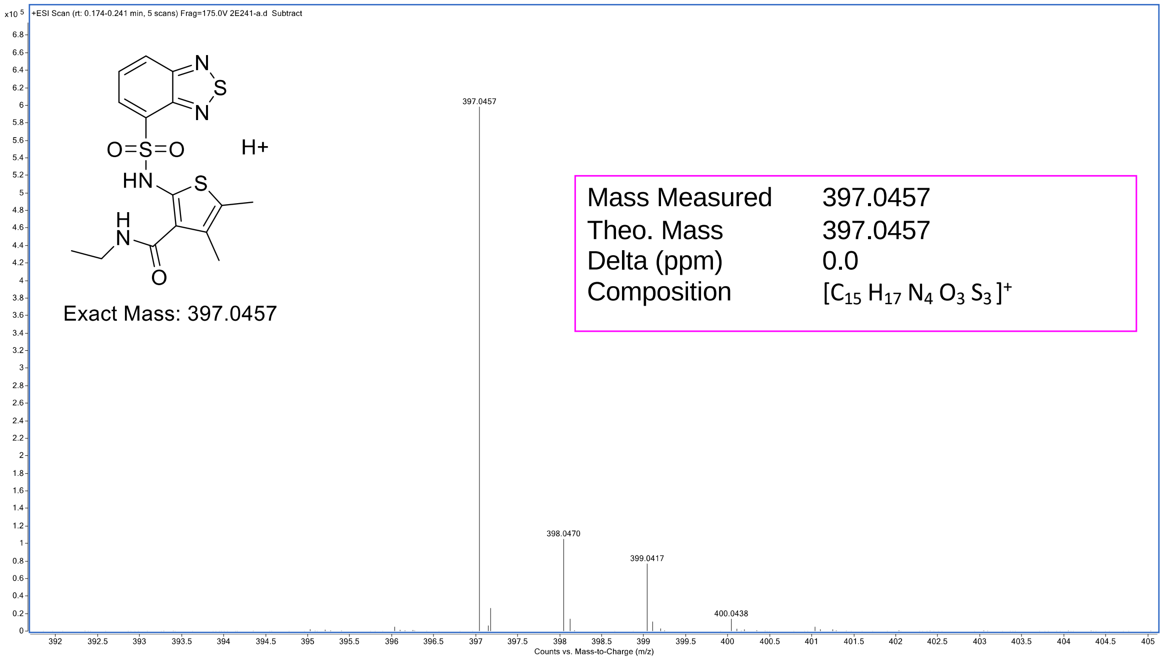

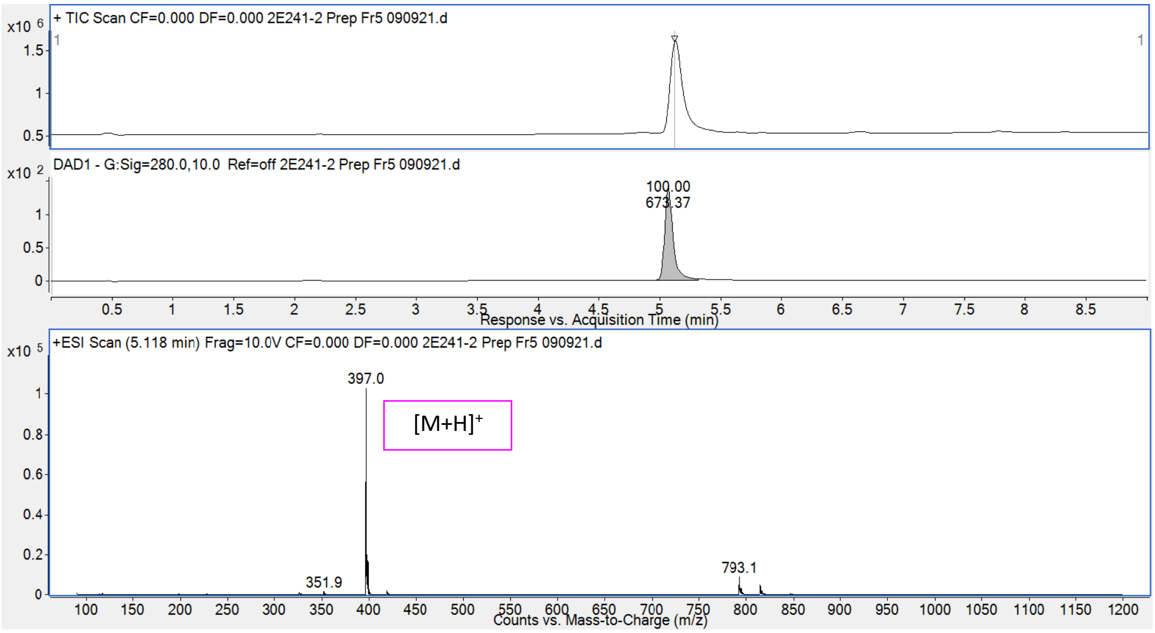

^{1}H, ^{13}C NMR **2G176**

8/8/2022 2:39:29 PM

Formula	$C_{16}H_{17}N_3O_4S_3$	FW	411.5189

Acquisition Time (sec)	2.0486	Comment	Std proton	Date	Jun 28 2022	Date Stamp	Jun 28 2022
File Name	C:\Users\Mycoahhh\Documents\NMR\michan\634\2G176-2-proton.fid\fid					Frequency (MHz)	499.83
Nucleus	1H	Number of Transients	16	Original Points Count	16415	Points Count	32768
Pulse Sequence	s2pul	Receiver Gain	20.00	Solvent	CHLOROFORM-d		
Spectrum Offset (Hz)	2997.1963	Spectrum Type	STANDARD	Sweep Width (Hz)	8012.82	Temperature (degree C)	30.000

^{1}H NMR (500 MHz, CHLOROFORM-d) d 11.15 (s, 1H), 8.34 (d, J = 7.09 Hz, 1H), 8.16 - 8.28 (m, 1H), 7.70 (dd, J = 7.09, 8.80 Hz, 1H), 4.18 (t, J = 6.60 Hz, 2H), 2.16 (s, 3H), 2.08 (s, 3H), 1.69 - 1.79 (m, 2H), 0.98 (t, J = 7.46 Hz, 3H)

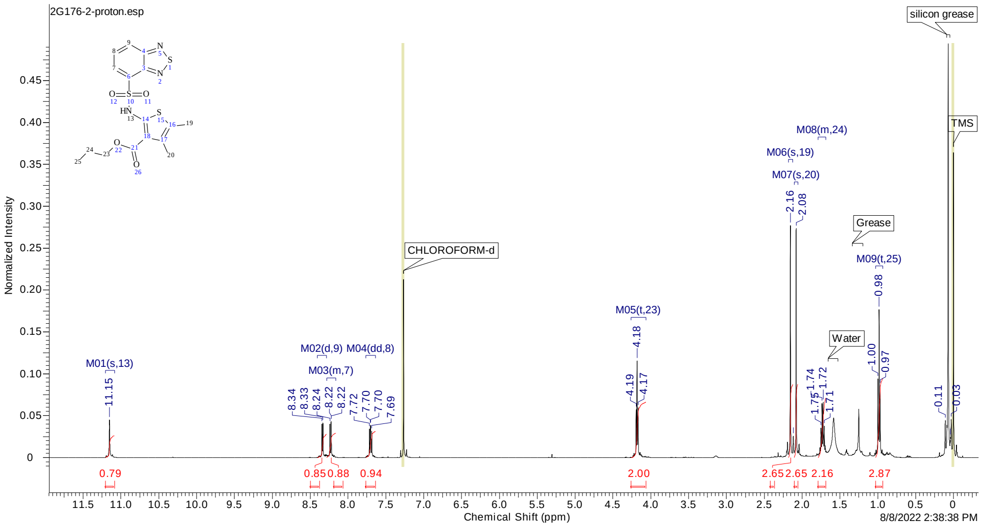

8/8/2022 2:38:38 PM

Formula	$C_{16}H_{17}N_3O_4S_3$	FW	411.5189

Acquisition Time (sec)	1.3005	Comment	Std carbon	Date	Jun 28 2022	Date Stamp	Jun 28 2022
File Name	C:\Users\Mycoahhh\Documents\NMR\michan\634\2G176-2-carbon.fid\fid					Frequency (MHz)	125.69
Nucleus	13C	Number of Transients	116	Original Points Count	39649	Points Count	65536
Pulse Sequence	s2pul	Receiver Gain	30.00	Solvent	CHLOROFORM-d		
Spectrum Offset (Hz)	13193.2383	Spectrum Type	STANDARD	Sweep Width (Hz)	30487.80	Temperature (degree C)	30.000

^{13}C NMR (126 MHz, CHLOROFORM-d) d 165.3, 155.2, 148.9, 145.6, 132.2, 130.3, 130.0, 127.9, 127.1, 122.9, 114.5, 66.5, 21.9, 14.3, 12.4, 10.7

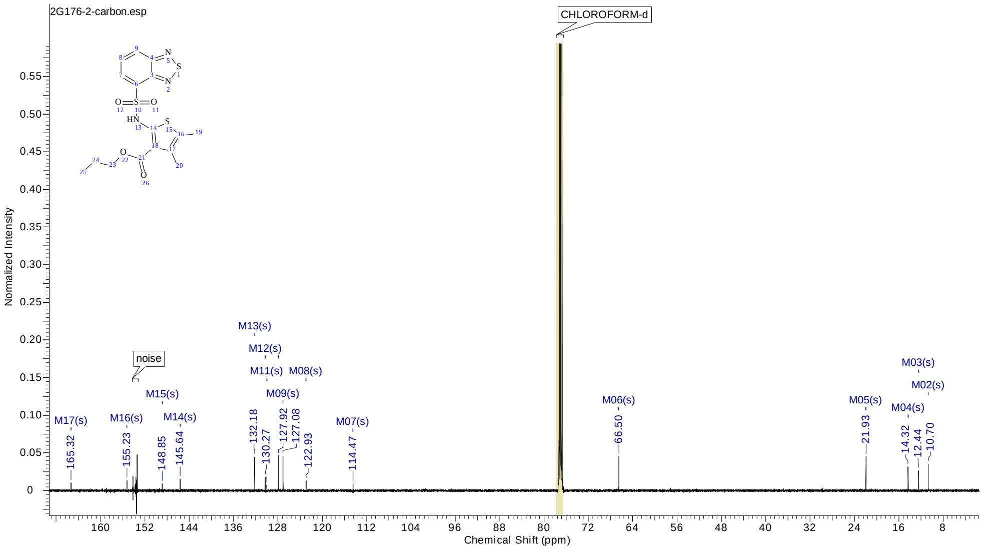

HRMS and LC-MS **2G176**

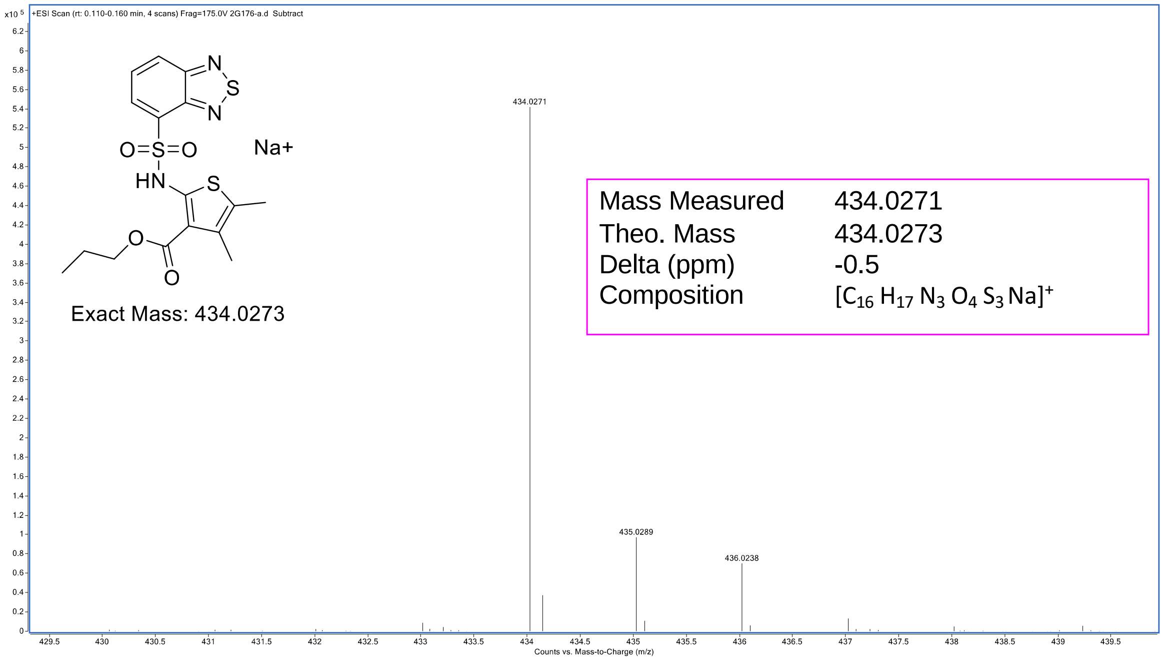

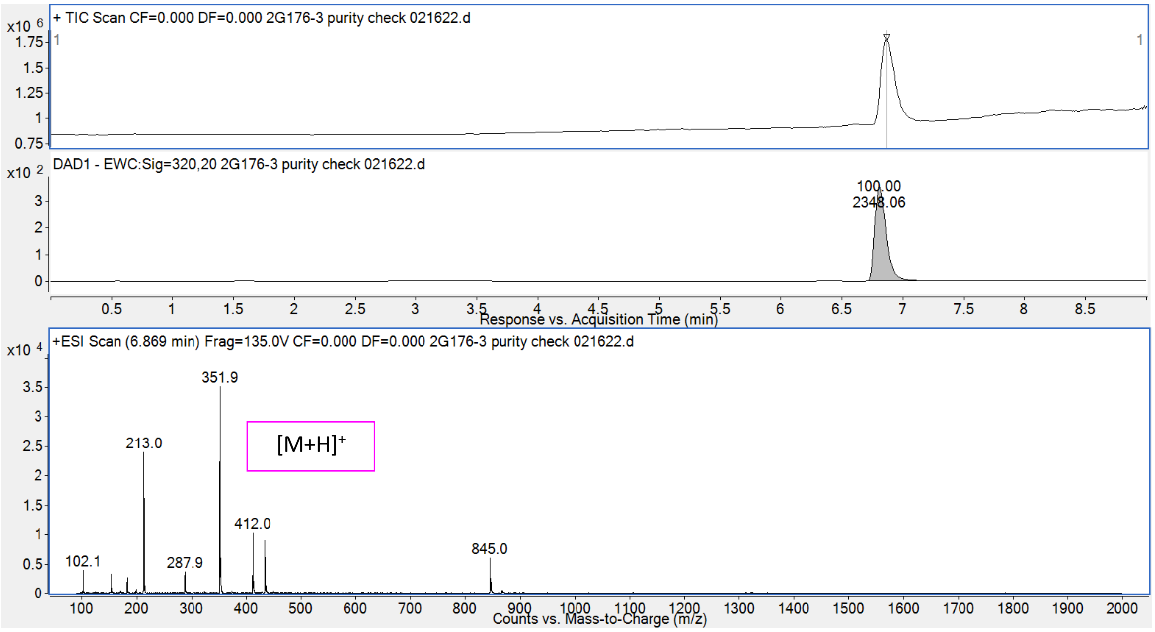

^{1}H, ^{13}C NMR **2G179a**

8/8/2022 2:40:25 PM

Formula	$C_{14}H_{13}N_3O_4S_3$	FW	383.4657

Acquisition Time (sec)	2.0486	Comment	Std proton	Date	Jul 6 2022	Date Stamp	Jul 6 2022
File Name	C:\Users\Mycoahhh\Documents\NMR\michan\634\2G179A-proton.fid\fid					Frequency (MHz)	499.83
Nucleus	1H	Number of Transients	16	Original Points Count	16415	Points Count	32768
Pulse Sequence	s2pul	Receiver Gain	36.00	Solvent	CHLOROFORM-d		
Spectrum Offset (Hz)	2997.0803	Spectrum Type	STANDARD	Sweep Width (Hz)	8012.82	Temperature (degree C)	30.000

^{1}H NMR (500 MHz, CHLOROFORM-d) d 10.99 (s, 1H), 8.34 (d, *J* = 7.00 Hz, 1H), 8.24 (d, *J* = 8.56 Hz, 1H), 7.71 (t, *J* = 8.80 Hz, 1H), 3.82 (s, 3H), 2.16 (s, 3H), 2.07 (s, 3H)

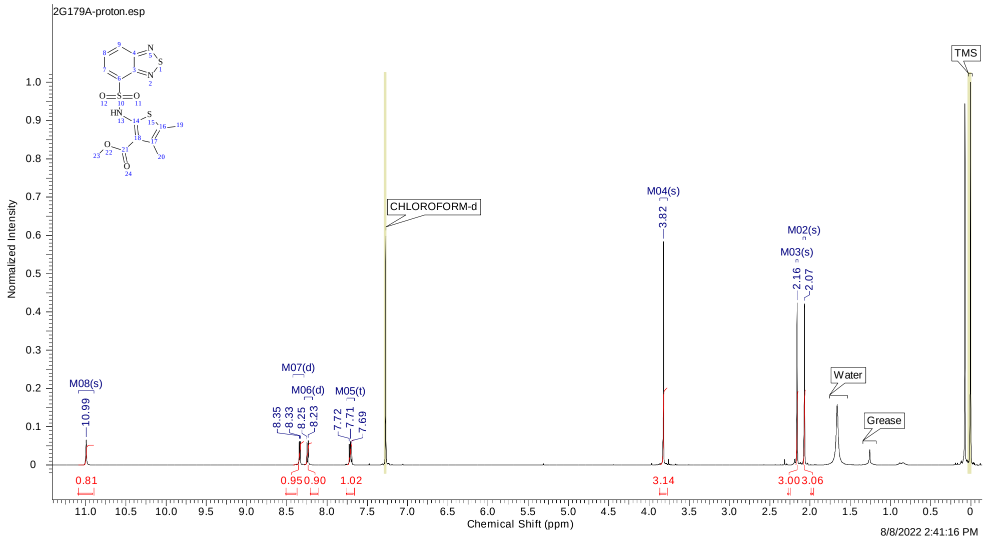

8/8/2022 2:41:16 PM

Formula	$C_{14}H_{13}N_3O_4S_3$	FW	383.4657

Acquisition Time (sec)	1.3005	Comment	Std carbon	Date	Jul 6 2022	Date Stamp	Jul 6 2022
File Name	C:\Users\Mycoahhh\Documents\NMR\michan\634\2G179A-carbon.fid\fid					Frequency (MHz)	125.69
Nucleus	13C	Number of Transients	252	Original Points Count	39649	Points Count	65536
Pulse Sequence	s2pul	Receiver Gain	30.00	Solvent	CHLOROFORM-d		
Spectrum Offset (Hz)	13192.3086	Spectrum Type	STANDARD	Sweep Width (Hz)	30487.80	Temperature (degree C)	30.000

^{13}C NMR (126 MHz, CHLOROFORM-d) d 165.4, 155.2, 148.8, 145.5, 132.2, 130.3, 130.0, 127.9, 127.1, 123.1, 114.4, 51.7, 14.2, 12.4

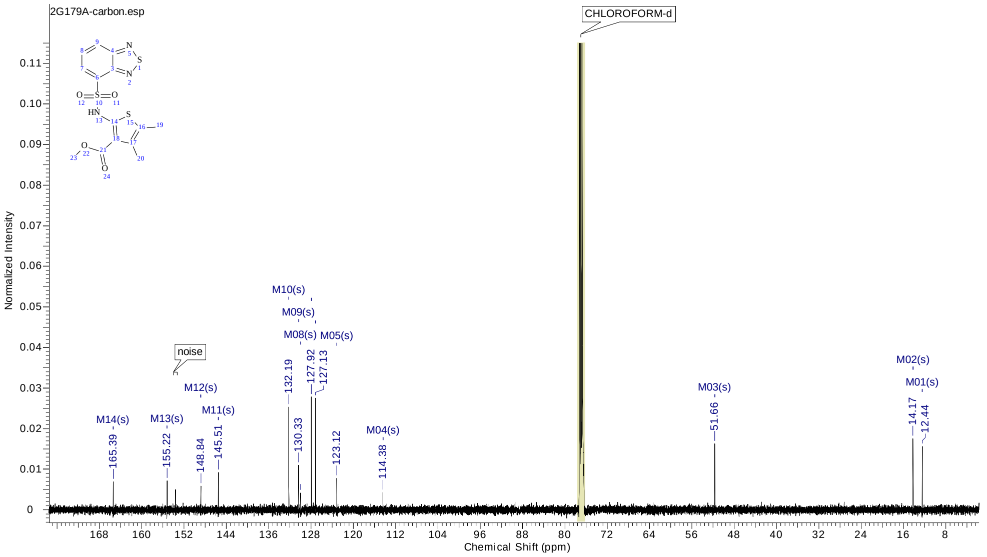

HRMS and LC-MS **2G179a**

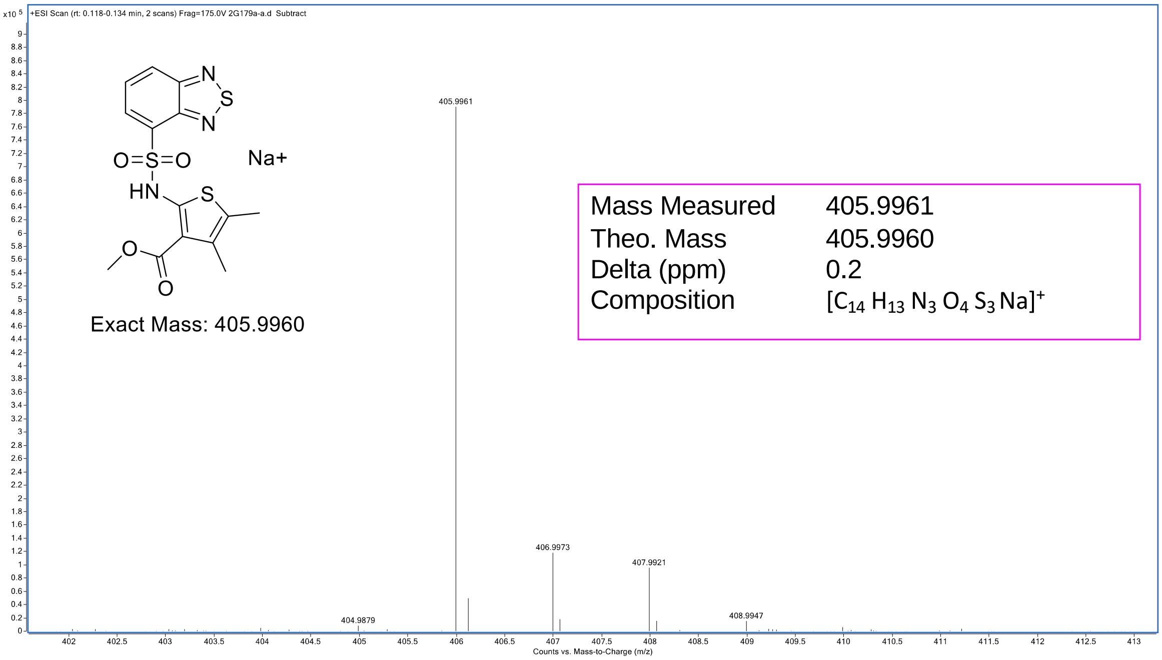

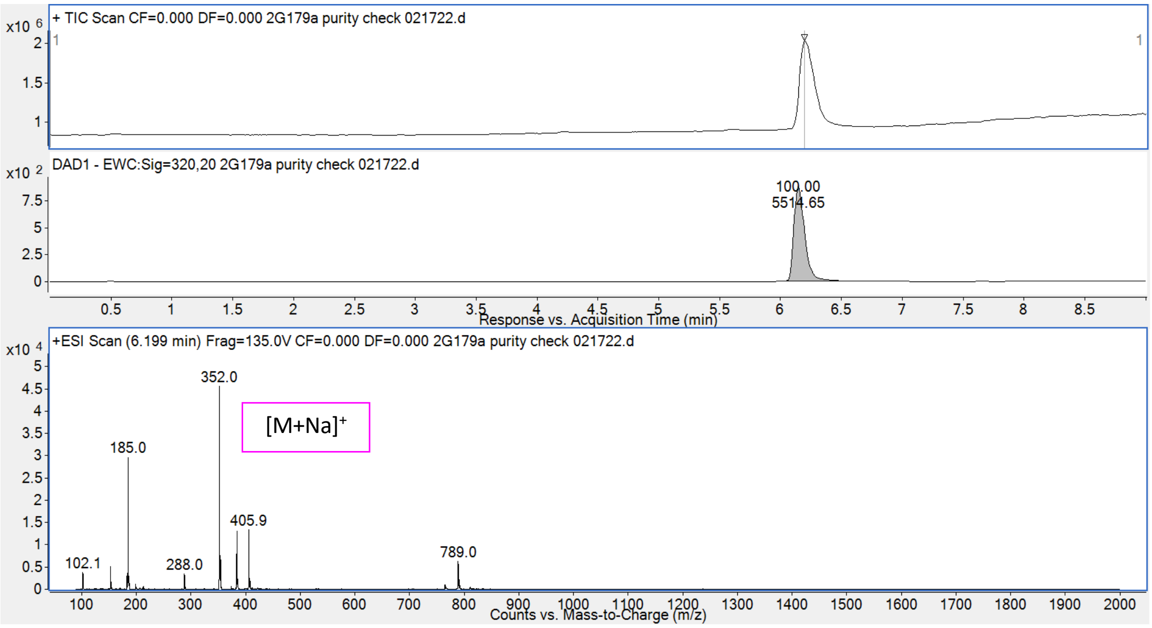

^{1}H, ^{13}C NMR **2G179b**

8/8/2022 2:42:56 PM

Formula	$C_{19}H_{23}N_3O_4S_3$	FW	453.5986

Acquisition Time (sec)	2.0486	Comment	Std proton	Date	Jul 6 2022	Date Stamp	Jul 6 2022
File Name	C:\Users\Mycoahhh\Documents\NMR\michan\634\2G179B-proton.fid\fid					Frequency (MHz)	499.83
Nucleus	1H	Number of Transients	16	Original Points Count	16415	Points Count	32768
Pulse Sequence	s2pul	Receiver Gain	32.00	Solvent	CHLOROFORM-d		
Spectrum Offset (Hz)	2998.9563	Spectrum Type	STANDARD	Sweep Width (Hz)	8012.82	Temperature (degree C)	30.000

^{1}H NMR (500 MHz, CHLOROFORM-d) d 11.16 (s, 1H), 8.35 (d, J = 7.09 Hz, 1H), 8.24 (d, J = 8.80 Hz, 1H), 7.71 (t, J = 7.80 Hz, 1H), 4.22 (t, J = 6.72 Hz, 2H), 2.16 (s, 3H), 2.08 (s, 3H), 1.70 (quin, J = 7.09 Hz, 2H), 1.36 - 1.42 (m, 2H), 1.30 - 1.35 (m, 4H), 0.90 (t, J = 6.72 Hz, 3H)

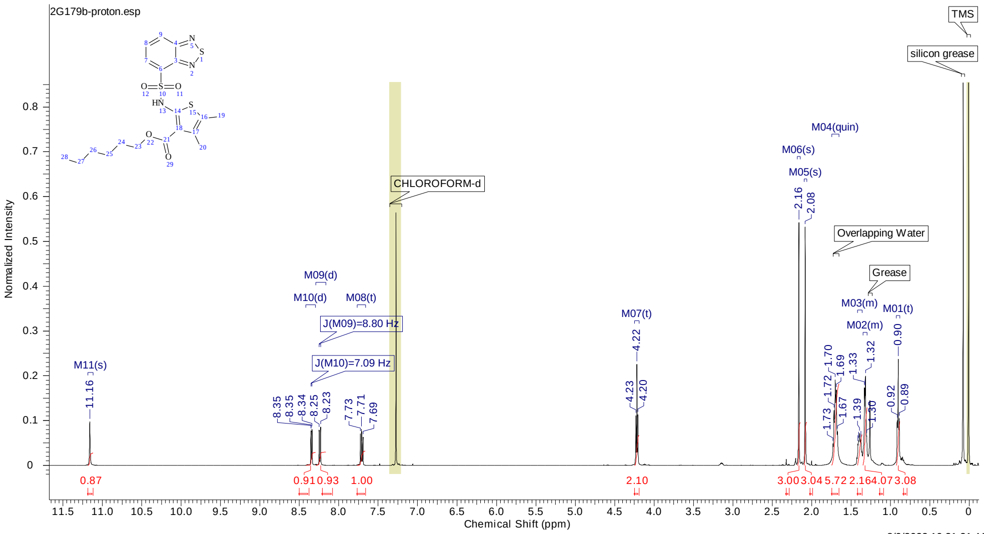

8/9/2022 10:31:21 AM

Formula	$C_{19}H_{23}N_3O_4S_3$	FW	453.5986

Acquisition Time (sec)	1.3005	Comment	Std carbon	Date	Jul 6 2022	Date Stamp	Jul 6 2022
File Name	C:\Users\Mycoahhh\Documents\NMR\michan\634\2G179B-carbon.fid\fid					Frequency (MHz)	125.69
Nucleus	13C	Number of Transients	132	Original Points Count	39649	Points Count	65536
Pulse Sequence	s2pul	Receiver Gain	30.00	Solvent	CHLOROFORM-d		
Spectrum Offset (Hz)	13192.7734	Spectrum Type	STANDARD	Sweep Width (Hz)	30487.80	Temperature (degree C)	30.000

^{13}C NMR (126 MHz, CHLOROFORM-d) d 165.3, 155.2, 148.9, 145.6, 132.2, 130.3, 130.0, 127.9, 127.1, 122.9, 114.5, 65.0, 31.4, 28.5, 25.7, 22.5, 14.3, 14.0, 12.4

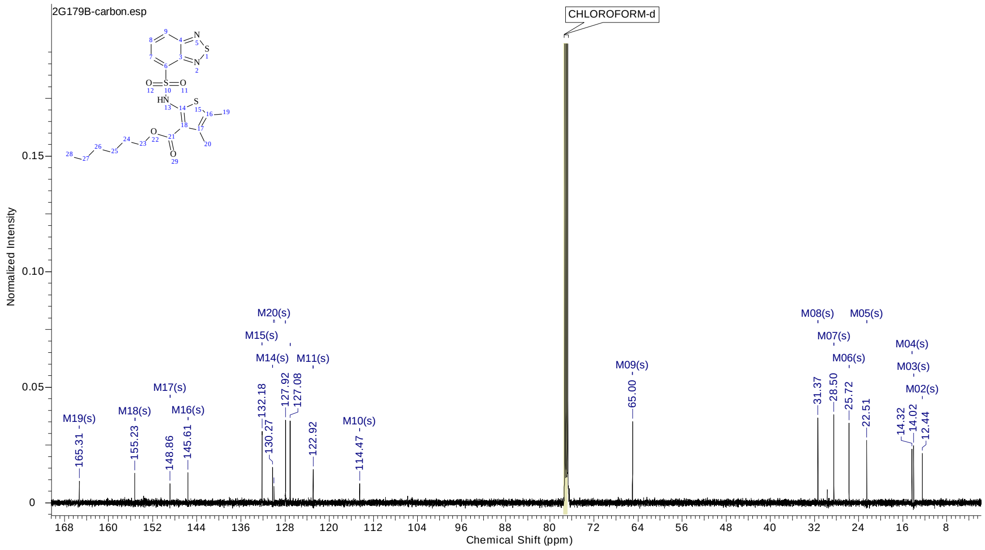

HRMS and LC-MS **2G179b**

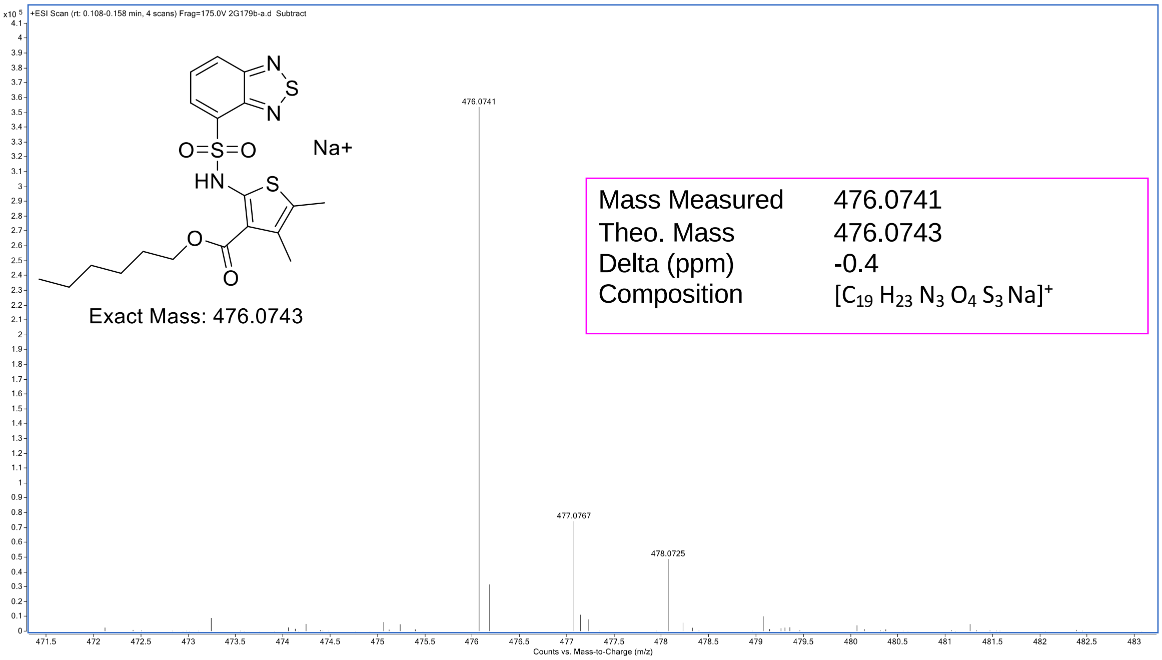

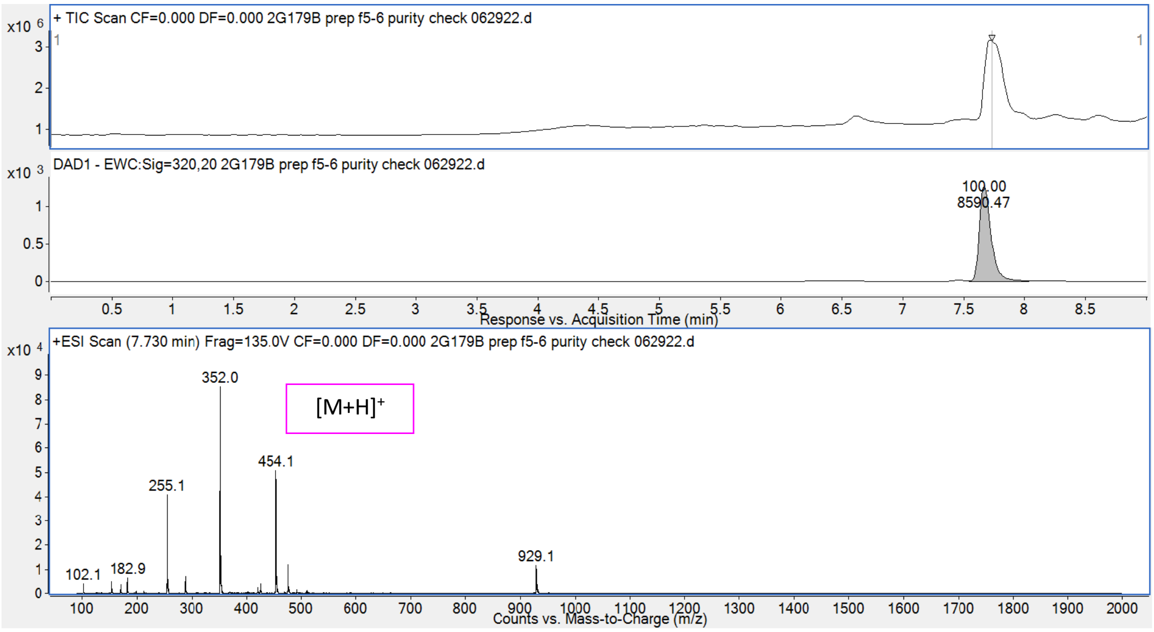

^{1}H, ^{13}C NMR

2G179c

8/8/2022 2:46:39 PM

Formula	$C_{16}H_{17}N_3O_4S_3$	***FW***	411.5189

Acquisition Time (sec)	2.0486	***Comment***	Std proton	***Date***	Jul 6 2022	***Date Stamp***	Jul 6 2022
File Name	C:\Users\Mycoahhh\Documents\NMR\michan\634\2G179C-proton.fid\fid					***Frequency (MHz)***	499.83
Nucleus	1H	***Number of Transients***	16	***Original Points Count***	16415	***Points Count***	32768
Pulse Sequence	s2pul	***Receiver Gain***	20.00	***Solvent***	CHLOROFORM-d		
Spectrum Offset (Hz)	2995.2400	***Spectrum Type***	STANDARD	***Sweep Width (Hz)***	8012.82	***Temperature (degree C)***	30.000

^{1}H NMR (500 MHz, CHLOROFORM-d) d 11.20 (s, 1H), 8.29 - 8.39 (m, 1H), 8.18 - 8.28 (m, 1H), 7.70 (dd, *J* = 7.09, 8.80 Hz, 1H), 5.10 - 5.21 (m, 1H), 2.15 (s, 3H), 2.06 (s, 3H), 1.30 (d, *J* = 6.36 Hz, 6H)

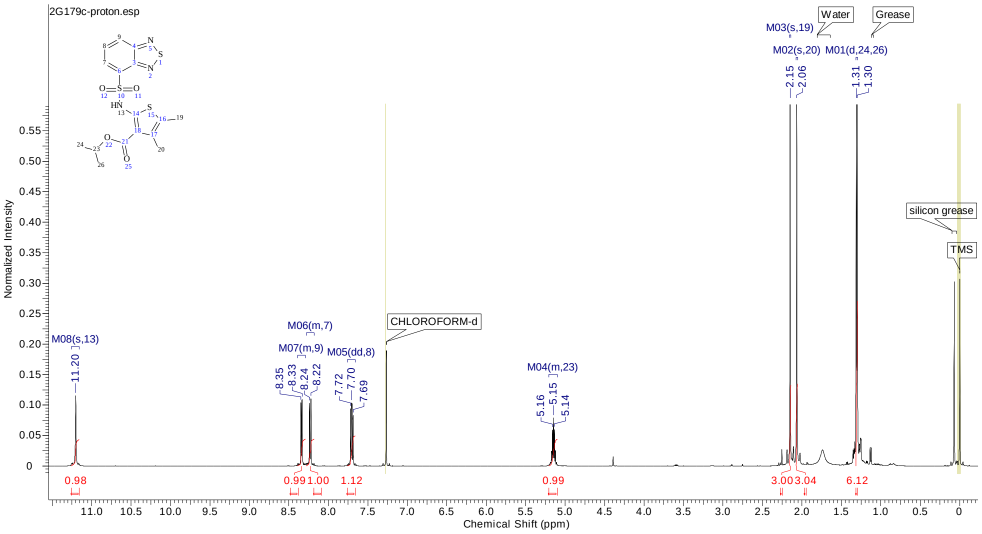

8/8/2022 2:44:15 PM

Formula	$C_{16}H_{17}N_3O_4S_3$	***FW***	411.5189

Acquisition Time (sec)	1.3005	***Comment***	Std carbon	***Date***	Jul 6 2022	***Date Stamp***	Jul 6 2022
File Name	C:\Users\Mycoahhh\Documents\NMR\michan\634\2G179C-carbon.fid\fid					***Frequency (MHz)***	125.69
Nucleus	13C	***Number of Transients***	152	***Original Points Count***	39649	***Points Count***	65536
Pulse Sequence	s2pul	***Receiver Gain***	30.00	***Solvent***	CHLOROFORM-d		
Spectrum Offset (Hz)	13192.3086	***Spectrum Type***	STANDARD	***Sweep Width (Hz)***	30487.80	***Temperature (degree C)***	30.000

^{13}C NMR (126 MHz, CHLOROFORM-d) d 164.8, 155.2, 148.9, 145.4, 132.2, 130.3, 130.0, 127.9, 127.1, 122.9, 114.7, 68.6, 21.9, 14.3, 12.4

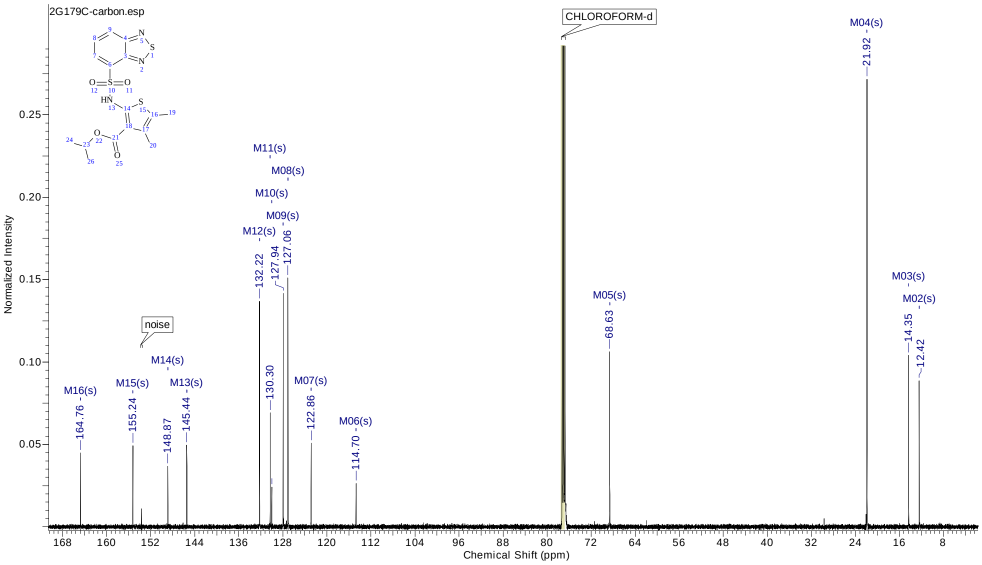

HRMS and LC-MS **2G179c**

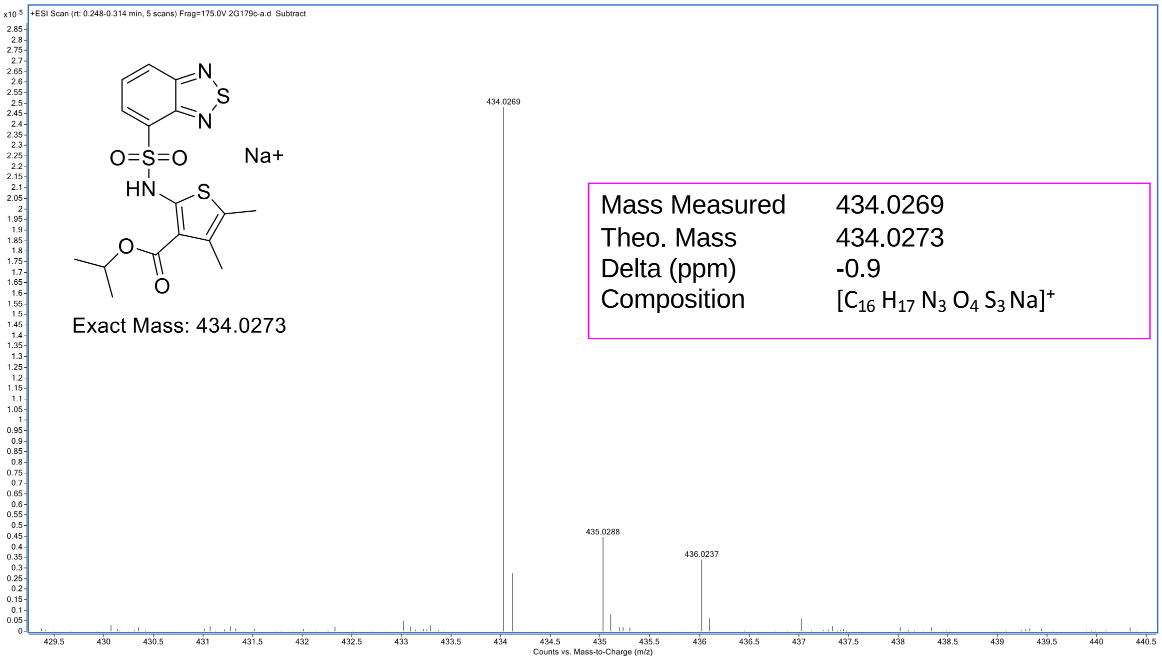

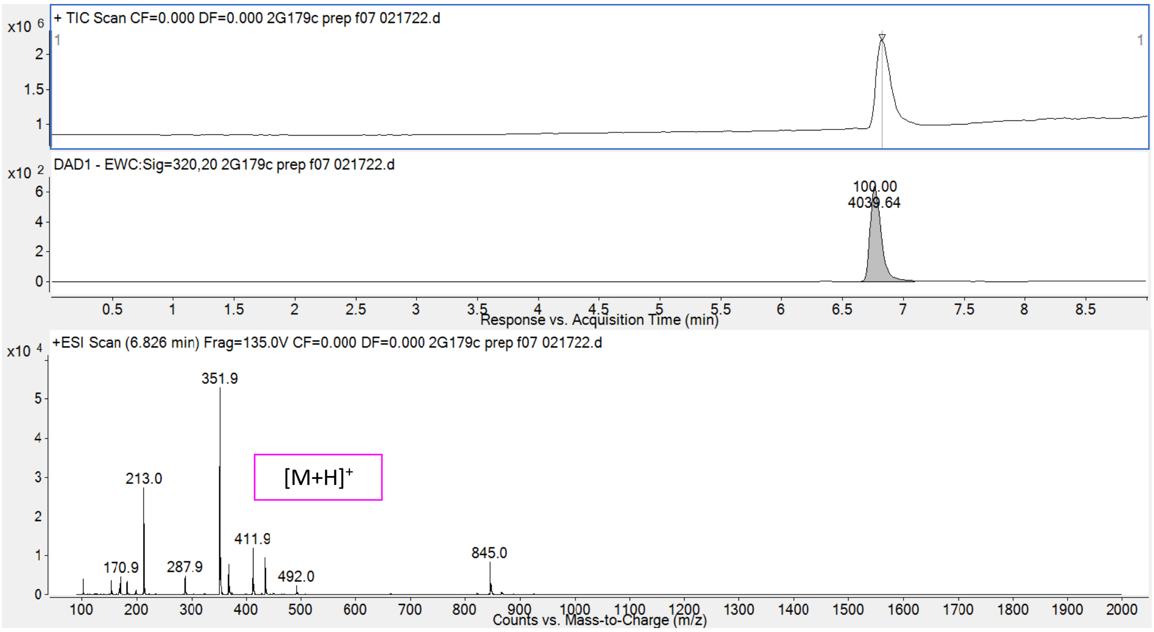

^{1}H, ^{13}C NMR **2G179d**

8/17/2022 8:26:24 AM

Formula	$C_{18}H_{21}N_3O_4S_3$	FW	439.5720

Acquisition Time (sec)	2.0486	***Comment***	Std proton	***Date***	Jul 6 2022	***Date Stamp***	Jul 6 2022
File Name	C:\Users\Mycoahhh\Documents\NMR\michan\634\2G179D-proton.fid\fid					***Frequency (MHz)***	499.83
Nucleus	1H	***Number of Transients***	16	***Original Points Count***	16415	***Points Count***	32768
Pulse Sequence	s2pul	***Receiver Gain***	34.00	***Solvent***	CHLOROFORM-d		
Spectrum Offset (Hz)	2994.7510	***Spectrum Type***	STANDARD	***Sweep Width (Hz)***	8012.82	***Temperature (degree C)***	30.000

^{1}H NMR (500 MHz, CHLOROFORM-d) d 11.14 (s, 1H), 8.34 (d, *J* = 7.09 Hz, 1H), 8.20 - 8.29 (m, 1H), 7.70 (dd, *J* = 7.09, 8.80 Hz, 1H), 4.25 (t, *J* = 6.85 Hz, 2H), 2.15 (s, 3H), 2.07 (s, 3H), 1.68 - 1.76 (m, 1H), 1.59 (q, *J* = 6.93 Hz, 2H), 0.95 (s, 3H), 0.93 (s, 3H)

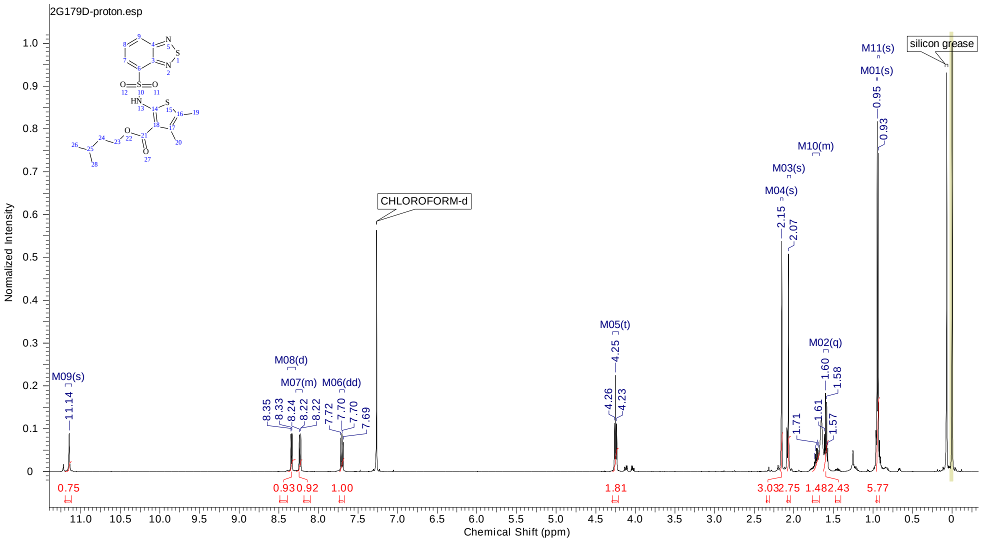

8/17/2022 8:28:17 AM

Formula	$C_{18}H_{21}N_3O_4S_3$	FW	439.5720

Acquisition Time (sec)	1.3005	***Comment***	Std carbon	***Date***	Jul 6 2022	***Date Stamp***	Jul 6 2022
File Name	C:\Users\Mycoahhh\Documents\NMR\michan\634\2G179D-carbon.fid\fid					***Frequency (MHz)***	125.69
Nucleus	13C	***Number of Transients***	140	***Original Points Count***	39649	***Points Count***	65536
Pulse Sequence	s2pul	***Receiver Gain***	30.00	***Solvent***	CHLOROFORM-d		
Spectrum Offset (Hz)	13192.7734	***Spectrum Type***	STANDARD	***Sweep Width (Hz)***	30487.80	***Temperature (degree C)***	30.000

^{13}C NMR (126 MHz, CHLOROFORM-d) d 165.3, 155.2, 152.8, 148.9, 145.6, 132.2, 130.3, 127.9, 127.1, 122.9, 114.4, 63.4, 37.2, 25.1, 22.4, 14.3, 12.4

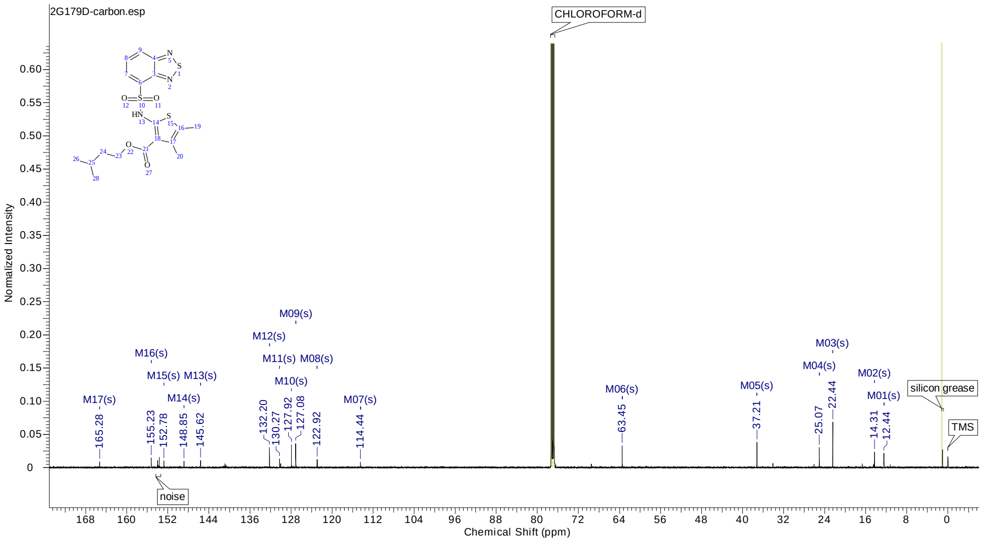

HRMS and LC-MS **2G179d**

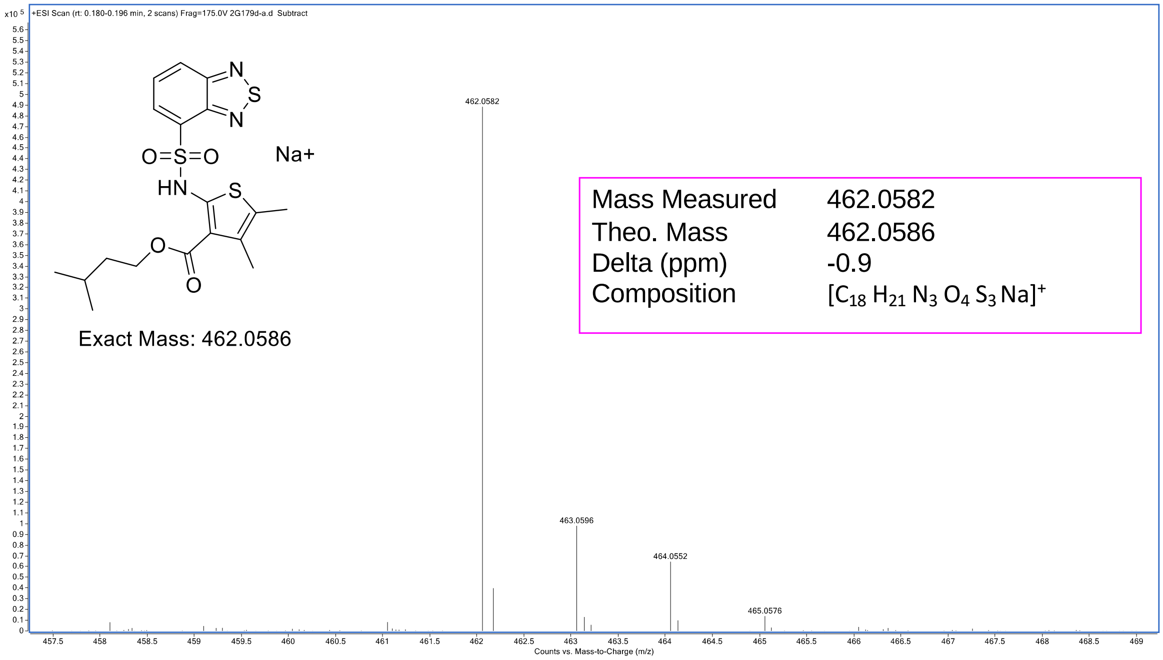

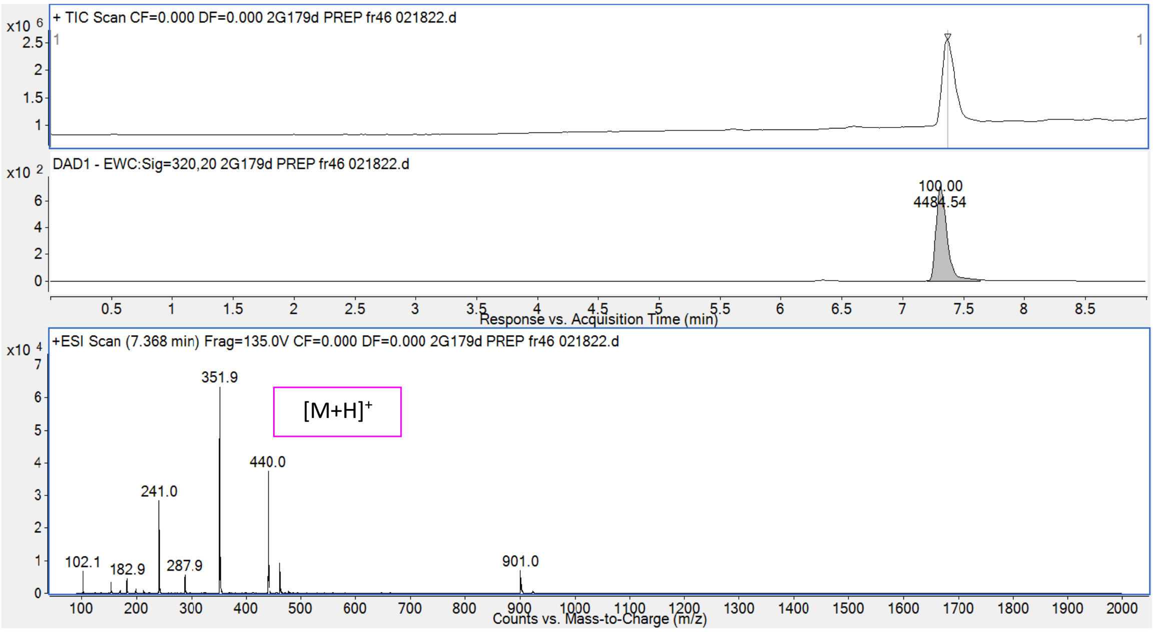

^{1}H, ^{13}C NMR **2G179e**

8/8/2022 3:19:29 PM

Formula	$C_{19}H_{21}N_3O_4S_3$	FW	451.5827

Acquisition Time (sec)	2.0486	Comment	Std proton	Date	Jul 6 2022	Date Stamp	Jul 6 2022
File Name	C:\Users\Mycoahhh\Documents\NMR\michan\634\2G179E-proton.fid\fid					Frequency (MHz)	499.83
Nucleus	1H	Number of Transients	16	Original Points Count	16415	Points Count	32768
Pulse Sequence	s2pul	Receiver Gain	34.00	Solvent	CHLOROFORM-d		
Spectrum Offset (Hz)	2997.0803	Spectrum Type	STANDARD	Sweep Width (Hz)	8012.82	Temperature (degree C)	30.000

^{1}H NMR (500 MHz, CHLOROFORM-d) d 11.22 (s, 1H), 8.30 - 8.40 (m, 1H), 8.18 - 8.28 (m, 1H), 7.71 (dd, *J* = 7.09, 8.80 Hz, 1H), 4.96 (quin, *J* = 4.20 Hz, 1H), 2.16 (s, 3H), 2.09 (s, 3H), 1.84 - 1.93 (m, 2H), 1.71 - 1.77 (m, 2H), 1.37 - 1.60 (m, 6H)

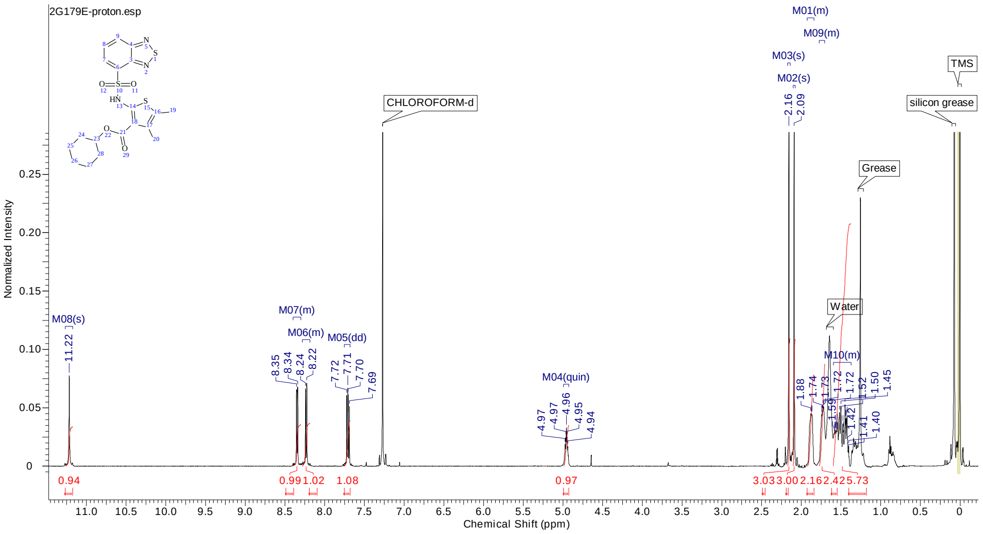

8/8/2022 2:48:50 PM

Formula	$C_{19}H_{21}N_3O_4S_3$	FW	451.5827

Acquisition Time (sec)	1.3005	Comment	Std carbon	Date	Jul 6 2022	Date Stamp	Jul 6 2022
File Name	C:\Users\Mycoahhh\Documents\NMR\michan\634\2G179E-carbon.fid\fid					Frequency (MHz)	125.69
Nucleus	13C	Number of Transients	136	Original Points Count	39649	Points Count	65536
Pulse Sequence	s2pul	Receiver Gain	30.00	Solvent	CHLOROFORM-d		
Spectrum Offset (Hz)	13192.7734	Spectrum Type	STANDARD	Sweep Width (Hz)	30487.80	Temperature (degree C)	30.000

^{13}C NMR (126 MHz, CHLOROFORM-d) d 164.7, 155.2, 148.9, 145.5, 132.2, 130.3, 130.0, 127.9, 127.1, 122.8, 114.8, 73.3, 31.5, 29.7, 25.3, 23.5, 14.5, 12.4

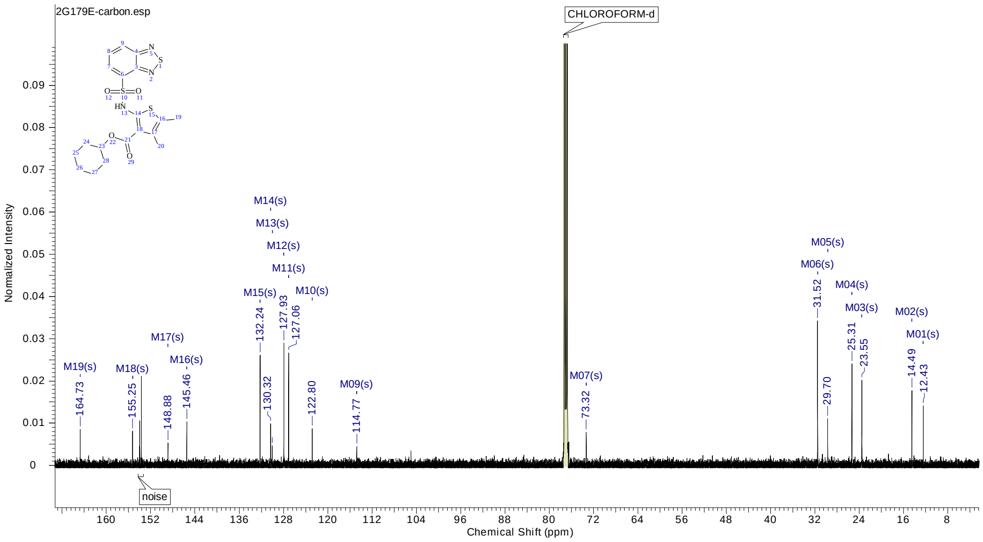

HRMS and LC-MS **2G179e**

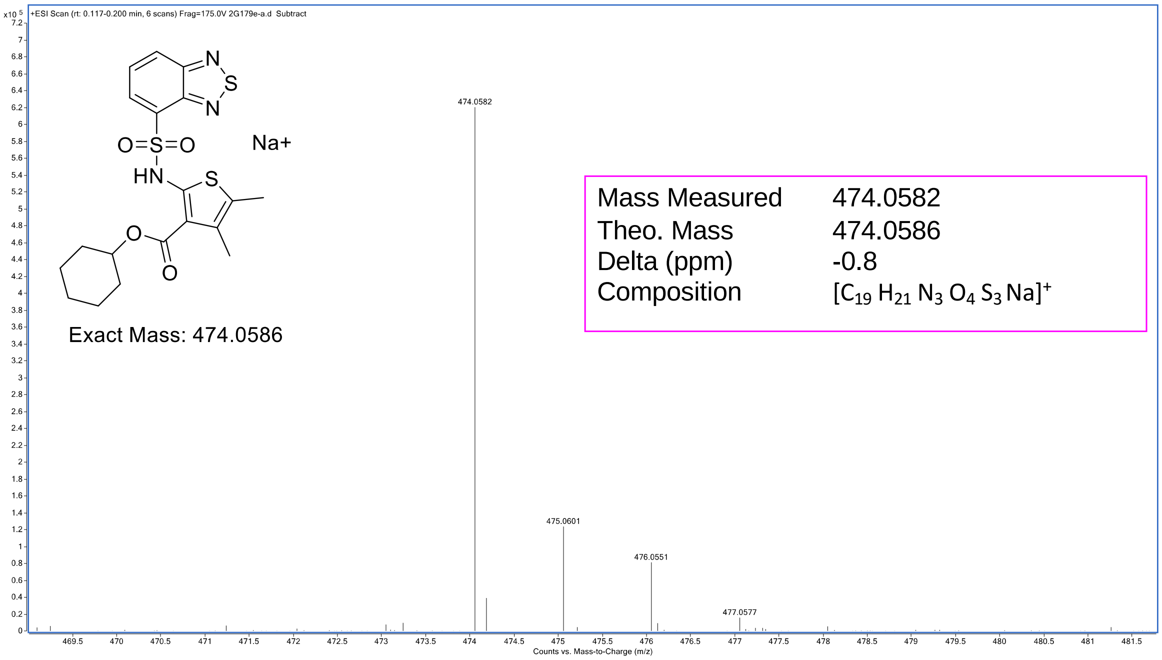

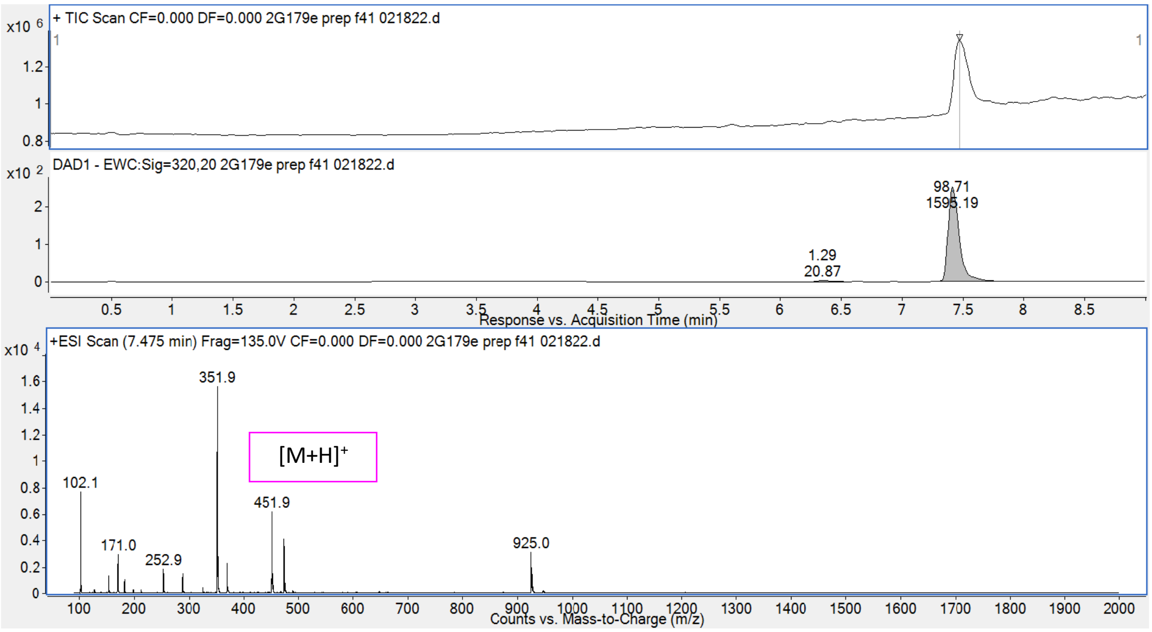

^{1}H, ^{13}C NMR

2G179f

8/8/2022 2:51:43 PM

Formula	$C_{20}H_{17}N_3O_4S_3$	FW	459.5617

Acquisition Time (sec)	2.0486	Comment	Std proton	Date	Jul 6 2022	Date Stamp	Jul 6 2022
File Name	C:\Users\Mycoahhh\Documents\NMR\michan\634\2G179F-proton.fid\fid					Frequency (MHz)	499.83
Nucleus	1H	Number of Transients	16	Original Points Count	16415	Points Count	32768
Pulse Sequence	s2pul	Receiver Gain	36.00	Solvent	CHLOROFORM-d		
Spectrum Offset (Hz)	2994.5063	Spectrum Type	STANDARD	Sweep Width (Hz)	8012.82	Temperature (degree C)	30.000

^{1}H NMR (500 MHz, CHLOROFORM-d) d 11.13 (s, 1H), 8.28 - 8.37 (m, 1H), 8.21 (d, *J* = 8.80 Hz, 1H), 7.69 (dd, *J* = 7.09, 8.56 Hz, 1H), 7.33 - 7.42 (m, 5H), 5.27 (s, 2H), 2.14 (s, 3H), 2.06 (s, 3H)

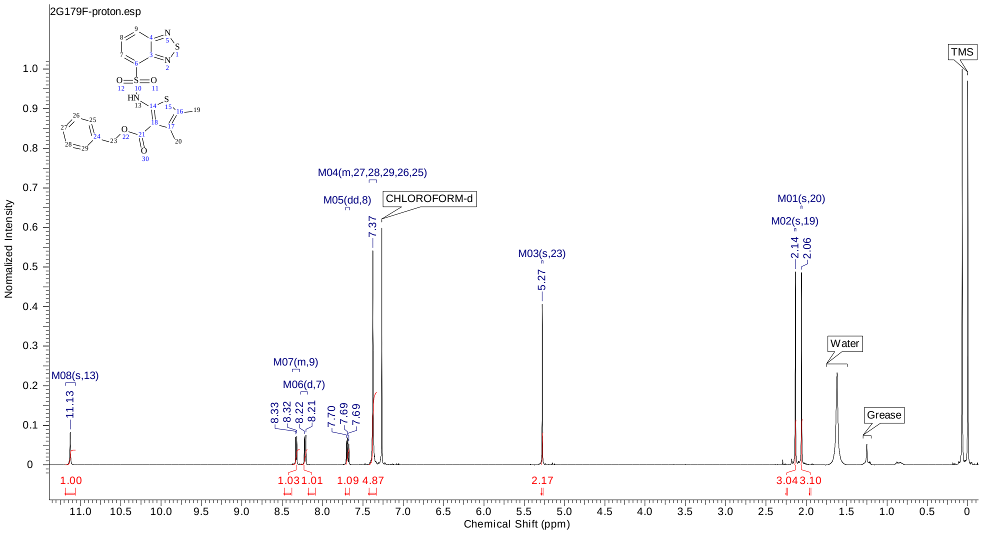

8/8/2022 2:50:47 PM

Formula	$C_{20}H_{17}N_3O_4S_3$	FW	459.5617

Acquisition Time (sec)	1.3005	Comment	Std carbon	Date	Jul 6 2022	Date Stamp	Jul 6 2022
File Name	C:\Users\Mycoahhh\Documents\NMR\michan\634\2G179F-carbon.fid\fid					Frequency (MHz)	125.69
Nucleus	13C	Number of Transients	140	Original Points Count	39649	Points Count	65536
Pulse Sequence	s2pul	Receiver Gain	30.00	Solvent	CHLOROFORM-d		
Spectrum Offset (Hz)	13192.3086	Spectrum Type	STANDARD	Sweep Width (Hz)	30487.80	Temperature (degree C)	30.000

^{13}C NMR (126 MHz, CHLOROFORM-d) d 164.9, 155.2, 148.7, 146.1, 135.5, 132.2, 130.3, 129.9, 128.7, 128.4, 128.4, 127.9, 127.1, 123.0, 114.1, 66.5, 14.4, 12.4

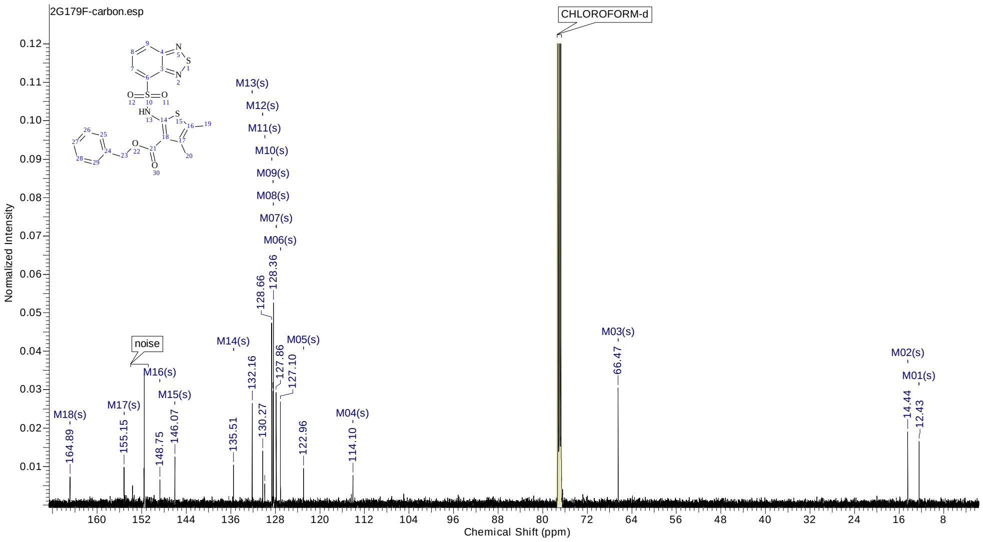

HRMS and LC-MS **2G179f**

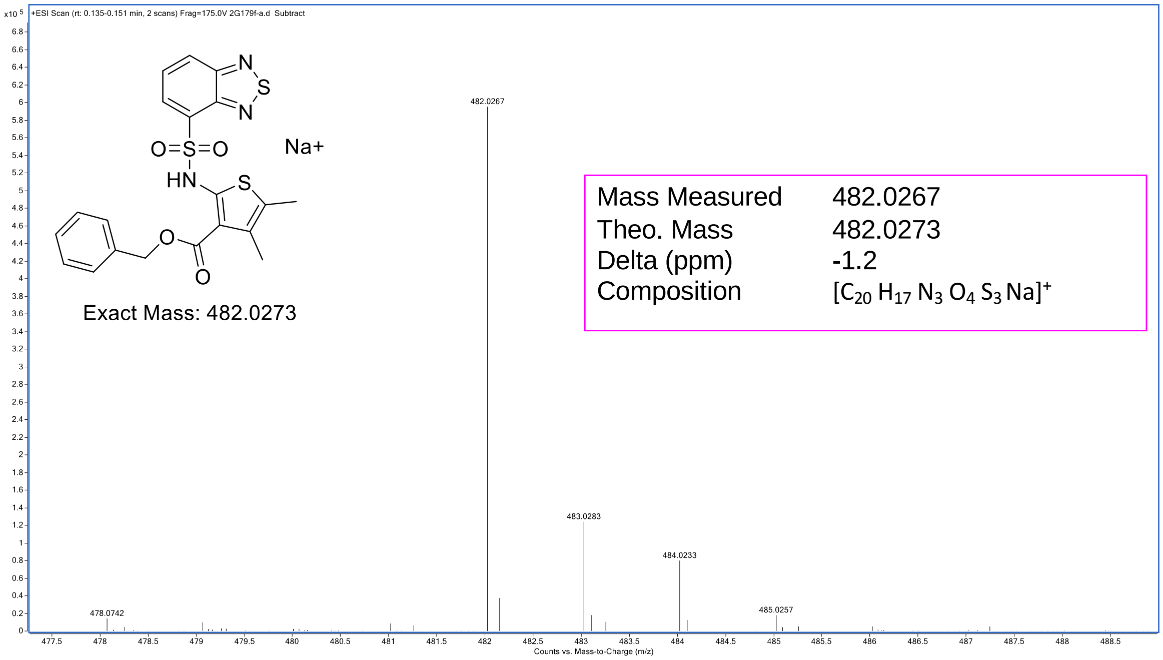

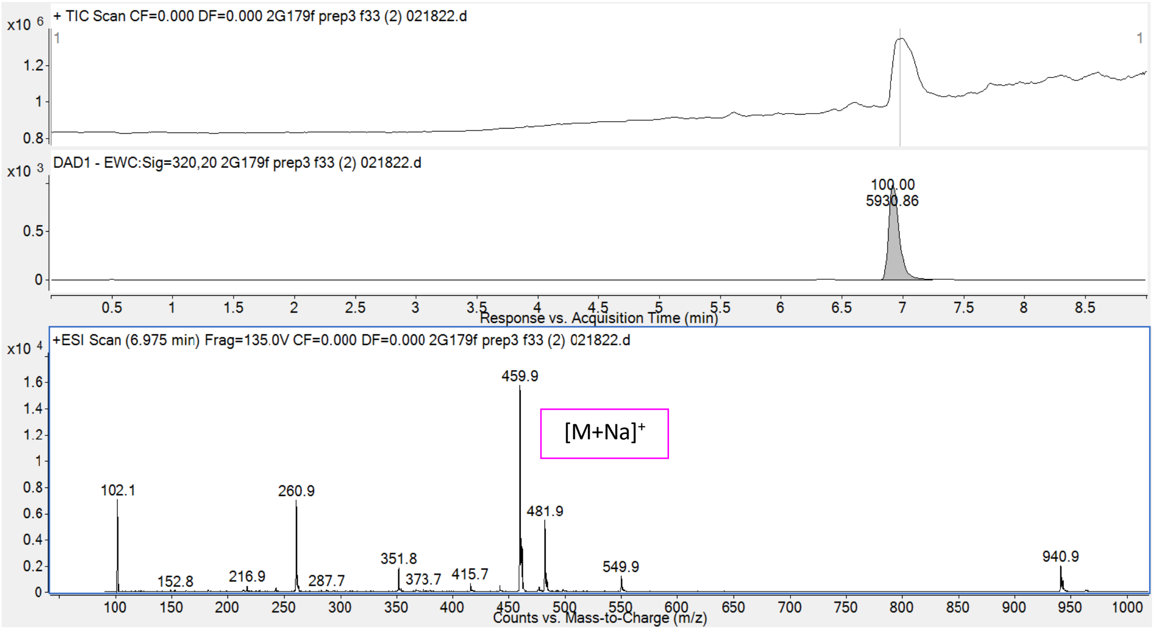

^{1}H, ^{13}C NMR **2G179g**

8/8/2022 2:53:21 PM

Formula	$C_{19}H_{15}N_3O_4S_3$	FW	445.5351

Acquisition Time (sec)	2.0486	Comment	Std proton	Date	Jul 6 2022	Date Stamp	Jul 6 2022
File Name	C:\Users\Mycoahhh\Documents\NMR\michan\634\2G179G-proton.fid\fid					Frequency (MHz)	499.83
Nucleus	1H	Number of Transients	16	Original Points Count	16415	Points Count	32768
Pulse Sequence	s2pul	Receiver Gain	32.00	Solvent	CHLOROFORM-d		
Spectrum Offset (Hz)	2994.2620	Spectrum Type	STANDARD	Sweep Width (Hz)	8012.82	Temperature (degree C)	30.000

^{1}H NMR (500 MHz, CHLOROFORM-d) d 10.92 (s, 1H), 8.36 (d, *J* = 6.36 Hz, 1H), 8.20 - 8.30 (m, 1H), 7.72 (dd, *J* = 7.09, 8.80 Hz, 1H), 7.45 (t, *J* = 7.83 Hz, 2H), 7.31 (t, *J* = 7.40 Hz, 1H), 7.06 (d, *J* = 7.58 Hz, 2H), 2.21 (s, 6H)

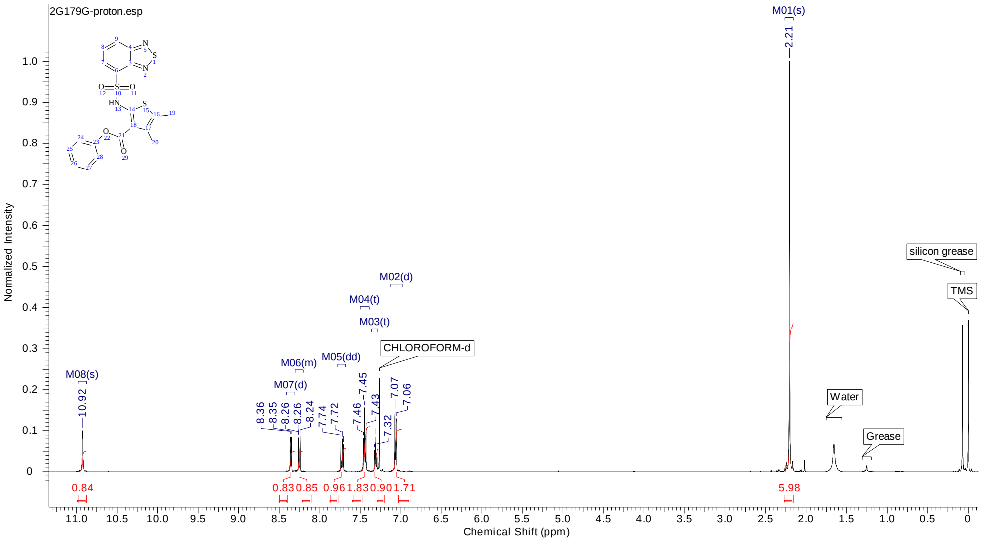

8/8/2022 2:52:33 PM

Formula	$C_{19}H_{15}N_3O_4S_3$	FW	445.5351

Acquisition Time (sec)	1.3005	Comment	Std carbon	Date	Jul 6 2022	Date Stamp	Jul 6 2022
File Name	C:\Users\Mycoahhh\Documents\NMR\michan\634\2G179G-carbon.fid\fid					Frequency (MHz)	125.69
Nucleus	13C	Number of Transients	120	Original Points Count	39649	Points Count	65536
Pulse Sequence	s2pul	Receiver Gain	30.00	Solvent	CHLOROFORM-d		
Spectrum Offset (Hz)	13192.3086	Spectrum Type	STANDARD	Sweep Width (Hz)	30487.80	Temperature (degree C)	30.000

^{13}C NMR (126 MHz, CHLOROFORM-d) d 163.6, 155.2, 149.8, 148.8, 147.3, 132.2, 130.3, 129.9, 129.6, 127.9, 127.2, 126.2, 123.3, 121.6, 113.5, 14.4, 12.5

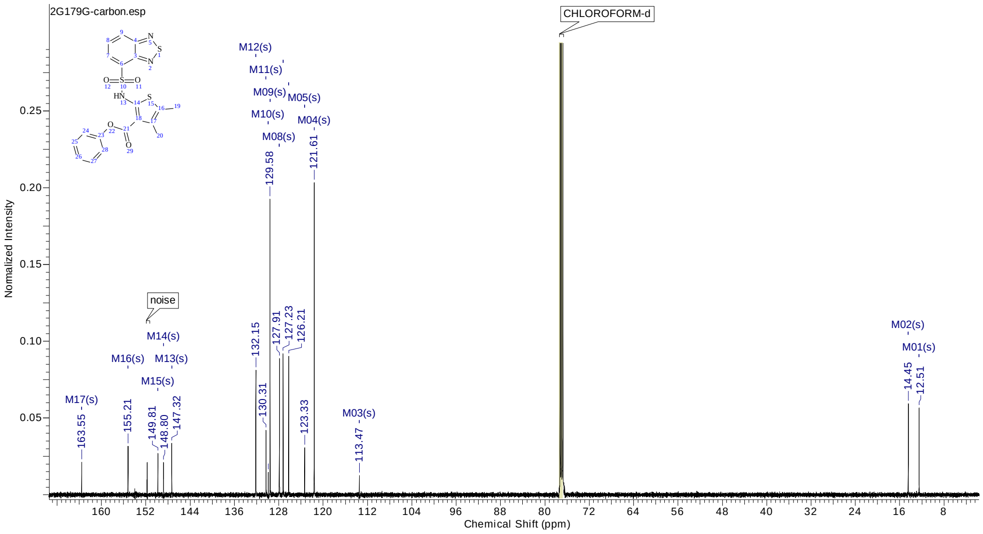

HRMS and LC-MS **2G179g**

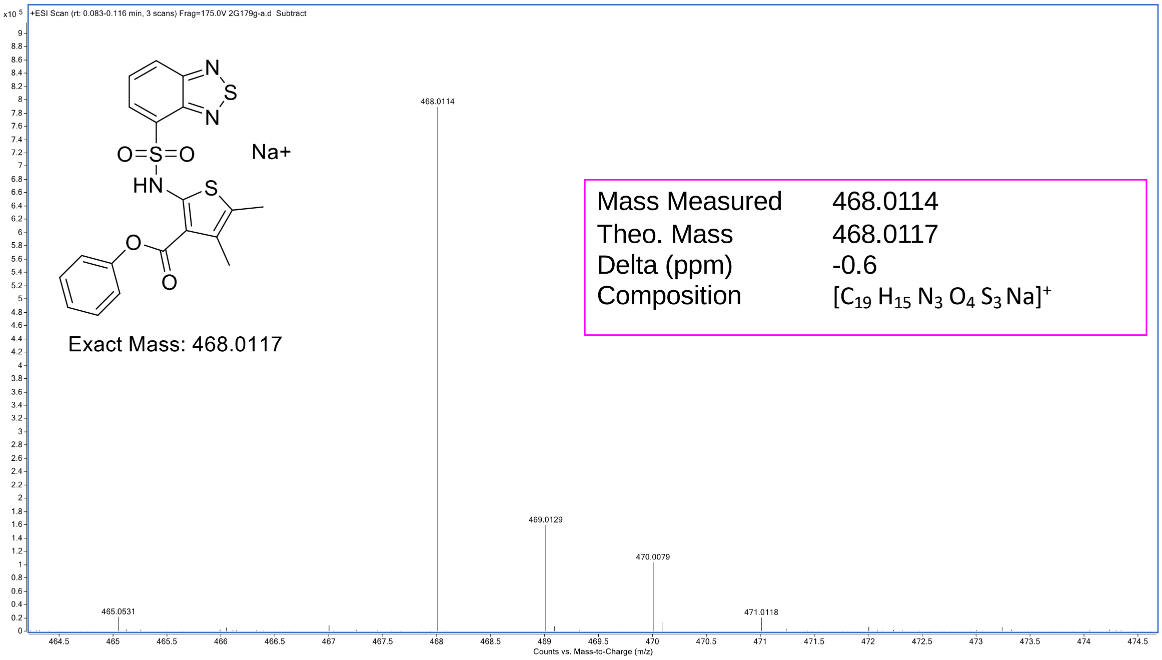

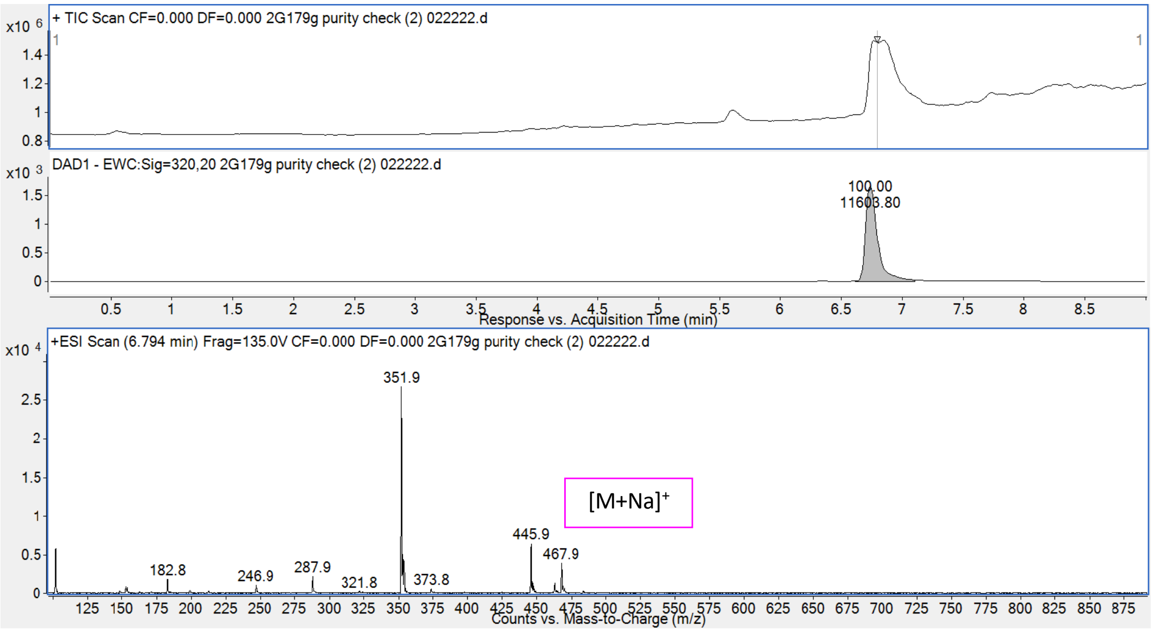

^{1}H, ^{13}C NMR 2G179h

8/8/2022 3:05:46 PM

Formula	$C_{17}H_{19}N_3O_4S_3$	FW	425.5455

Acquisition Time (sec)	2.0486	Comment	Std proton	Date	Jul 6 2022	Date Stamp	Jul 6 2022
File Name	C:\Users\Mycoahhh\Documents\NMR\michan\634\2G179H-proton.fid\fid					Frequency (MHz)	499.83
Nucleus	1H	Number of Transients	16	Original Points Count	16415	Points Count	32768
Pulse Sequence	s2pul	Receiver Gain	20.00	Solvent	CHLOROFORM-d		
Spectrum Offset (Hz)	2995.2400	Spectrum Type	STANDARD	Sweep Width (Hz)	8012.82	Temperature (degree C)	30.000

^{1}H NMR (500 MHz, CHLOROFORM-d) d 11.28 (s, 1H), 8.28 - 8.38 (m, 1H), 8.18 - 8.28 (m, 1H), 7.70 (dd, *J* = 7.09, 8.80 Hz, 1H), 2.14 (s, 3H), 2.04 (s, 3H), 1.52 (s, 9H)

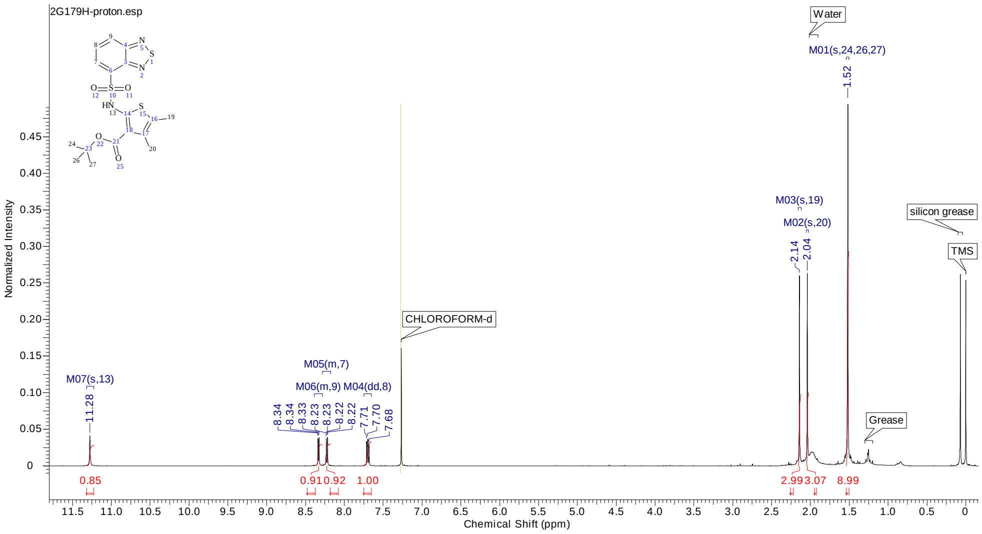

8/8/2022 2:58:36 PM

Formula	$C_{17}H_{19}N_3O_4S_3$	FW	425.5455

Acquisition Time (sec)	1.3005	Comment	Std carbon	Date	Jul 6 2022	Date Stamp	Jul 6 2022
File Name	C:\Users\Mycoahhh\Documents\NMR\michan\634\2G179H-carbon.fid\fid					Frequency (MHz)	125.69
Nucleus	13C	Number of Transients	128	Original Points Count	39649	Points Count	65536
Pulse Sequence	s2pul	Receiver Gain	30.00	Solvent	CHLOROFORM-d		
Spectrum Offset (Hz)	13192.3086	Spectrum Type	STANDARD	Sweep Width (Hz)	30487.80	Temperature (degree C)	30.000

^{13}C NMR (126 MHz, CHLOROFORM-d) d 164.5, 155.2, 148.9, 144.9, 132.3, 130.3, 130.0, 128.0, 127.0, 122.8, 115.8, 82.2, 28.2, 14.4, 12.4

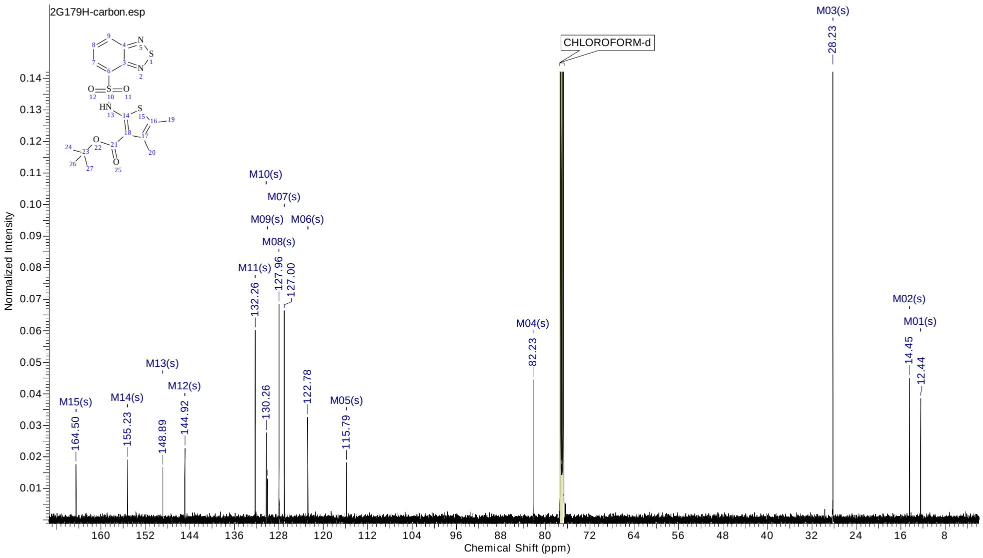

HRMS and LC-MS **2G179h**

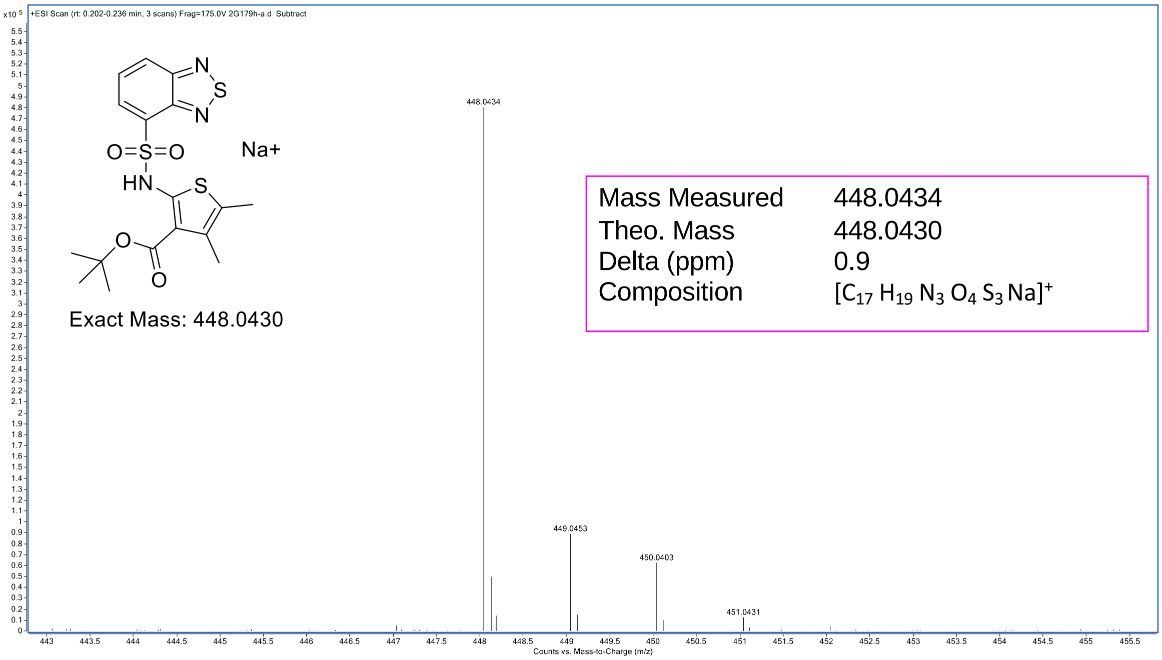

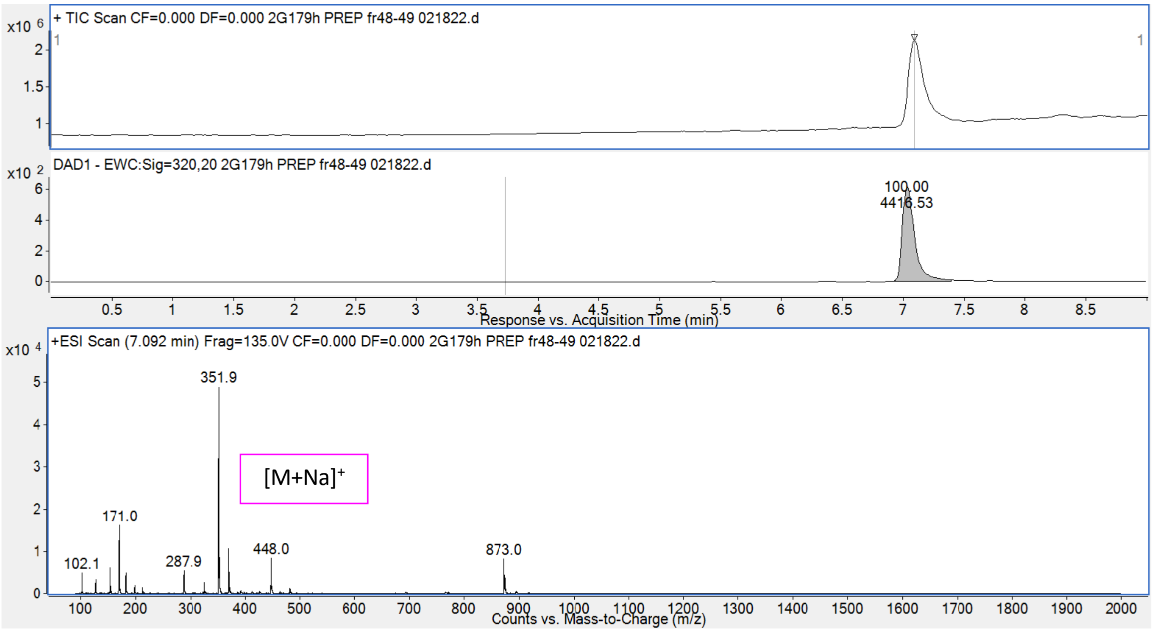

Materials and Methods: Biological Experiments

EV isolation by SEC. EV were isolated by UC as described above. The pellet was resuspended in 0.5 mL cold-filtered PBS. The resuspended pellet was applied on a qEV original size exclusion column (Izon Science) after rinsing the column with 17 mL PBS. We collected 13 fractions (0.4 mL of each) that flowed through the column after flushing the first 2.1 mL. SEC-EV (2nd to 5th fraction) and SEC-Contaminant (6th to 13th fraction) were collected, following the manufacturer's protocol. SEC-EV and SEC-Contaminant were concentrated by ultracentrifugation at 100,000 g_{avg} for 3 h in an SW28 rotor. The pellets were resuspended in 50 µL in cold-0.02 micron filtered PBS. All centrifugation steps were performed at 4 ˚C, and resultant samples were stored at -80 ˚C until use.

Measurement of the level of 634 in an EV preparation using LC-MS. EV sample 6 µL (1:4 diluted in PBS) was dissolved in 12 µL of acetonitrile, of which 15µL was injected into an LC-MS (Agilent 5420 Triple Quad mass spectrometry system) equipped with a Phenomenex C18 reverse-phase chromatography column (250 mm x 2.1 mm, 5µm) in the sample by multiple reaction monitoring (MRM) methods. The mobile phase consisted of acetonitrile (CAN) and water, both of which were acidified with trifluoroacetic acid at 0.01%. A gradient starting with 10:90 ACN: Water to 90:10 ACN: Water was utilized over 8 minutes to elute **634** at a flow rate of 0.4 mL per min at 30 ° C. The standard curve was prepared using 634 in DMSO at 10, 37, 111, and 333 nM. Six µL of each stock solution was dissolved in 12 µL of acetonitrile, of which 15 µL was injected into LC-MS, and **634** was eluted using the acetonitrile/water gradient.

RNA-seq and Data Analysis. mBMDCs were prepared from bone marrow cells harvested from femurs of C57BL/6 mice. mBMDCs (1.5×10^6 cells, 10^6 cells/mL × 1.5 mL) were treated with Veh (0.1% DMSO), **634** (5 µM) for 5 h, and then the total RNA was isolated using Quick-RNA™ Miniprep Kit (Zymo Research). Each group has triplicates. RNA-seq was performed by the sequencing core at the UC San Diego Institute for Genomic Medicine Genomics Center. Briefly, paired-ended sequencing was performed on the Illumina NovaSeq 6000. Reads were aligned to the mouse reference genome (mm10) using STAR (ver. 2.5.1), and mRNA expression levels were calculated per gene using RSEM (ver. 1.3.0). Genes were filtered if more than 24 out of 27 of the samples (including compound 634, there were a total of 8 testing compounds and DMSO in triplicated samples) had counts < 10. Raw counts were then quantile normalized and used as expression values in the subsequent analysis. Also, if mouse gene IDs corresponded with multiple human genes, the genes with the highest variance in expression values were kept [2]. A total of 11759 genes remained. Limma (linear models for microarray, using R-limma package) trend tests were used for differential analysis based on log2 expression values. The Benjamini-Hochberg procedure was applied to control the false discovery rate (FDR). A gene was considered significantly changed if FDR <0.05 and fold change>2. If log2 fold change in expression for a test compound vs control was greater than 0, it was said to be up-regulated; otherwise, it was down-regulated. RNA-seq data have been deposited in ArrayExpress, https://www.ebi.ac.uk/arrayexpress/ (accession no. E-MTAB-12377) [3].

RT-qPCR. RNA was isolated from mBMDCs derived from C57BL/6 mice (1.5×10^6 cells, 10^6 cells/mL × 1.5 mL) treated with Veh (0.1% DMSO), **634** (5 µM) for 5 h using Quick-RNA™ Miniprep Kit (Zymo Research) and was reverse-transcribed using iScript (Bio-Rad). qPCR analyses using Taqman Gene Expression Assay (Thermo Fisher Scientific) were performed by CFX-Connect Real-Time System (Bio-Rad). Data were normalized to *Gapdh* expression. PCR primer pairs were as follows; *Htr7* (#Mm00434133_m1), *Cdh1* (#Mm01247357_m1), *Cdhr1* (#Mm00499982_m1), *Ppm1e* (#Mm07302251_m1), and *Gapdh* (Pre-Developed TaqMan Assay Reagents mouse *Gapdh* # 4352661) from Thermo Fisher Scientific.

Reference

(1) Shukla NM, Sato-Kaneko F, Yao S, Pu M, Chan M, Lao FS, et al. A Triple High Throughput Screening for Extracellular Vesicle Inducing Agents With Immunostimulatory Activity. Front Pharmacol. 2022;13: 869649. doi:10.3389/fphar.2022.869649

(2) Miller JA, Cai C, Langfelder P, Geschwind DH, Kurian SM, Salomon DR, et al. Strategies for aggregating gene expression data: The collapseRows R function. Bmc Bioinformatics. 2011;12: 322. doi:10.1186/1471-2105-12-322

(3) T. Hayashi, ArrayExpress. https://www.ebi.ac.uk/arrayexpress/. (Deposited 30 October 2022).
